# HAP40 is a conserved central regulator of Huntingtin and a potential modulator of Huntington’s disease pathogenesis

**DOI:** 10.1371/journal.pgen.1010302

**Published:** 2022-07-19

**Authors:** Shiyu Xu, Gang Li, Xin Ye, Dongsheng Chen, Zhihua Chen, Zhen Xu, Moretti Daniele, Sara Tambone, Alessandra Ceccacci, Licia Tomei, Lili Ye, Yue Yu, Amanda Solbach, Stephen M. Farmer, Erin Furr Stimming, George McAllister, Deanna M. Marchionini, Sheng Zhang

**Affiliations:** 1 The Brown Foundation Institute of Molecular Medicine, McGovern Medical School at the University of Texas Health Science Center at Houston (UTHealth), Houston, Texas, United States of America; 2 Department of Translational and Discovery Research, IRBM SpA, Pomezia (RM), Italy; 3 Programs in Genetics and Epigenetics and Neuroscience, The University of Texas MD Anderson Cancer Center UTHealth Graduate School of Biomedical Sciences (MD Anderson UTHealth GSBS), Houston, Texas, United States of America; 4 Program in Biochemistry and Cell Biology, The University of Texas MD Anderson Cancer Center UTHealth Graduate School of Biomedical Sciences (MD Anderson UTHealth GSBS), Houston, Texas, United States of America; 5 Department of Neurology, HDSA Center of Excellence, McGovern Medical School at the University of Texas Health Science Center at Houston (UTHealth), Houston, Texas, United States of America; 6 CHDI Management/CHDI Foundation, 350 Seventh Ave, New York, New York, United States of America; 7 Department of Neurobiology and Anatomy, McGovern Medical School at the University of Texas Health Science Center at Houston (UTHealth), Houston, Texas, United States of America; The University of North Carolina at Chapel Hill, UNITED STATES

## Abstract

Perturbation of huntingtin (HTT)’s physiological function is one postulated pathogenic factor in Huntington’s disease (HD). However, little is known how HTT is regulated *in vivo*. In a proteomic study, we isolated a novel ~40kDa protein as a strong binding partner of *Drosophila* HTT and demonstrated it was the functional ortholog of HAP40, an HTT associated protein shown recently to modulate HTT’s conformation but with unclear physiological and pathologic roles. We showed that in both flies and human cells, HAP40 maintained conserved physical and functional interactions with HTT. Additionally, loss of HAP40 resulted in similar phenotypes as HTT knockout. More strikingly, HAP40 strongly affected HTT’s stability, as depletion of HAP40 significantly reduced the levels of endogenous HTT protein while HAP40 overexpression markedly extended its half-life. Conversely, in the absence of HTT, the majority of HAP40 protein were degraded, likely through the proteasome. Further, the affinity between HTT and HAP40 was not significantly affected by polyglutamine expansion in HTT, and contrary to an early report, there were no abnormal accumulations of endogenous HAP40 protein in HD cells from mouse HD models or human patients. Lastly, when tested in *Drosophila* models of HD, HAP40 partially modulated the neurodegeneration induced by full-length mutant HTT while showed no apparent effect on the toxicity of mutant HTT exon 1 fragment. Together, our study uncovers a conserved mechanism governing the stability and *in vivo* functions of HTT and demonstrates that HAP40 is a central and positive regulator of endogenous HTT. Further, our results support that mutant HTT is toxic regardless of the presence of its partner HAP40, and implicate HAP40 as a potential modulator of HD pathogenesis through its multiplex effect on HTT’s function, stability and the potency of mutant HTT’s toxicity.

## Author summary

Abnormal polyglutamine expansion in huntingtin (HTT) protein causes neurodegenerative Huntington’s disease (HD). Given the complex and still unclear pathogenic mechanisms underlying HD, there is great interest in targeting HTT as a therapeutic strategy against HD. Accordingly, it is important to understand how HTT is normally regulated. In a proteomic study in *Drosophila*, we isolated a novel protein as a strong binding partner of fly HTT homolog and demonstrated that it was the functional counterpart of mammalian HAP40, a protein known to modulate HTT’s conformation but with unclear physiological and pathologic roles. We found that the physical and functional interactions of HTT with HAP40 were conserved from flies to human cells, and HAP40 was essential for HTT’s normal functions and protein stability, while in the absence of HTT, HAP40 was quickly cleared through the proteasome. Further, polyglutamine expansion in HTT protein did not affect its affinity for HAP40 or cause abnormal accumulation of HAP40 in HD cells. Lastly, we observed that HAP40 could modulate HD-associated neurodegeneration in fly models that expressed full-length mutant HTT. Together, our study demonstrates that HAP40 is a highly conserved and essential regulator of HTT and a potential candidate for achieving specific HTT-lowering in HD cells.

## Introduction

Huntington’s disease (HD) is a neurodegenerative disorder caused by an abnormal expansion of a glutamine tract (polyQ) near the N-terminus of huntingtin (HTT) protein [1]. Despite its simple genetic cause, HD has a rather complex etiology that remains to be elucidated [2,3]. In particular, although HTT gene is broadly expressed throughout the brain, HD preferentially destroys medium spiny neurons in the striatum and pyramidal neurons in the cortex [4–8]. This and other evidence have led to the hypothesis that longer polyQ tracts not only confer a dominant toxicity, but also perturb HTT’s normal physiological functions, and these combinatorial insults together lead to selective neuronal loss over time [9,10]. Consequently, there is great interest in targeting HTT itself to either specifically eliminate mutant HTT or to lower the levels of total HTT as a therapeutic strategy against HD [11]. A clear understanding of how endogenous HTT is regulated *in vivo*, including its protein stability and its normal cellular functions, are critical both for elucidating HD etiology and for identifying effective drug targets.

HTT is a widely expressed, large ~350 kDa protein [4,6,8]. In mouse, it is essential for early embryogenesis and for neuronal survival in postnatal brain [12–16]. Structurally, HTT is composed primarily of HEAT repeats, an anti-parallel double-helix motif implicated in protein-protein interaction [17,18], leading to the proposal that HTT is a scaffold to mediate protein interactions and integrate multiplex cellular responses [9,19]. Indeed, HTT is involved in a diverse set of cellular processes including transcription, trafficking and autophagy [10,20]. However, despite extensive studies, it is still unclear how HTT itself is regulated *in vivo*.

As a powerful genetic model, *Drosophila* has been invaluable in discovering and dissecting conserved signaling pathways and modeling human diseases including HD [21]. Previously, we have characterized *Drosophila* HTT homolog (*dhtt*) [22], which has relatively low homology at the amino acid level with human HTT [23]. But resembling HTT, *dhtt* also encodes a large protein (3,511 amino acids) consisting primarily of predicted HEAT repeats [18,22,23]. Importantly, beyond their similarity in large protein size and secondary structure, *dhtt* is also involved in similar cellular processes as its mammalian counterparts. For example, *dhtt* can functionally replace HTT in rescuing the axonal trafficking and misaligned mitotic spindle in mouse cortical neurons [24,25] and plays a similar conserved role in selective autophagy [26,27], supporting *Drosophila* as a relevant *in vivo* organism to functionally dissect HTT.

Considering the structural and functional conservation of HTT from flies to humans, we hypothesize that the core regulators of HTT, especially those with close physical interactions with HTT, should be conserved between these two evolutionary distant species. Indeed, among the large number of HTT-associated proteins (HAPs) identified from various biochemical and proteomic studies, most have fly homologues [10,28–33]. However, few of them have been validated *in vivo* and the central regulators of HTT remain largely obscure.

Accordingly, we carried out proteomic studies to directly isolate dHtt-associated proteins (dHaps) from fly tissues, from which we identified a novel 40kDa protein as a specific and perhaps the strongest binding partner of dHtt. This 40kDa protein, encoded by a previously uncharacterized gene *cg8134*, shares notable sequence similarity with HAP40, a known HTT binding partner in mammals. HAP40 was first isolated from rat brain homogenate as an interactor of endogenous HTT [34], and was shown later in a proteomic study in mouse brains as the “most significantly correlated” with HTT in their protein levels [31]. Consistently, HAP40 was found to bind HTT tightly at 1:1 molar ratio [35]. More recently, HAP40 was found to be important in regulating HTT’s conformation [36]. Importantly, a ~10 fold increase of HAP40 levels were observed in primary fibroblasts and striatal tissues from HD postmortem brains as compared to healthy controls [35]. Despite these strong biochemical and structural evidence, there is no reported study on HAP40’s physiological roles in any animal settings, and its functional relationship with HTT and its potential involvement in HD pathogenesis remain unclear.

We found that *in vitro*, the 40kDa protein encoded by *cg8134* was capable of physically interacting with human HTT. Conversely, when expressed in flies, human HAP40 could rescue the phenotypes of *cg8134-*null mutants, supporting *cg8134* as the fly orthologue of HAP40. Accordingly, we renamed *cg8134* as *dhap40* (*Drosophila* Hap40). At whole animal levels, flies lacking *dhap40* showed similar loss of function phenotypes as *dhtt* null (*dhtt-ko*) mutants, suggesting that *dhtt* and *dhap40* play similar or overlapping physiological roles. Strikingly, there was an almost complete loss of endogenous dHap40 protein in *dhtt* null flies. Conversely, the levels of endogenous dHtt were significantly reduced in *dhap40* knockout flies. Importantly, a similar mutual reliance between HTT and HAP40 is conserved in mammalian cells. Further, polyQ expansion in HTT did not affect its binding with HAP40, HAP40 overexpression were not toxic in neuronal tissues in flies, and there was no apparent increase of HAP40 levels in HD cells. Lastly, when tested with fly models of HD, HAP40 significantly altered the levels of overexpressed human full-length HTT protein, but only mildly modulated neurodegeneration induced by full-length mutant HTT and no effect on mutated HTT exon 1. Collectively, these findings support that HAP40 is an essential and conserved regulator of HTT, and mutant HTT is toxic regardless of its binding with HAP40.

## Results

### Affinity purification of interacting partners for endogenous dHtt from *Drosophila*

**Fig 1:**
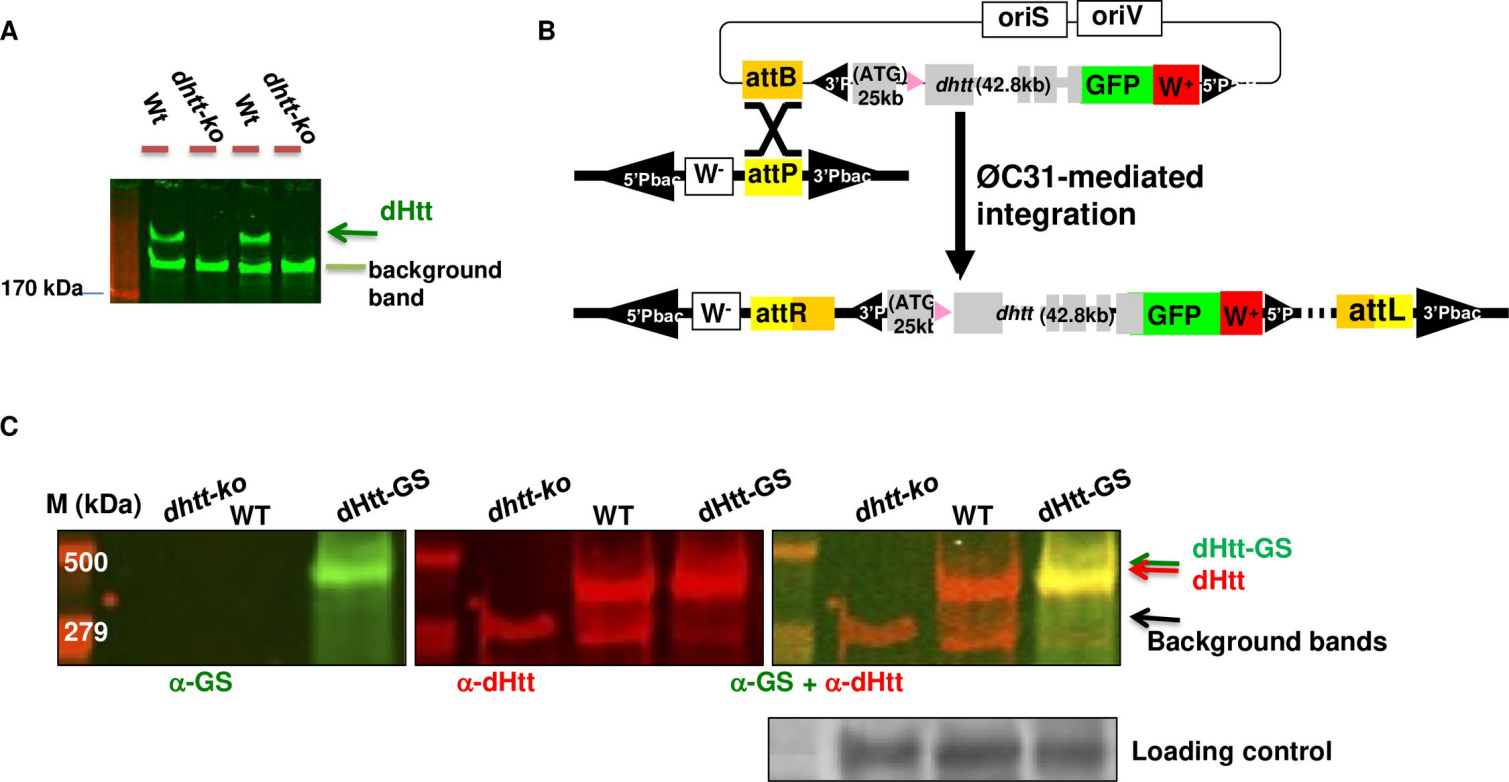
dHtt expression and genomic tagging of dHtt. (A) Western blot analysis of endogenous dHtt expression in *Drosophila* embryos, revealed with anti-dHtt antibody. A single ~400kDa dHtt band is present in wildtype embryos and absent in age-matched *dhtt-ko* mutants. The lower background band served as loading control, as noted. (B) Schematics for genome-tagging *dhtt* gene at its C-terminus with eGFP, which includes an ~80kb genome region covering *dhtt* gene, including about 25kb upstream of the first coding exon, 42.8 kb genome region covering all the coding exons and about 12kb 3’-untranslated region (UTR). eGFP was fused in-frame to the C-terminus of encoded dHtt protein before the stop codon and 3’ UTR. The genome fusion constructs were cloned into pacman vector through the recombineering method, and subsequently integrated into the preselected attP site in the fly genome through the phiC31-integrase mediated transgene approach (see Methods). (C) Western analysis for the expression of dHtt-GS-TAP fusion protein from pacman-*dhtt-GS-TAP* transgenic flies (dHtt-GS), or controls of wildtype (WT) and *dhtt-ko* mutants, as indicated. The GS-TAP tag contains two Protein G modules, a TEV protease cleavage site, and a streptavidin binding peptide (SBP). The whole-protein extracts from the indicated genotypes were probed simultaneously with anti-SBP (green band in left panel) to detect the GS-TAP tag and anti-dHtt (red bands in the middle panel) antibodies, followed by anti-βTubulin antibody for loading control. The dHtt-GS-TAP fusion protein was only detected in the *dhtt*-GS transgenic flies and absent in controls of WT (middle lane) and *dhtt-ko* (left lane) flies. The overlaying image (right panel) for both anti-SBP and anti-dHtt staining showed that the dHtt-GS-TAP was expressed as full-length fusion protein (orange band) with slightly larger size than endogenous dHtt.

**Fig 2:**
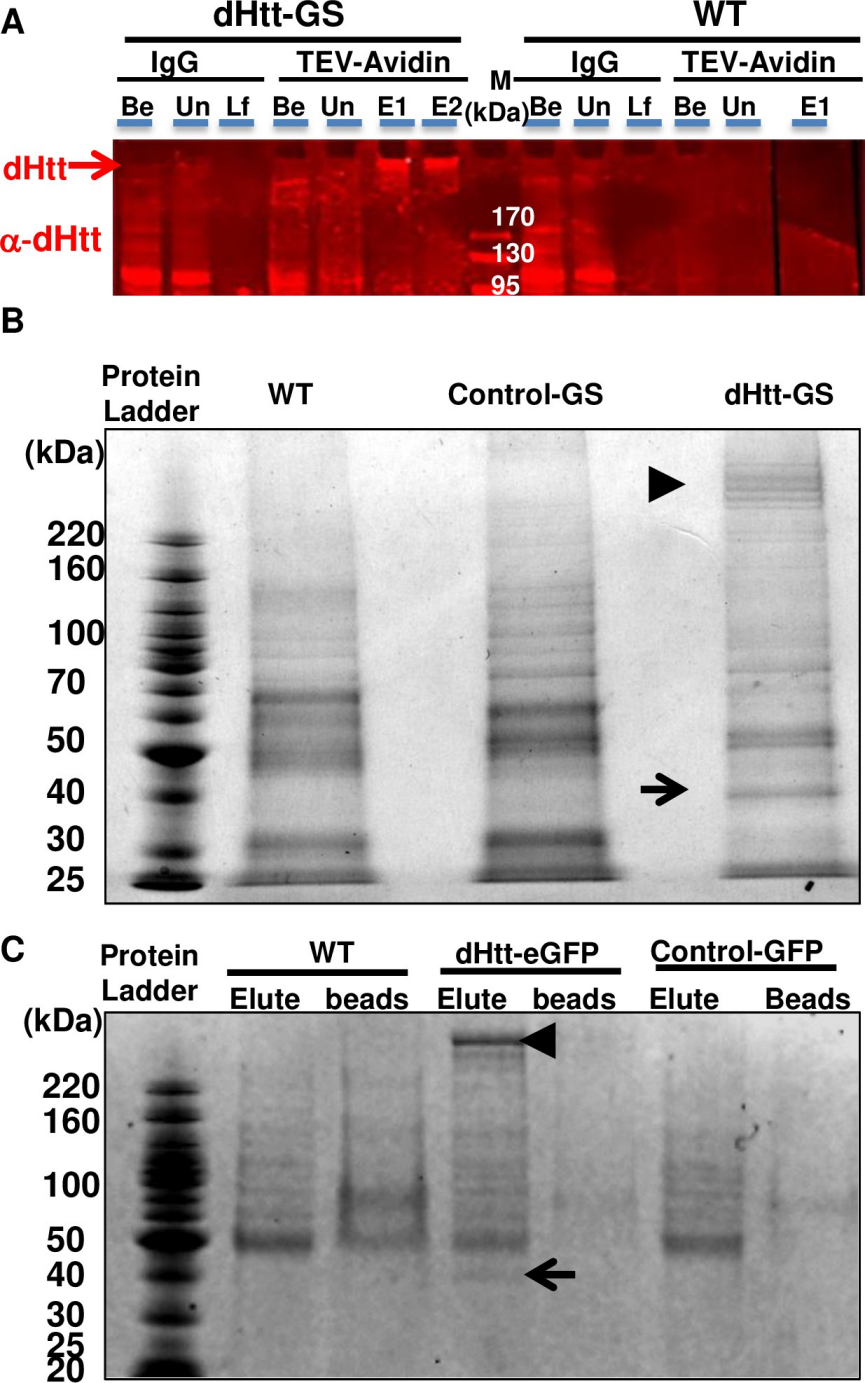
Affinity-purification of endogenous dHtt and dHtt-associated proteins from *Drosophila*. (A) Western blot analysis of sequential purification steps for dHtt-GS-TAP fusion and its associated proteins from flies. Whole embryos extracts from wildtype control (WT) or flies transgenic for pacman-dhtt-GS-TAP (dHtt-GS) were processed in parallel through the following purification steps: (1) IgG-pulldown against Protein G modules in the GS-TAP tag; (2) TEV protease cleavage of the TEV recognition site in the GS-TAP tag to release dHtt and associated proteins from IgG beads; (3) Second pull-down with Streptavidin beads against the SBP within the GS-TAP tag; (4) final elution of dHtt and dHtt-associated proteins from Streptavidin beads with biotin. When possible, equal volumes of protein extracts or agarose slurry from the following purification steps were analyzed, as annotated in the figure panel (from the left to right): Be, whole embryo extracts *before* incubation with IgG beads; Un, *unbound* protein samples after depletion of dHtt-GS with IgG-conjugated agarose beads from whole embryo extracts; Lf, *leftovers* on IgG beads after TEV cleavage; Be, elute from IgG beads after TEV cleavage, *before* incubating with Streptavidin-conjugated agarose beads; E1 and E2: final *eluate* 1 and 2 fractions released from Streptavidin beads with biotin solutions. Samples from each of the above purification steps were processed for SDS-PAGE analyses and probed with rabbit α-dHtt antibody. Note that the quantity of endogenous dHtt protein in the crude protein extracts from the pacman-dhtt-GS-TAP transgenic flies was barely detectable (lane 1, labeled as “Be” in left panel), and became significantly enriched in the final eluates (arrows in E1, E2). The same band was absent from WT control (right panel). M: protein ladder *marker*, with their sizes labeled. (B and C) SDS-PAGE analysis of affinity-purified dHtt and dHtt-associated proteins isolated from transgenic flies carrying (B) pacman-*dhtt*-GS-TAP (dHtt-GS), or (C) pacman-*dhtt*-eGFP, visualized with Coomassie blue staining. The following controls were processed in parallel: wildtype (WT) non-transgenic flies; controls for pacman flies carrying in-frame fusion with an unrelated protein of (B) GS-TAP (Control-GS) or (C) eGFP (Control-GFP) tags, as indicated. Note the co-purification of a prominent ~40kDa protein (arrows) with the ~400 kDa dHtt (arrowheads) specifically from (B) dHtt-GS or (C) dHtt-GFP flies only, but not from either of the controls. In (B), several large bands were present around the ~400 kDa range (arrowhead) specifically in dHtt-GS sample, which likely were partially degraded dHtt protein generated during multi-step TAP purification procedures. In (C), after final eluting from GFP-nanobody agarose beads with 10mM glycine (PH 2.5), both the eluates (Elute) and the post-elution agarose beads (beads) were processed for SDS-PAGE analysis.

We first examined the expression patterns of endogenous dHtt protein by Western blot analysis, which revealed a single protein product of about 400kDa that was present in wildtype but absent in *dhtt-ko* flies (Fig 1A). Given its overall low expression levels (Fig 2A), to achieve effective pulldown of its binding partners under physiological conditions, we created two genome-tagging lines containing in-frame fusion of eGFP or GS-TAP tags at the C-terminus of the encoded dHtt protein. Specific anti-GFP antibodies, including the nanobody [37], are available for efficient pull down of GFP-tagged protein, while GS-TAP is a tag optimized for tandem-affinity-purification (TAP) of target proteins from *Drosophila* tissues or cultured cells [38, 39]. The GFP and GS-TAP tags have no overlap sequences, thus allowing affinity-purification (AP) of dHtt and its associated proteins (dHaps) via two complementary approaches. Using pacman, an optimized BAC transgene approach [40], we established the genome-tagging lines for *dhtt-eGFP* and *dhtt-GS-TAP* (Fig 1B). The ~83Kb BAC constructs carried the full coding and regulatory regions of *dhtt* gene, thereby enabling the expression of the tagged *dhtt* transgene under the control of its native regulation elements, and the levels and expression patterns of the tagged dHtt protein mirroring that of endogenous dHtt. Subsequent characterization of the pacman transgenic flies confirmed the expression of the tagged dHtt-eGFP and dHtt-GS-TAP proteins at the expected sizes and levels comparable to that of endogenous dHtt (Figs 1C and S1). Further, despite their relatively low abundance in whole animal homogenates, dHtt-GS-TAP fusion was significantly enriched to relatively high purity through the TAP sequential purification (Fig 2A and 2B). Similarly, a single-step pulldown by anti-GFP nanobody highly enriched dHtt-GFP from homogenates (Fig 2C). The *dhtt* genome tagging lines thus allow convenient isolation of dHaps under physiological setting, avoiding artifacts often associated with protein mis-expression and overexpression.

### CG8134 is a strong and specific binding partner of endogenous dHtt

Using *dhtt* genome-tagging flies, we optimized and then carried out multiple rounds of large-scale affinity purifications for dHtt associated proteins from whole embryo homogenates, which were prepared from overnight collections of fly embryos of appropriate genotypes (see Materials and Method for details). To eliminate false positives common with purification procedures or with promiscuous binding to the GS-TAP or GFP tags, we included three sets of controls in parallel experiments: (1) wildtype flies carrying no transgenes, (2) an independent pacman transgenic line expressing an unrelated gene with a C-terminal GS-TAP tag, and (3) another pacman transgenic line expressing the same unrelated gene with a C-terminal GFP tag.

**Fig 3:**
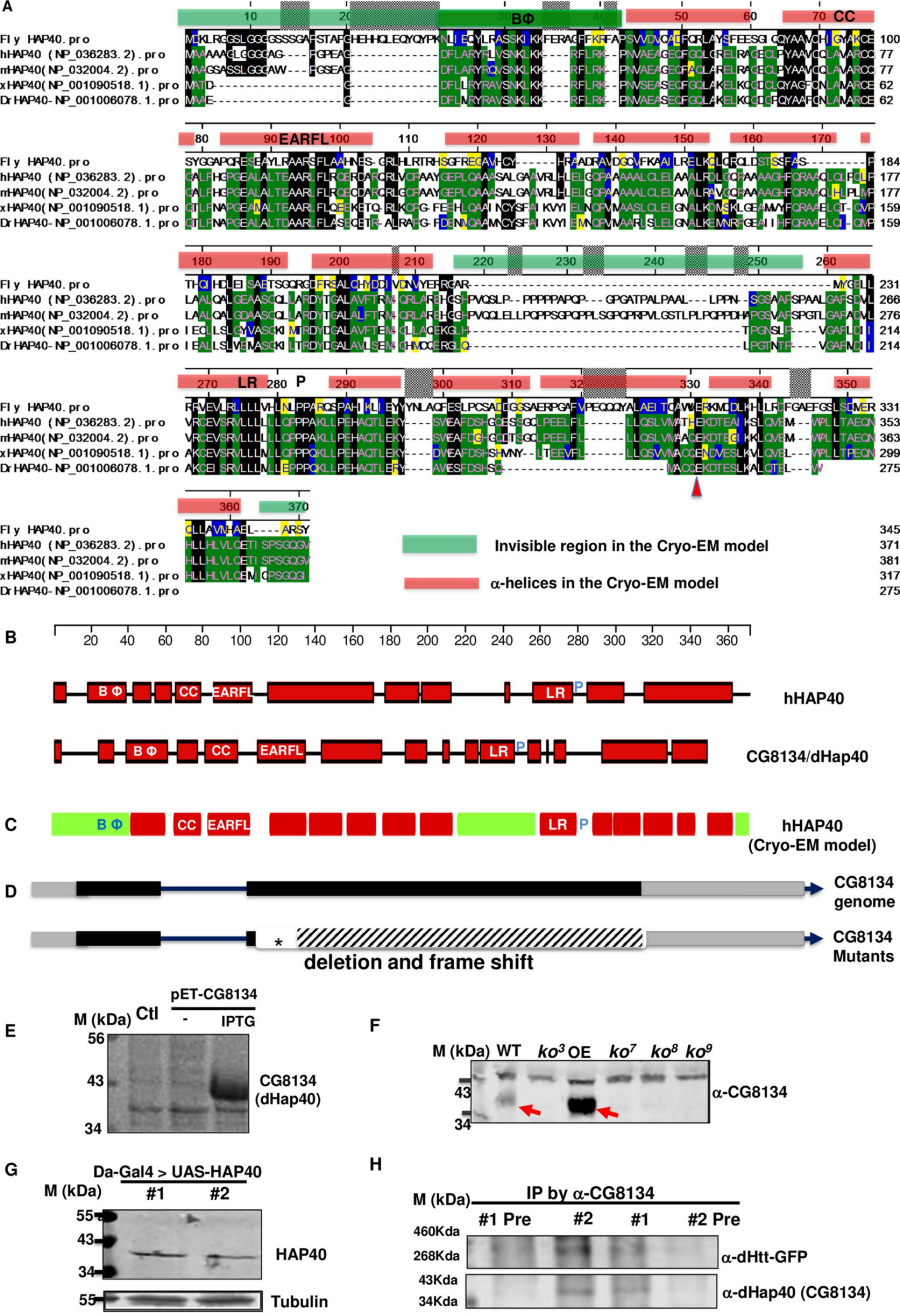
CG8134 encodes *Drosophila* Hap40 homologue. (A) Sequence alignment between CG8134/dHap40 and the following vertebrate HAP40 homologs: human (hHAP40/F8a1), mouse (mHAP40/F8a), xenopus (xHAP40) and zebra fish *Danio rerio* (DrHAP40). Genebank ID of these proteins were indicated. The scale bar above the alignment refers to amino acid position from human HAP40 (NP_036283). Amino acid similarities are annotated in color in the following order in the alignment: (1) black box highlights amino acids that are identical to that in CG8134/dHap40; (2) green boxes are those identical to those in human HAP40; (3) Yellow boxes are those with similar chemical property as those in human HAP40; (4) blue box highlights amino acids that are with similar chemical property as in CG8134/dHap40. The red bars above the alignment cover the sequences of the predicted 14 α-helices and the green bars those invisible in the Cryo-EM model. (B) Schematics of the predicted secondary structures in human HAP40 (top) and CG8134/dHap40 (bottom) proteins using the Garnier-Robson structure prediction module (Protean program by DNASTAR), drawn in scale (top) of human HAP40. Red boxes represent the predicted α-helices. BΦ: the N-terminal α-helices enriched with basic and hydrophobic amino acids, which is part of the N-terminal invisible region in the Cryo-EM model (see Fig 3C below); CC: the α-helices containing two conserved cysteines; EARFL: α-helices with a conserved stretch of E,A,R,F and L amino acids; LR: α-helices with leucine-repeat; P: proline linker after the LR α-helices. (C) Schematics of the secondary structures in human HAP40 protein predicted from the Cryo-EM study. Red rectangles correspond to the predicted 14 α-helices and the green rectangles are regions invisible in the Cryo-EM model. (D) Schematics of genomic structure of *cg8134/dhap40* gene and the mutant alleles created in the study. *dhap40* is a X-linked gene composed of one intron (solid line) and two coding exons (grey boxes: untranslated regions; black boxes: coding regions), with arrowhead indicting the orientation of the encoded protein from 5’ to 3’ ends. Star annotates the locations of the molecular lesions for each of the characterized *dhap40* alleles. Blank area indicates the region of small insertions and deletions and shaded area the induced frameshift in the encoded protein. (E) SDS-PAGE and Coomassie blue staining of protein lysates from *E*. *coli*. A protein product of 40kDa size was produced only in cells transformed with a pET protein expression plasmid containing full-length *cg8134* cDNA without (lane 2) or with IPTG induction (lane 3) but not in control transformed with an empty protein expression vector alone (lanes 1). (F) Western Blot analysis of CG8134/dHap40 expression in adult flies from wildtype (WT), four *dhap40* mutant (*ko3*, *ko7*, *ko8* and *ko9*) alleles, and a dHap40 overexpression line (OE) carrying a UAS-*cg8134* transgene directed by a strong Actin-Gal4 driver. Note that a 40kDa band (left red arrow) corresponding to endogenous CG81344/dHap40 was detected in WT and present at significantly higher levels in the OE line (right red arrow), but was completely absent in the four *dhap40* mutant lines. (G) Western Blot analysis of ectopically expressed human HAP40, detected as a 40kDa protein, from homogenates of two fly lines carrying a UAS-F8A1/HAP40 transgene direct by *daughterless*-Gal4 driver. (H) co-IP of endogenous dHtt by dHap40 antibodies. Fly homogenates from dHtt-eGFP flies were incubated with two independent anti-dHap40 (anti-CG8134 # 1 and #2) antibodies, or with their corresponding pre-immunization sera (#1 Pre and #2 Pre) as controls. The immunoprecipitates were probed with anti-GFP or anti-dHap40 antibodies after SGS-PAGE separation, as indicated.

In all the pull-down assays, a prominent ~40kDa band was co-purified with both dHtt-eGFP and dHtt-GS-TAP, but not in either of the controls (arrows in Fig 2B and 2C). The relatively high abundance of this ~40kDa protein in independent dHtt-pull down experiments suggests that it is a specific and likely the strongest binding partner for endogenous dHtt. Mass spectrometry of the 40kDa band revealed it is the predicted protein product of a previously uncharacterized gene *cg8134* (Fig 3A).

### CG8134 encodes the fly HAP40 ortholog

BLAST search of the protein encoded by *cg8134* revealed notable similarity with HAP40 homologs from multiple vertebrate species (Fig 3A). The recent Cryo-EM study on the structure of the HTT/HAP40 complex noted that homology-based search failed to identify a HAP40 homolog in *Drosophila* [36]. However, the homology between CG8134 and HAP40 exists throughout most of the proteins, and are particularly prominent in two ~50 amino acid (a.a.) long stretches at their N- or C-terminal regions, where there are about ~40% identical and ~50% similar amino acids between CG8134 and human HAP40 proteins (Figs 3A and S2A). Importantly, the Cryo-EM study revealed that the N- and C- regions of HAP40 mediate its direct physical interactions with HTT [36], raising an attractive possibility that the similar conserved regions in CG8134 also mediate its binding with dHtt in *Drosophila*.

The homology is not just limited to amino acid sequence, but also to their predicted secondary structures. In particular, when analyzed using the same modeling parameters, both CG8134 and human HAP40 are predicted to be composed mainly of α-helices that are distributed in similar patterns throughout the two proteins (Fig 3B). Importantly, these predicted α-helices also align remarkably well with the 14 α-helices identified in the Cryo-EM study [36].

Sequence alignment across species also revealed several intriguing structural features suggestive of potential functional importance (Fig 3A–3C). For example, the central region of human HAP40, predicted as unstructured from the sequence-based prediction (Fig 3B), is invisible in the model from the Cryo-EM study (the middle green bar in Fig 3A and 3C) and absent in both CG8134 and the HAP40 homologs in zebrafish and frogs (Fig 3A), consistent with the findings that it is dispensable for HTT binding [36]. In contrast, although the very N-terminus of HAP40 is also invisible in the Cryo-EM study, it contains a ~20 amino acid-long stretch which we named as “BΦ” motif for its unusually high content of basic and hydrophobic residues, a feature that is highly conserved (Fig 3A–3C). In particular, in human HAP40, the BΦ motif contains eight strong basic (K,R) and eight hydrophobic (A,I,L,F,W,V) amino acids (a.a. 21–41, DFLARYRLVSNKLKKRFLRKP), while the corresponding stretch in CG8134 has seven strongly basic and nine hydrophobic amino acids (a.a. 38–61, NLIEQYLRASSKIKKFERAGFFKR). Such a unique and conserved amino acid composition implies a potential functional importance for this N-terminus motif. Similarly, the second predicted α-helices (labeled as “CC” in Fig 3A–3C) is characterized by two highly conserved cysteine residues (a.a. 69 and 76 in human HAP40. Fig 3A). The following α-helices (labeled as “EARFL” in Fig 3A–3C) is marked by its highly conserved stretch of amino acids composed of glutamic acid (E), alanine (A), arginine (R), phenylalanine (F) and leucine (L) residues. The predicted ninth α-helices is marked by its long leucine repeat (“LR” in Fig 3A–3C, a.a. 260–280 in human HAP40), containing four continuous leucine residues in dHap40 and six leucine in human and mouse HAP40, followed by two or three continuous proline (“P” in Fig 3A–3C) before the next α-helices. Lastly, E331 at the C-terminus of human HAP40 exist across all the species (red arrowheads in Fig 3A), supporting its functional importance in mediating the direct interaction with the bridge domain of HTT as revealed in the Cryo-EM study [36].

When expressed in *E*. *coli*., *cg8134* indeed produced a protein product at the predicted ~40kDa size (Fig 3E). In addition, in whole animal homogenates, two independent antibodies (1049 (α -CG8134 #1) and 1050 (α -CG8134 #2), S3 Fig) that were raised against bacteria-purified full-length CG8134 both detected a ~40kDa band that was present in wildtype flies but completely absent in all the established *cg8134*-null mutant lines (Fig 3D and 3F). Moreover, in flies with ectopic overexpression of *cg8134* from a UAS-*cg8134* transgene, which served as a positive control for CG8134 expression, the levels of the same ~40kDa band became significantly higher (arrows in Fig 3F, detected by 1049 (α-CG8134 #1) antibody). Further, when human HAP40 protein was expressed in fly tissues from transgenic flies, it also ran at similar size as fly *cg8134* (Fig 3G). Lastly, both anti-CG8134 antibodies could co-precipitate endogenous dHtt protein from whole animal homogenates (Fig 3H), supporting their physical interaction *in vivo*. Taken together, these results demonstrated that the ~40kDa protein co-purified with dHtt protein is the protein product of *cg8134* gene. The overall sequence and structural similarities between CG8134 and vertebrate HAP40 suggest that *cg8134* encodes the fly ortholog of HAP40, a conclusion supported in subsequent functional studies (see below). We therefore renamed the *cg8134* gene as *dhap40* (*Drosophila* HAP40 (HTT-associated protein 40kDa).

### *dhap40* and *dhtt* share similar phenotypes in *Drosophila*

**Fig 4:**
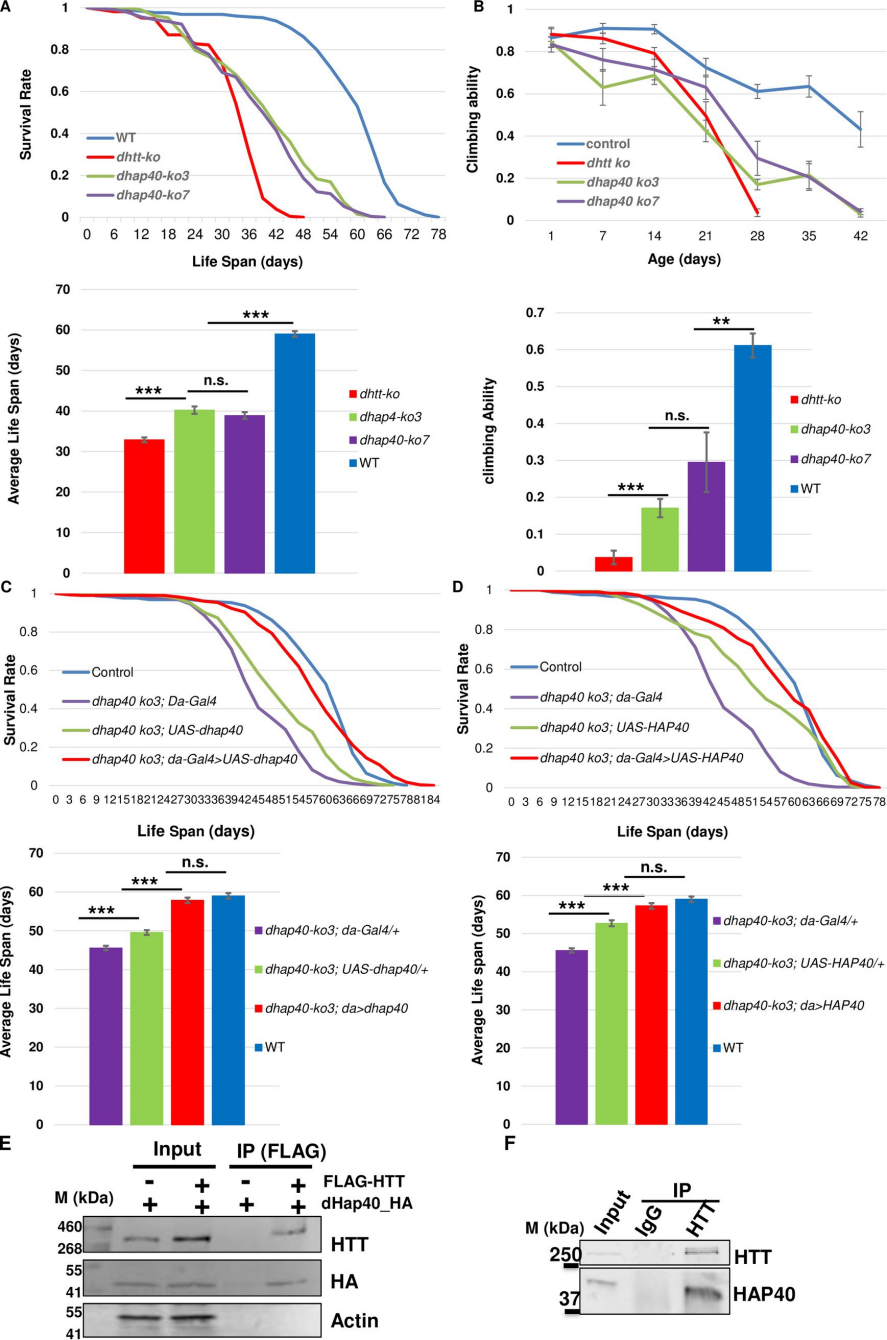
*dhap40* mutants show similar loss-of-functions phenotypes as *dhtt* null flies. (A) Survival curves (top) and bar-chart panels on average life span (bottom) as well as (B) climbing assays (top) and bar-chart panels on climbing ability (bottom) for two *dhap40* null alleles *ko3* and *ko7* (green and purple lines, respectively), together with wildtype control (blue line) and null *dhtt-ko* mutants (red line), as indicated. Note that both *dhap40* mutant alleles manifested similar, albeit significant weaker, phenotypes than *dhtt-ko* flies. (A) *dhap40-ko* had an average life span of 40 days (n = 230 total), differing significantly (p<0.001, log-rank test) from both wildtype (59 days, n = 253) and null *dhtt-ko* mutants (33 days, n = 209). (B) Climbing assays for 28-day-old adult flies of the indicated genotypes, with *dhap40* flies (n = 180) showing significant differences (** p<0.01 and *** p<0.001, *t*-test) from both wildtype (n = 200) and *dhtt-ko* mutants (n = 180). (C and D) Survival curves (top) and bar-chart panels on average life span (bottom) for rescue experiments on the longevity deficit of *dhap40*^*ko3*^ flies, restored by ectopically expressed (C) fly dHap40 from the UAS-dhap40 or (D) human HAP40 from UAS-HAP40 transgenes, all directed by a ubiquitous *da*-Gal4 driver (red lines. Genotype: “*w*^*1118*^, *dhap40*^*ko3*^; UAS-dhap40/da-Gal4” (C, n = 300) or “*w*^*1118*^, *dhap40*^*ko3*^; UAS-HAP40/da-Gal4” (D, n = 307)). The survival curves of the rescued flies in (C) and (D) were statistically indistinguishable from wildtype (blue lines, p>0.5, log-rank test n = 253. Genotype: “*w*^*1118*^”), but significantly different from the two controls: (1) *dhap40*^*ko3*^ flies carrying da-Gal4 driver alone (purple lines. *** p<0.001, log-rank test. Genotypes: “*w*^*1118*^, *dhap40*^*ko3*^; da-Gal4/+”. n = 308); and (2) *dhap40*^*ko3*^ flies carrying (C) UAS-*dhap40* (Genotype: “*w*^*1118*^, *dhap40*^*ko3*^; UAS-dhap40/+”. n = 355) or (D) UAS-HAP40 (Genotype: “*w*^*1118*^, *dhap40*^*ko3*^; UAS-HAP40/+”. n = 337) transgene alone (green lines, *** p<0.001, log-rank test), as indicated. The same set of data from wildtype (blue lines) and “*dhap40*^*ko3*^; da-Gal4/+” (purple lines) were used in both (C) and (D) as shared controls. (E and F) co-IP experiments between (E) transfected HTT and dHap40 in HEK293 cells or between (F) endogenous HTT and HAP40, as indicated. Note that the pulldown efficiency by HTT was significantly higher against (F) endogenous HAP40 than (E) dHap40.

To investigate the physiology functions of *dhap40* in *Drosophila*, we generated UAS-based transgenic flies for *cg8134* full-length cDNA that allow its targeted overexpression in different fly tissues (Fig 3F) [41], and also created multiple independent *cg8134* mutant alleles using the CRISPR/Cas9 approach [42–45] (Figs 3D and S2B–S2F). The established *dhap40* mutant alleles harbor various molecular lesions, including deletions and frame shifts, in the coding region of *dhap40* gene (Figs 3D and S2B–S2F). Consistently, for each of the alleles, the endogenous dHap40 protein was absent in homogenates of homozygous mutant flies (Fig 3F), suggesting that they are all protein-null. Notably, all these established *dhap40* null alleles are homozygous viable and developed normally into adulthood. Further, both male and female mutant flies are fertile with no apparent morphological defects. These observations are reminiscent of *dhtt*-null flies, which are also homozygous viable and fertile with no apparent developmental defects [22]. However, the *dhtt*-null adults develop ageing-related phenotypes, including shortened lifespan, reduced mobility and mild autophagy defects [22,26,27]. Remarkably, *dhap40-ko* mutants manifested very similar phenotypes as *dhtt* null. All the *dhap40-ko* alleles examined died early, with an average life span of 40 days, compared to 33 days for *dhtt*-null flies and 59 days for WT flies (Fig 4A). As the flies become older, the *dhap40-ko* flies also showed accelerated reduction of their mobility, as measured by climbing assay, mirroring that of *dhtt*-null flies (Fig 4B). Importantly, all observed phenotypes could be rescued by ectopically expressed *dhap40* from the UAS-*dhap40* (*cg8134*) transgenes driven by a ubiquitously *daughterless*-Gal4 (da-Gal4), thus confirming that the observed defects of *dhap40-ko* animals are due to the loss of endogenous dHap40 (Fig 4C). The similarity in loss of function phenotypes between *dhtt* and *dhap40* flies suggest that the two genes share similar physiological roles and likely function together in the same or overlapping cellular processes. However, it is noticeable that overall, the phenotypes of *dhap40* mutants were relatively weaker than that of *dhtt-ko* flies, living on average about 7 days longer (Fig 4A) and their mobility declining at a slower pace (Fig 4B), potentially indicating a modulatory role by HAP40 within the HTT/HAP40 complex in facilitating the cellular functions executed by HTT.

### HAP40 is functionally conserved

The above results prompted us to examine the potential functional conservation of HAP40 between the fly and humans. We first tested whether human HAP40 could rescue the loss of function phenotypes of *dhap40-ko* flies. Indeed, when directed by the same da-Gal4 driver, human HAP40 significantly suppressed all the observed mutant phenotypes of *dhap40-ko* flies, including the shortened lifespan and reduced mobility (Fig 4D). Such a functional rescue would predict that *Drosophila* dHtt still retains the ability to physically interact with human HAP40 protein, and dHap40 with HTT. Indeed, in HEK293 cells, HTT could co-immunoprecipitate (co-IP) ectopically expressed dHap40 protein from transfected cells (Fig 4E), although the pulldown efficiency was significantly lower than the robust pulldown of endogenous HAP40 by HTT in parallel co-IP experiments (Fig 4F), suggesting a potentially conserved albeit significantly reduce binding affinity between fly HAP40 and human HTT. Such a conserved physical interaction would be consistent with the observation that the N- and C-terminal regions that are important for HTT-binding are highly conserved between dHap40 and HAP40 (Figs 3A and S2A), which might be the underlying basis for the functional conservation of HAP40 between these two evolutionarily very distant species. Together, these results support that *Drosophila* CG8134/dHap40 is the functional ortholog of HAP40.

### High levels of HAP40 are not toxic in *Drosophila*

An early study reported an up to ~10-fold increase of the levels of HAP40 protein in samples from HD patients and mouse HD models, potentially implicating a toxic effect from elevated HAP40 expression [35]. We therefore examined the effects of HAP40 overexpression in various fly tissues. Using the established UAS-transgenic lines, driven by different Gal4 lines, we achieved dHap40 overexpression at levels ~10 folds or more than that of the endogenous dHap40 (Fig 3F). However, in all the Gal4 lines tested, including the ubiquitous (*i*.*e*., da-Gal4, Act-Gal4 and Tubulin-Ga4), neuronal-specific (*i*.*e*., Elav-Gal4 and Syt-Gal4) and eye-specific GMR-Gal4, dHap40 overexpression did not cause any apparent effect such as animal lethality and eye degeneration (S4 Fig). Similarly, no apparent detrimental effects were observed in flies overexpressing human HAP40 from the established transgenic lines (Fig 3G and examples in S4 Fig). Together, they suggest that high levels of HAP40 might not be toxic by itself, at least in the tested *Drosophila* tissues.

### HTT and HAP40 are mutually dependent for their protein stability

**Fig 5:**
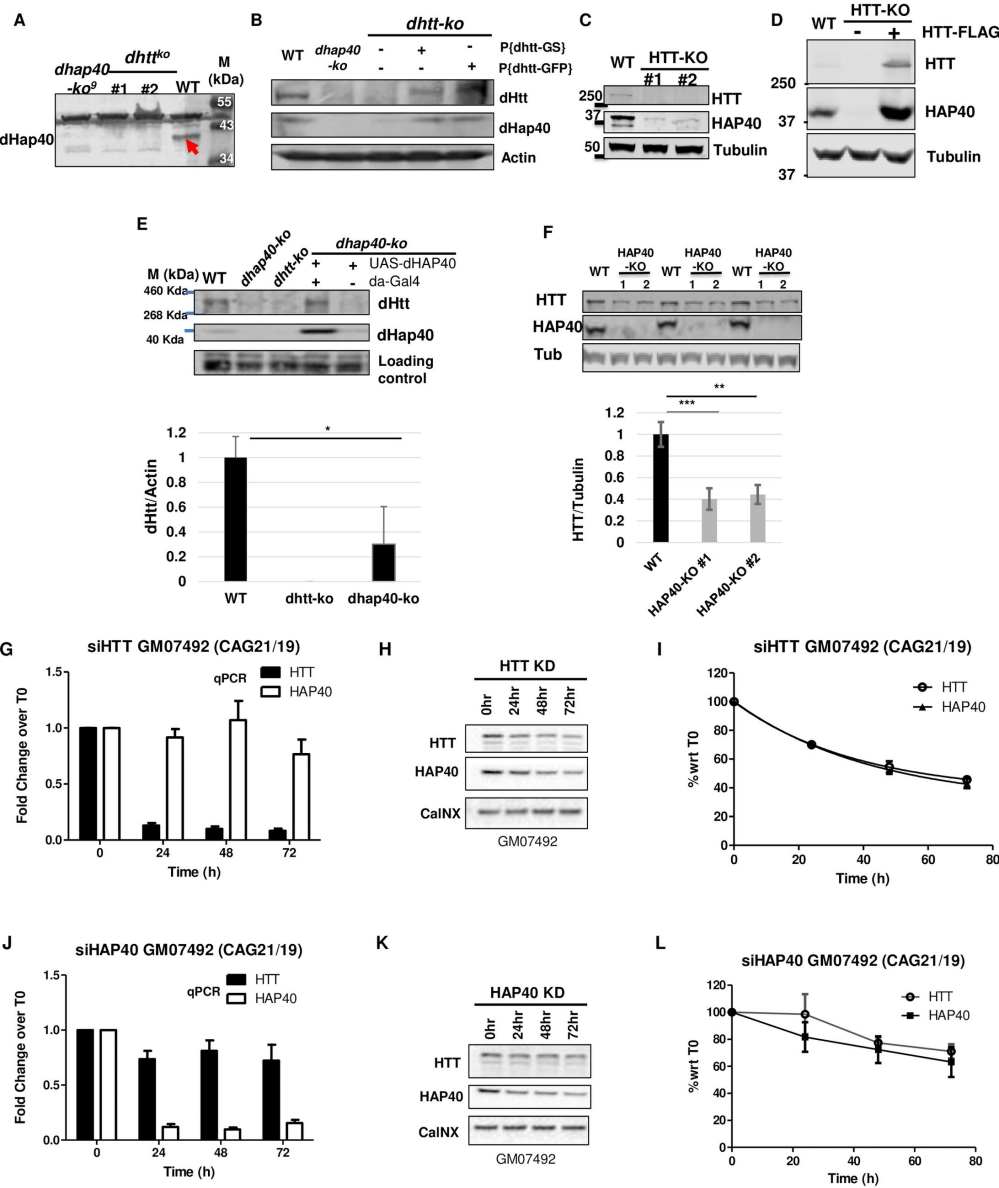
Conserved mutual dependence between HAP40 and HTT proteins on each other’s stability. Western blot assays of HTT and HAP40 proteins in flies and human cells. (A) The 40kDa endogenous dHap40 (arrow) protein in wildtype control (WT) was absent in two independent *dhtt* null alleles, similar as a *dhap40* null allele. Genotypes as indicated. A background band of 50kDa size present in all samples served as loading control. (B) Rescue experiment for *dhtt-ko* flies. Re-introduction of one copy of pacman-dhtt-GS (lane 4) or pacman-dhtt-eGFP (lane 5) transgenes into *dhtt-ko* flies rescued the expression of endogenous dHtt and dHap40 proteins. Genotypes as indicated. Actin served as loading control.(C) Endogenous HAP40 was significantly depleted in two independent HTT-knockout (HTT-KO) HEK293 cell lines, as compared to wildtype HEK293 control (WT). α-Tubulin serves as loading and normalization controls. (D) HAP40 was severely depleted in HTT-KO cells (lane 2) and was restored by overexpressed FLAG-HTT to a level even higher (lane 3) than in WT control (lane 1). α-Tubulin serves as loading and normalization controls. (E) Rescue experiment for *dhap40-ko* flies. Expression of dHtt and dHap40 proteins in WT, *dhap40* ^*ko3*^ and *dhtt* ^*ko*^ null adult flies, or *dhap40* ^*ko3*^ flies with UAS-dHap40 rescue transgene (lanes 4 and 5), as indicated. Bar chart quantification below shows an average of 70% reduction of endogenous dHtt protein in null *dhap40* ^*ko3*^ flies as compared to WT control (N = 4 four repeat experiments, p = 0.02, student’s *t*-test). dHtt expression was restored in *dhap40* ^*ko3*^ flies when dHap40 was overexpressed from UAS-dHap40 transgene driven by da-Gal4 driver (lane 4), but not in the control flies that lacked da-Gal4 driver (lane 5). A ~50kDa background band from anti-dHap40 antibody served as loading controls. (F) Reduced levels of endogenous HTT protein, shown in three independent Western blot assays, in two independent HAP40-KO cell lines and their quantification in the bar chart below, showing an average of ~60% reduction of normalized HTT levels as compared to WT control. α-Tubulin serves as loading and normalization controls (** p<0.01 and *** p<0.001, *t*-test). (G-L). Representative data on the turnover dynamics of (G-I) HAP40 or (J-L) HTT proteins after transient knockdown of endogenous (G-I) HTT or (J-L) HAP40, respectively, by siRNA in a normal human fibroblast cell line GM07492. All three independent repeats showed similar results. The knockdown efficiency and specificity of the siRNAs targeting HTT or HAP40 were evaluated (G and J) at mRNA levels by quantitative PCR (qPCR) and (I and L) at protein levels by Western blot assays. (I and L) Quantification of the relative levels of HTT and HAP40 proteins, all normalized again loading control Calcineurin (CalNX), from Western Blot assays (G and I) at indicated time points after siRNA treatment. The data were plotted with Time 0 as reference point (N = 3 independent repeats). GM07492 fibroblast cell line contains two normal HTT alleles, one with 19 and the other 21 CAG repeats.

Given the highly conserved physical and functional interaction between HTT and HAP40, we next tested how they might regulate each other. Strikingly, in *Drosophila*, in two independent null *dhtt* alleles, loss of dHtt led to an almost complete depletion of endogenous dHap40 protein (Fig 5A), a phenotype that was rescued by re-introducing into the *dhtt-ko* flies a single copy of the dhtt-eGFP or dhtt-GS pacman transgenes (Fig 5B), demonstrating the causative link between the simultaneous loss of endogenous dHtt and dHap40 proteins. To test whether such regulation is also conserved in mammalian cells, we next created multiple independent HTT knockout (HTT-KO) lines from HEK293 cells using the CRISPR/Cas9 method. Indeed, in these HTT-KO cells, which were validated for the absence of the endogenous HTT by Western blot assays, the levels of endogenous HAP40 were dramatically reduced as compared to that in control cells (Fig 5C). Importantly, restoring HTT expression in the HTT-KO cells, by transfecting plasmids encoding full-length HTT, led to a full rescue of the levels of endogenous HAP40 protein. Moreover, when HTT was overexpressed above its endogenous levels, it resulted in accumulation of HAP40 above its endogenous levels as well (compare lane 3 with lane 1 in Fig 5D).

Given the above findings, we next examined whether HTT levels are similarly affected by the loss of HAP40. In *Drosophila*, in all the tested *dhap40-ko* alleles the levels of endogenous dHtt were ~70% lower than that in wildtype controls (Fig 5E), a phenotype that could be rescued by re-introducing the expression of dHap40 back in *dhap40-ko* flies using UAS-dHap40 transgenes (lane 4 in Fig 5E). Similarly, in independent HAP40 knockout (HAP40-KO) HeLa cells that we created using CRISPR, loss of HAP40 led to a notable ~60% reduction of endogenous HTT protein (Fig 5F).

Since all the above results were from permanent knockout fly lines or cultured human cell lines, to explore the dynamics of this mutual dependence between HTT and HAP40 proteins, we next tested the effect of transient knockdown of HTT or HAP40 in human primary fibroblast cells by siRNA. siRNAs targeting HTT was highly specific and effective, resulting in more than 90% depletion of HTT mRNAs in the treated cells 24 hours after transfection, followed by a gradual reduction of HTT protein levels over the next 72 hours, while showing relatively no clear effect on HAP40 mRNA levels (Fig 5G and 5H). Importantly, in siHTT-expressing cells, HAP40 protein showed an almost identical curve in its rate of reduction as that of HTT (Fig 5I), suggesting a rapid degradation of HAP40 concurrent with HTT clearance. Similarly, in siHAP40-treated cells, which also caused a ~90% depletion of endogenous HAP40 mRNA levels but no effect on HTT mRNA (Fig 5J), the levels of HTT protein also turned lower following a similar trend as that of endogenous HAP40 (Fig 5K and 5L), supporting that HTT protein became destabilized in the absence of endogenous HAP40. Together, these results demonstrated that HTT and HAP40 proteins are mutually dependent on each other for their own stability, a regulation that is highly conserved during evolution from flies to mammalian cells.

### HAP40 is degraded through proteasome

**Fig 6:**
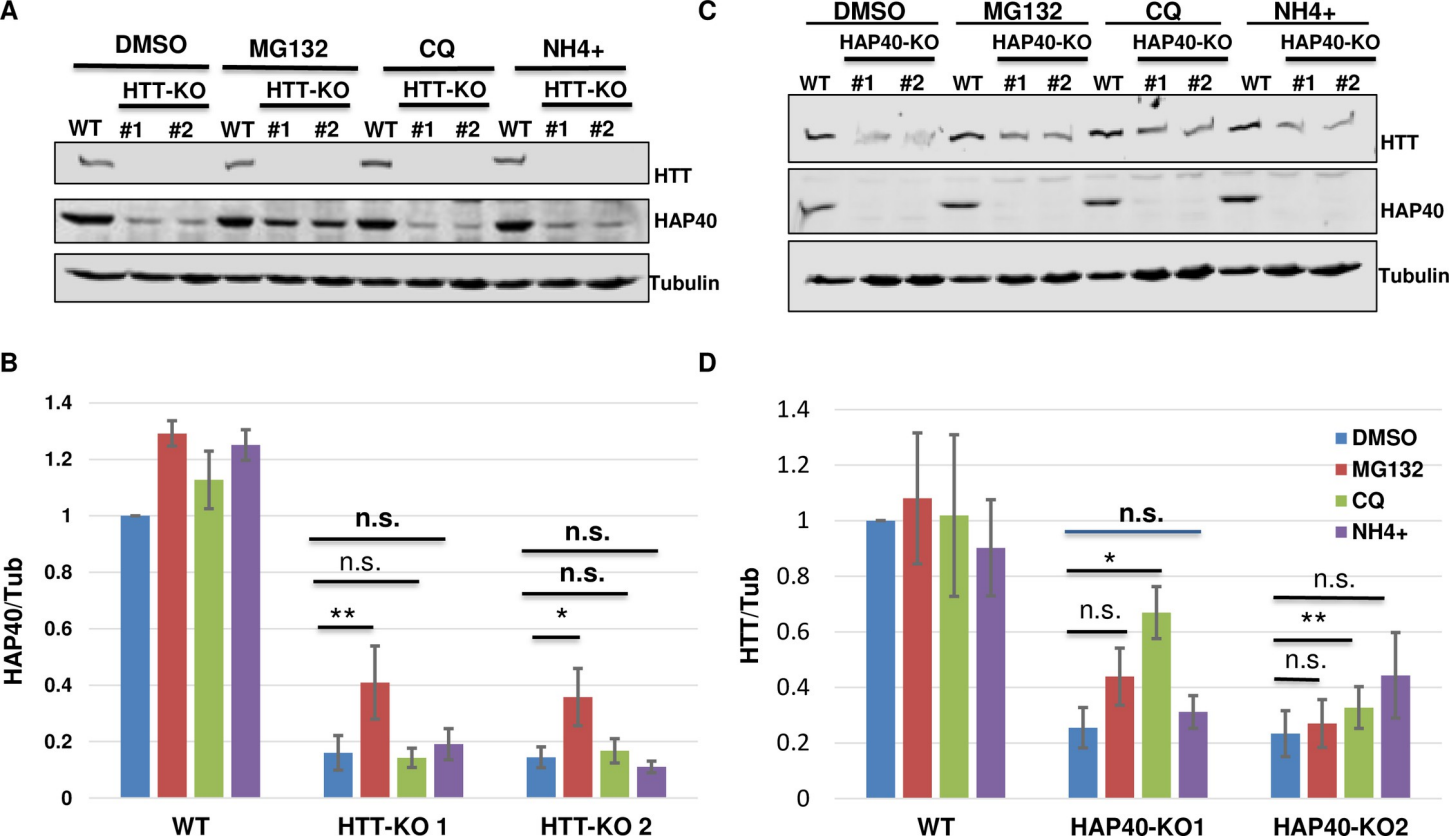
Proteasome mediates HAP40 degradation. Western blot assays and quantifications for endogenous HAP40 and HTT proteins in HTT-KO, HAP40-KO or wildtype (WT) HEK293 cells under different treatments, as indicated. (B and D) Normalized levels of (B) HAP40 or (D) HTT proteins from three repeat experiments, corresponding to (A) and (C), respectively. Treatment with proteasome inhibitor MG132 for 5 hours partially but significantly restored the levels of depleted HAP40 protein in HTT-KO cells (A and B, N = 5 repeats for all the experiments, p = 0.002 for HTT-KO #1 line, p = 0.018 for HTT-KO #2 line), but showed no clear effect on the levels of endogenous HTT protein in HAP40-KO cells (C and D, N = 4 repeats for all the experiments). Autophagy/lysosome inhibitor ammonium (NH4+) and CQ, also treated for 5 hours. CQ showed partially but significant rescue of HTT levels in HAP40-KO cells (N = 4 repeats for all the experiments. p = 0.016 for HAP40-KO #1 line, p = 0.002 for HAP40-KO #2 line). NH4+ behaved similarly as DMSO mock treatment in both HTT-KO and HAP40-KO cells. * p< 0.05, ** p< 0.01 (student’s t-test). n.s., no significance. α-Tubulin served as loading and normalization controls in all the experiments.

We further explored the mechanism underlying the mutual dependence between HTT and HAP40 proteins. In HTT-KO cells, application of proteasome inhibitor MG132 partially restored the levels of endogenous HAP40. In contrast, autophagy and lysosome inhibitors chloroquine (CQ) or ammonium had no discernable effect on the levels of endogenous HAP40 (Fig 6A and 6B), suggesting that in the absence of HTT, HAP40 is quickly cleared by the proteasome system. However, when these inhibitors were similarly applied to HAP40-KO cells, CQ showed a variable but statistically significant capacity in partially restoring the levels of endogenous HTT in two independent HAP40-KO lines, but neither ammonium nor MG132 could significantly rescue the diminished levels of HTT protein within the duration of the drug treatment (Fig 6C and 6D). Treatment with autophagy inhibitors 3-MA and bafilomycin also could not meaningfully restore HTT levels in HAP40-KO cells (S5 Fig). Such results raised a question on whether autolysosomal pathway plays a role in HTT degradation. Nevertheless, these results suggest potentially different protein dynamics between HTT and HAP40. The smaller HAP40 protein might be a preferential target of the proteasome in the absence of HTT, while the much larger HTT might be relatively more stable and refractory to degradation in the absence of HAP40.

### HAP40 reduces the turnover rate of endogenous HTT

**Fig 7:**
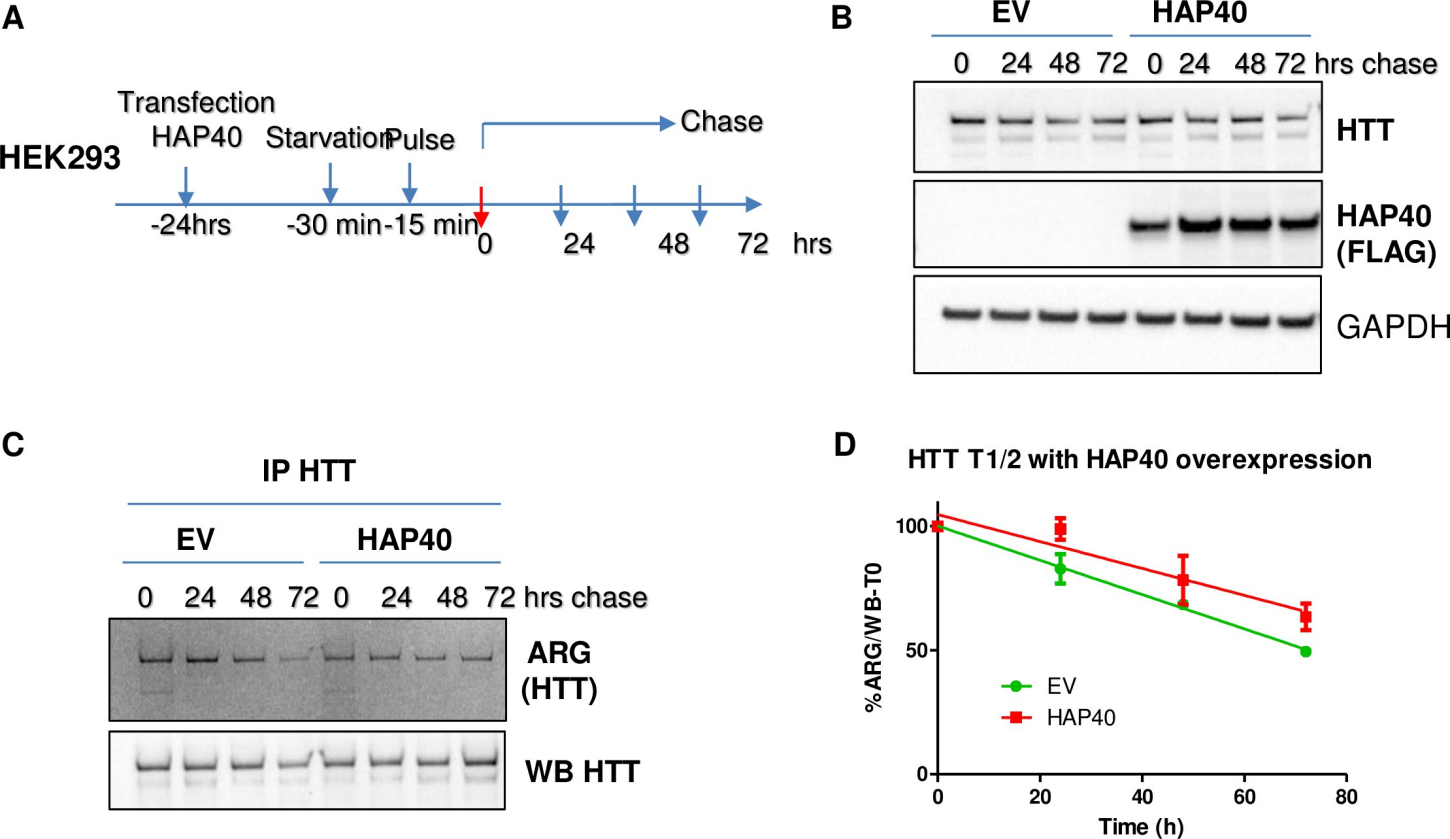
HAP40 overexpression extends the half-life of endogenous HTT proteins. Pulse-chase experiments to measure the turnover rates of endogenous HTT proteins in HEK293 cells. (A) Schematics of pulse-chase experiments. HEK293 cells were cultured in regular medium for 24 hours after transfection with control empty vector (EV) or construct expressing FLAG-tagged HAP40, followed by standard pulse-chase experiment. After starvation in methionine-free medium for 30 minutes, cells were supplemented with medium containing ^35^S-methionine for another 15 minutes to label the newly synthesized proteins. After the pulse labeling, the cells were washed and then maintained in regular medium for indicated intervals before harvesting for further analysis. (B) Western blot assay to measure the total levels of endogenous HTT and transfected FALG-tagged HAP40 proteins at each indicated time points in control and HAP40-overpresssing cells. GAPDH as loading control. (C) Endogenous HTT protein were enriched by immunoprecipitation with D7F7 anti-HTT antibody and resolved by SDS-PAGE separation, followed by autoradiography (ARG) and Western blot assays to measure the amount of ^35^S-labeled and total HTT protein at each time point, as indicated. (D) Turnover rate of endogenous HTT protein in control and HAP40-overexpressing cells, which was quantified as the relative levels of remaining ^35^S-labeled HTT at each time point after the start of the chase, all normalized against total HTT from each pulldown. Half-life for endogenous HTT at ~73 ± 4hrs hours in EV control and ~103 ± 18 hours in the presence of overexpressed HAP40 (N = 3; p<0.05 (student’s *t*-test)).

To further understand how HAP40 might affect HTT protein, we carried out pulse-chase experiment in HEK293 cells to measure the turnover rate of endogenous HTT, which revealed that the half-life of endogenous HTT was approximately 65 hours (S6 Fig) in wildtype HEK293 cells. Significantly reduced levels of endogenous HTT in HAP40-KO cells (Fig 5F) made it not feasible to reliably quantify the turnover rate of HTT protein in HAP40-null cells using the similar pulse-chase experiment. We then examined the effect of higher levels of HAP40 on HTT stability (Fig 7). Remarkably, HAP40 overexpression significantly reduced the turnover rate of endogenous HTT and extended its half-life from the 65 hours measured in control (green line in Fig 7D) to over 100 hours by extrapolation with the presence of HAP40 overexpression (red line in Fig 7D), further supporting that HAP40 is an effective regulator of HTT stability.

### PolyQ expansion in HTT has minimal effect on its binding affinity for HAP40

**Fig 8:**
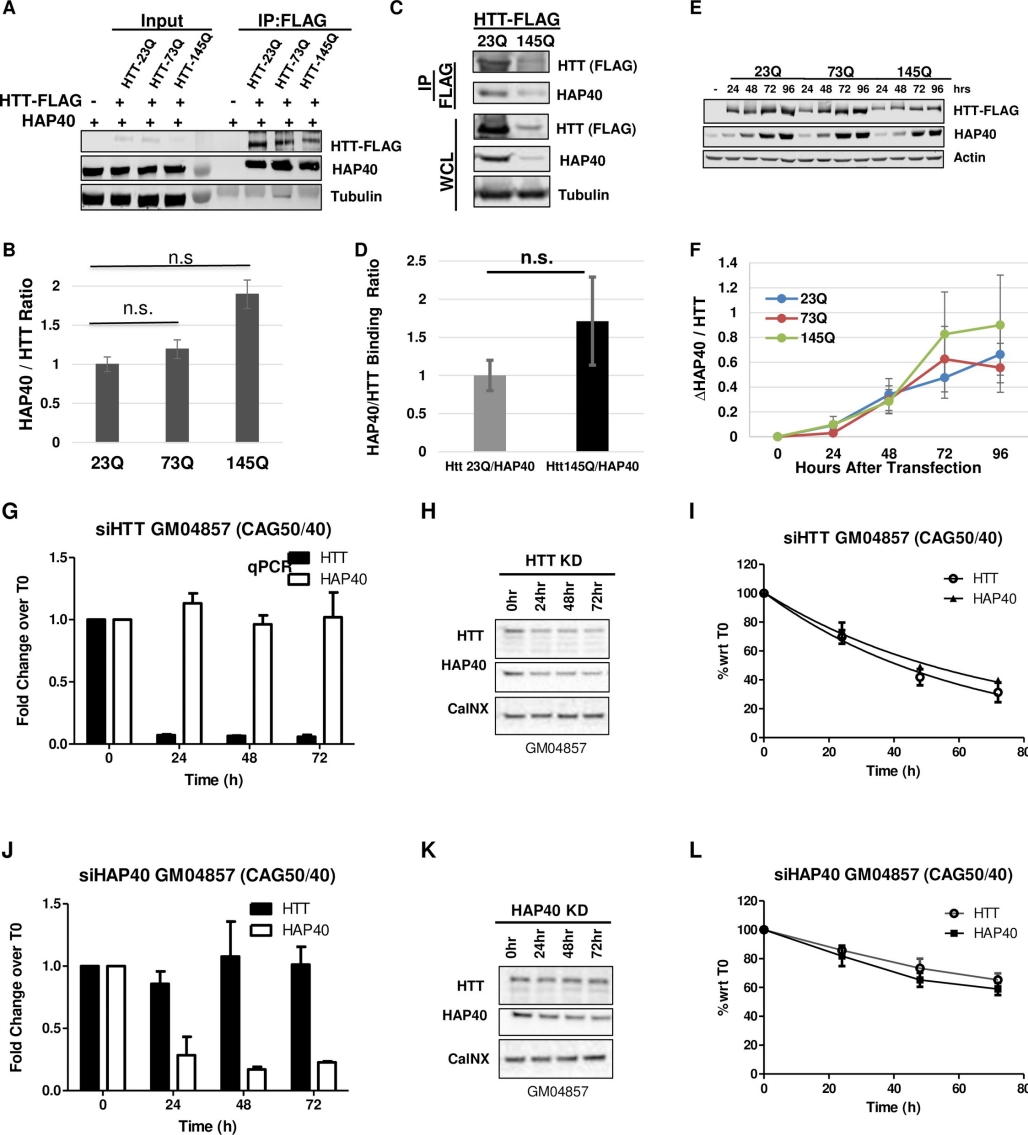
PolyQ expansion in HTT does not significantly affect its affinity for HAP40. (A and C) Western blot assay and (B and D) quantification of co-IP experiments between HAP40 and FLAG-tagged full-length HTT carrying 23, 73 or 145 (23Q, 73Q and 145Q) glutamine tracts expressed either from transient transfection (A and B) or stable cell lines expressing HTT-Q23 or HTT-Q145 (C and D), as indicated. (B and D) The pull-down efficiency of HAP40 by different HTT proteins were quantified as the relative ratio of co-immunoprecipitated HAP40 and HTT proteins, averaged from three independent experiments. All three HTT proteins showed comparable pulldown efficiency for endogenous HAP40. n.s., no significance. α-Tubulin serves as loading control. (E and F) Time course analyses of endogenous HAP40 and ectopically expressed HTT-23Q, 72Q or 145Q in HTT-KO HEK293 cells, hours after transfection with FLAG-tagged HTT expressing plasmids, as indicated. (F) Quantification of the time-dependent changes of HAP40 levels normalized against HTT at each time point, averaged from three repeat experiments. (G-L). Representative data on the turnover dynamics of endogenous (G-I) HAP40 or (J-L) mutant HTT proteins after transient knockdown of (G-I) HTT or (J-L) HAP40, respectively, by siRNA in a human HD fibroblast cell line GM04857. All three independent repeats showed similar results. The knockdown efficiency and specificity of the siRNAs targeting HTT or HAP40 were evaluated (G and J) at mRNA levels by quantitative PCR (qPCR) and (H and K) at protein levels by Western blot assays. (I and L) Quantification of the relative levels of HTT and HAP40 proteins, all normalized again loading control Calcineurin (CalNX), from Western Blot assays (representative images in H and K), respectively, at indicated time points after siRNA treatment, plotted with Time 0 as reference point (N = 3 independent repeats). GM04857 is a fibroblast cell line derived from a homozygote HD patient with pathogenic CAG expansion in both HTT alleles, one with 40 CAG repeats and the other 50 CAG repeats.

As polyQ length correlates with mutant HTT toxicity, we next examined whether polyQ expansion in HTT could affect its affinity for HAP40. We first performed co-IP assays between HAP40 and FLAG-tagged full-length HTT proteins carrying a polyQ tract with 23, 73 or 145 glutamines. In transfected HEK293T cells, wildtype and mutant HTT proteins showed comparable pull-down efficiency against endogenous HAP40, quantified based on the ratios of co-IPed HAP40 and HTT proteins in repeat experiments (Fig 8A and 8B). Interestingly, albeit not statistically significant, the quantification result implied a trend of slightly higher affinity between HAP40 and mutant HTT-145Q, whereas the binding affinity of HAP40 with HTT-23Q and HTT-73Q were virtually the same (Fig 8B). To exclude the possibility that the observation was influenced by transient expression of mutant HTT, we next established stable cell lines expressing FLAG-tagged wildtype (23Q) or mutant (145Q) HTT proteins in HEK293T cells. Co-IP experiments using these stable HTT-expressing cell lines again showed a comparable binding affinity of HAP40 with wildtype or mutant HTT (Fig 8C and 8D). To further test the above observation, we took advantage of the findings that the stability of endogenous HAP40 relies almost exclusively on the presence of HTT (Fig 5), and compared the efficiency of wildtype and mutant HTT in restoring the levels of endogenous HAP40 in HTT-KO HEK293 cells. Quantification data from this rescue experiment showed that in HTT-KO cells, both mutant HTT-73Q and HTT-145Q restored the levels of endogenous HAP40 protein as effectively as wildtype HTT-23Q (Fig 8E and 8F). Nevertheless, time course analyses, by comparing the relative levels of HAP40 protein versus that of the ectopically expressed HTT at different time points after HTT transfection in HTT-KO cells, again suggested a trend of slightly better rescue efficiency by HTT-145Q than HTT-73Q and HTT-23Q in restoring the expression of endogenous HAP40 (Fig 8F), although the difference was too weak to reach a statistical significance in this HAP40 rescue experiment. Lastly, as ectopic expression of HTT in wildtype cells can induce higher levels of endogenous HAP40 (Fig 5D), we further compared wildtype or mutant HTT for their overexpressing effect on the levels of endogenous HAP40 protein in normal HEK293T cells. The results were similar as that in HTT-KO cells, with HTT-145Q being slightly more effective than HTT-23Q and HTT-73Q in inducing higher levels of endogenous HAP40 (S7 Fig).

The above result would suggest that the mutual dependence between HTT and HAP40 proteins is not affected by polyQ expansion in HTT. To test this prediction in a pathological setting, we knocked down HTT or HAP40 expression in GM04857, a human HD homozygote fibroblast cell line that was derived from a HD patient homozygous for HD mutations, with one of the HTT alleles containing 40 CAG repeat and the other 50 CAG repeat. Similar as that observed in normal human fibroblasts (Fig 5H–5L), depletion of mutant HTT expression in HD cells by siHTT led to a parallel reduction of endogenous HAP40 protein (Fig 8G–8I), while conversely HAP40 knockdown by siHAP40 over a 72 hour period resulted in a parallel depletion of mutant HTT protein as well (Fig 8J–8L). Taken together, these data support that polyQ expansion in HTT does not compromise its affinity for HAP40, nor does it influence the mutual dependence between HAP40 and mutant HTT proteins.

### The levels of HAP40 protein are not elevated in HD cells

**Fig 9:**
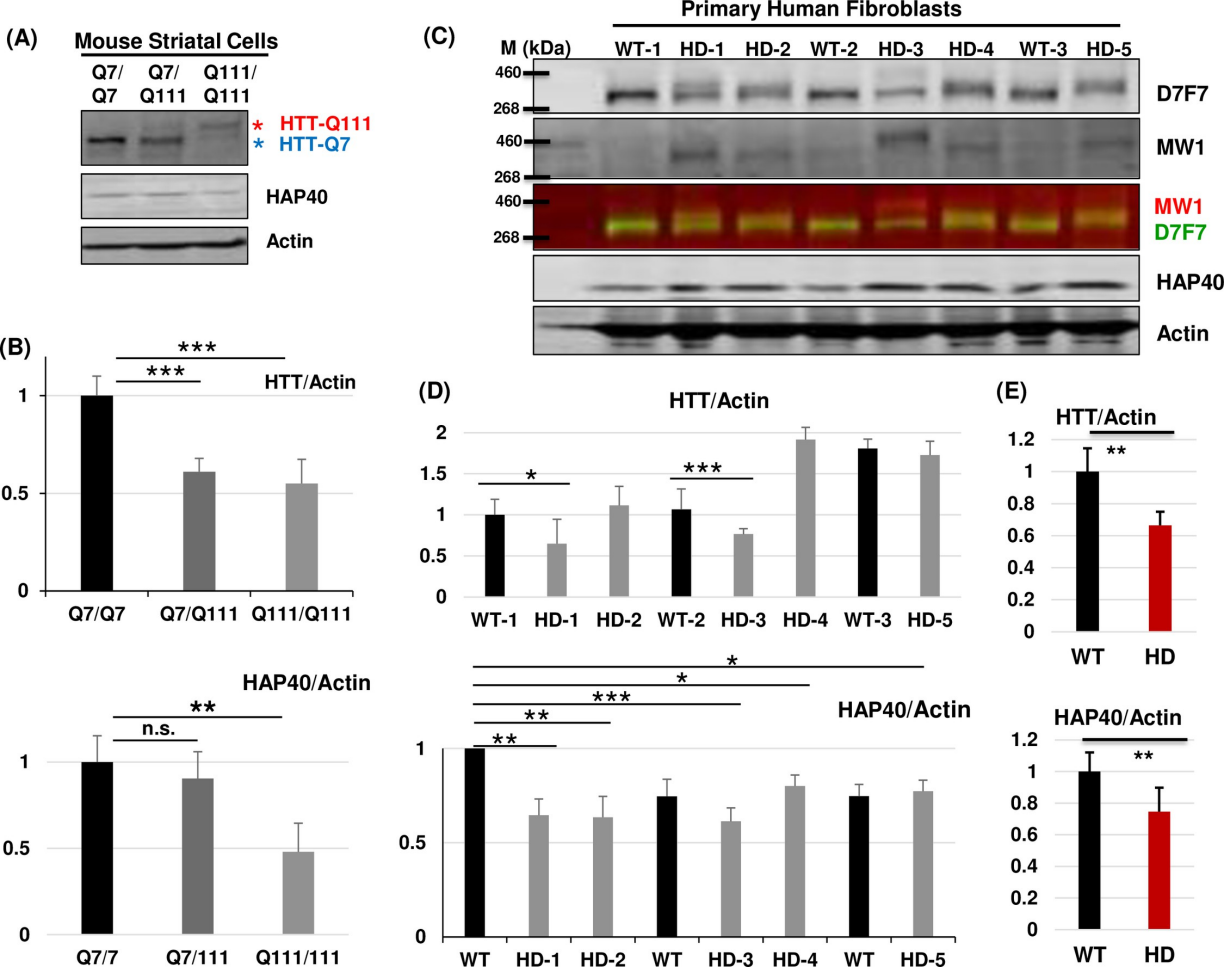
The levels of HAP40 protein are lower in HD cells. Western blot assays and quantification of endogenous HTT and HAP40 proteins in normal and HD (A and B) mouse striatal precursor cells and (C-E) human fibroblast cells, as indicated. The human fibroblast cell lines are: GM04729 (WT-1), GM05539 (HD-1), GM21757 (HD-2. CAG repeats reported as 66 and 16), GM02189 (WT-2), GM09197 (HD-3. 180 CAG repeat in affected HD allele), GM04723 (HD-4), GM04787 (WT-3) and GM21756 (HD-5. CAG repeats reported as 70 and 15). (A) Note that for each mutant HD cell lines in these SDS-PAGE gels, mutant HTT proteins (HTT-Q111, indicated by red *) were reduced and migrated slower than wildtype HTT (wtHTT or HTT-Q7, indicated by blue *) in a polyQ length-dependent manner. (C) The membrane was co-probed with both anti-total HTT (D7F7,) and mutant HTT specific (MW1, red) antibodies. In both (B) and (D), N = 6 for quantification of HAP40 protein levels and N = 4 for quantification of HTT. (E) Average relative ratio of the normalized HTT and HAP40 protein levels in wildtype (WT) and HD cells, quantified from the eight wildtype and 13 mutant HD fibroblast cells lines tested in repeats experiments (see S8 Fig for additional data). *p< = 0.05, **p< = 0.01, ***p< = 0.001 (student’s *t*-test). n.s., no significance. β-Actin served as loading and normalization controls in all the experiments.

Given the above findings, especially that HAP40 relies on HTT for its protein stability and that it binds wildtype and mutant HTT with similar affinity, and also considering that mutant HTT protein exists at similar or even lower levels as that of normal HTT in tissues from HD patients and multiple mouse models of HD [46, 47], one can speculate that the levels of HAP40 protein should not change significantly by HD mutations, a conclusion that would contradict with the early report of elevated HAP40 levels in HD cells and tissues [35]. To test this hypothesis, we first examined mouse striatal precursor cell lines STHdh Q7/Q7, STHdh Q111/Q111 and STHdh Q7/Q111, which were derived from knock-in transgenic mice homozygous (Q7/Q7 and Q111/Q111) or heterozygous (Q7/Q111) for mouse HTT (Hdh) loci with a humanized exon 1 containing normal 7 or expanded 111 polyglutamine repeats, respectively [48]. Noticeably, in heterozygous STHdh Q7/Q111 cells, the levels of mutant HTT-Q111 protein from the CAG-expanded Hdh allele were markedly lower than that of HTT-Q7 from the normal Hdh allele in the same cells (lane 2 in Fig 9A), suggesting that mutant HTT is significantly less stable. Consistently, the levels of HTT-Q111 protein in homozygous STHdh-Q111/Q111 cells were about ~50% lower than that of HTT-Q7 in homozygous STHdh-Q7/Q7 cells (compare lane 3 with lane 1 in Fig 9A). In line with the prediction, the levels of HAP40 proteins were not increased, but mildly decreased in heterozygous STHdh Q7/Q111 cells and became significantly lower in homozygous STHdh Q111/Q111 cells, at about 50% level of that in STHdh-Q7/Q7 cells (Fig 9B).

To further confirm this result in human setting, we next examined the levels of HTT and HAP40 proteins in human fibroblast cell lines derived from thirteen HD patients and eight normal controls. For the mutated HTT alleles among the thirteen HD lines, the exact lengths of CAG repeats in most lines were not known, in line GM21757 (HD-2) were reported as 66 and in line GM21756 (HD-5) as 70 (https://www.coriell.org/), while in line GM09197 (HD-3), which was derived from a Juvenile onset HD patient, it was unusually long at 180 CAG repeats [49]. Notably, mutant HTT (i.e., the upper HTT bands) in HD-3 migrated significantly slower than mutant HTT in other HD lines, likely due to its unusually long 180 CAG repeat. Similar to observations in mouse striatal cells, there was a reduction in full-length HTT protein (Figs 9C and S8). Importantly, MW1, which is specific for polyQ-expanded mutant HTT protein [50], confirmed that these bands correspond to mutant HTT protein (Fig 9C, comparing the total HTT proteins detected by D7F7 anti-HTT antibody (panel 1) with mutant HTT detected by MW1 (panel 2) and their overlaying signals (green for total HTT and red for MW1 in panel 3)). Further, although with significant variations, none of the tested HD cells showed elevated levels of HAP40 (Fig 9D). Line HD-3 (GM09197), apparently carrying the longest CAG repeats among the five tested HD lines, also showed an overall significantly reduced levels of total HTT protein with concurrent lower levels of HAP40. Quantification of all of the tested eight normal and 13 HD cell lines revealed overall a ~30% reduction of total HTT levels and ~20% reduction of HAP40 levels in HD cells as compared to that in normal cells (Fig 9E).

### HAP40-free mutant HTT is toxic

Considering the close physical and functional relationship between HAP40 and HTT, one important question is whether and how HAP40 might modulate the toxicity of mutant HTT. Multiple *Drosophila* models of HD have been created that can faithfully recapitulate the polyQ length-dependent toxicity of mutant HTT proteins [21], thus allowing us to address this question in this model organism. We first tested flies with neuronal expression of human HTT exon1 carrying 93 glutamines (HTTex1-93Q), a well-established HD model that manifests robust neuronal degeneration in targeted neurons [51]. Interestingly, loss of endogenous *dhap40* did not modify neuronal degeneration of HTTex1-93Q flies; there was no difference in internal photoreceptor cell loss (S9A Fig).

**Fig 10:**
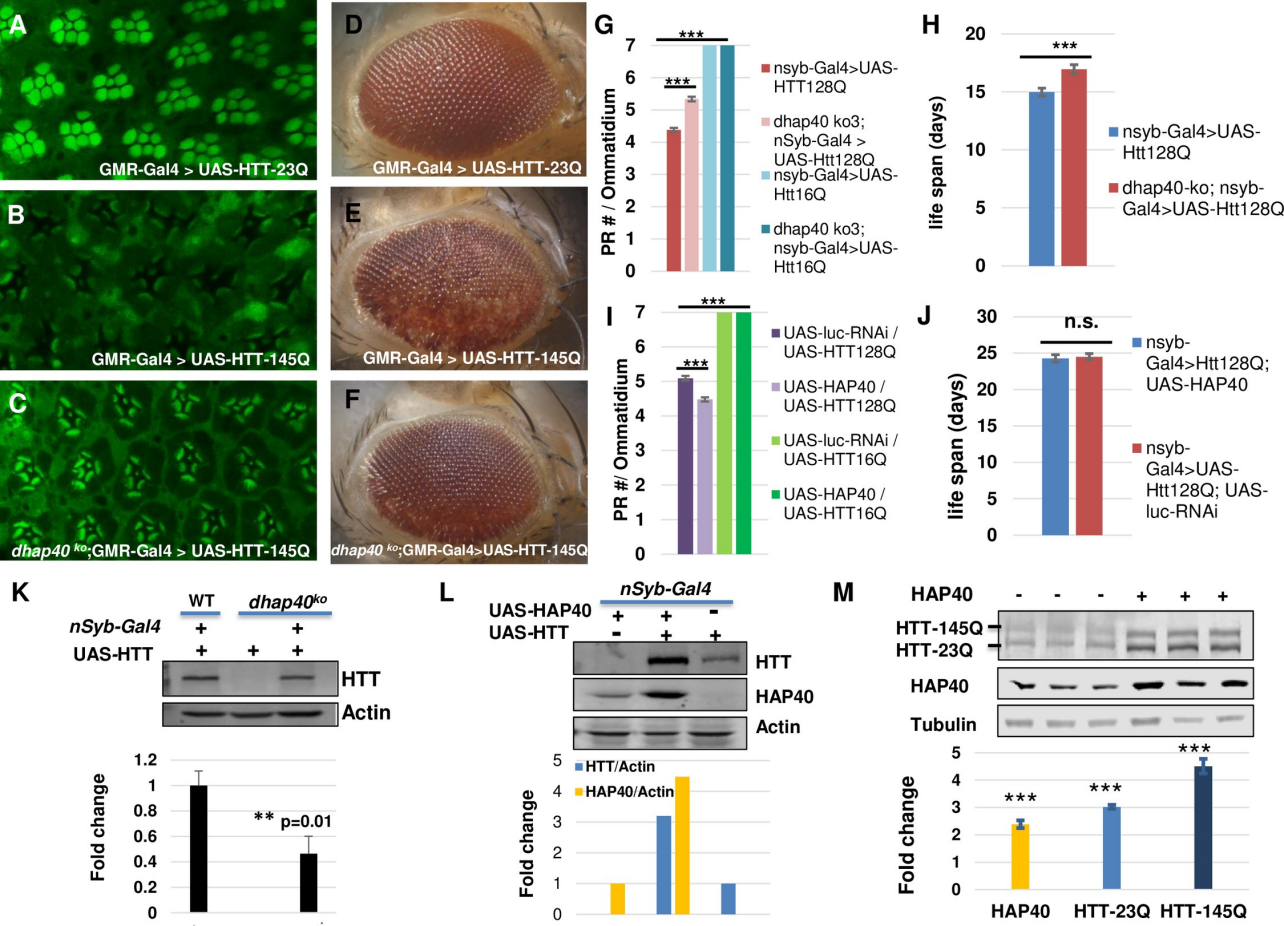
Mild effect of HAP40 on neurodegeneration of *Drosophila* HD models. (A-F) Representative (A-C) confocal images of whole-mount retina with phalloidin staining, dissected from 1-day-old adult female flies, or (D-F) bright-field image of 30-day-old adult fly eyes with eye-specific expression of (A and D) control wildtype fl-HTT-23Q, or (B, C, E and F) human mutant fl-HTT-145Q in (B and E) normal or (C and F) *dhap40*^ko3^ background, all directed by eye-specific GMR-Gal4 driver. Compared with (A) the control of the eye expressing wildtype HTT-23Q, which showed regular composition and patterning of the seven photoreceptors within each ommatidium unit, (B) mutant HTT-145Q already caused severe degeneration phenotypes, showing prominent loss of photoreceptor cells and disintegration of ommatidia structure, while (C) in the absence of endogenous *dhap40*, the eye degeneration phenotypes were significantly suppressed. Similarly, the loss of eye pigmentation induced by fl-HTT-145Q (E A), indicating underlying eye degeneration, was suppressed by the absence of *dhap40* (F). Genotypes: (A and D) GMR-Gal4/+ >UAS-hHTT-23Q/+. (B and E) GMR-Gal4/+ >UAS-hHTT-145Q/+. (C and F) *dhap40*^ko3^/*dhap40*^ko3^; GMR-Gal4/+ >UAS-hHTT = 145Q/+. Flies with the same genotype showed relatively similar phenotypes at the same age (examples in S9B–S9D Fig). More than 4 fly eyes were examined and imaged in (A-C) and 30 flies were examined in (D-F) for each of the genotypes. (G-J) Quantification of photoreceptor cell degeneration and viability phenotypes of flies with neuronal-specific expression of fl-HTT-128Q either (G and H) in *dhap40* null background or (I and J) with co-expression of human HAP40, all directed by pan-neuronal *nsyb*-Gal4 and with respective controls, as indicated. (G and I) Bar chart presentation of the average number of intact photoreceptor cells (PRC) per ommatidium in 7-day-old flies of the following designated genotypes. (G)*“nsyb-*Gal4/UAS-fl-HTT-128Q”: 4.4 PRC/ommatidium (n = 360 ommatidia from 20 flies); “*dhap40* ^ko3^*; nsyb-*Gal4/UAS-fl-HTT-128Q”: 5.3 PRC/ommatidium (n = 360 ommatidia from 20 flies); The difference between the two was significance (p<0.001, Mann-Whitney rank-sum test). Control flies expressing Fl-HTT-16Q in both normal and *dhap40*-ko backgrounds showed normal seven photoreceptor cells (blue bars). (I) “UAS-HAP40; *nsyb-*Gal4 *>*UAS-fl-HTT-128Q” flies: 5.1 PRC/ommatidium (n = 331 ommatidia from 20 flies); control “UAS-Luciferase-dsRNA; *nsyb-*Gal4 *>*UAS-fl-HTT-128Q” flies: 4.5 PRC/ommatidium (n = 320 ommatidia from 20 flies). The difference between the two is significance (p<0.001 by Mann-Whitney rank-sum test). Control flies co-expressing HTT-16Q with HAP40 or with luciferase (green bars) all had seven intact photoreceptor cells. (H and J) Bar chart presentation of the average life spans of the adult flies expressing fl-HTT-128Q. (H) Average life span was 17 days for “*dhap40* ^*ko3*^*; nsyb-*Gal4/UAS-fl-HTT-128Q” flies (n = 179) and 15 days for control *“nsyb-*Gal4/UAS-fl-HTT-128Q” flies (n = 229). The difference between the two is significance (p<0.001. Log-rank test). (J) Average life span was 24.3 days for flies co-expressing HTT-Q128 with HAP40 (genotype: “*nsyb*-Gal4>UAS-HAP40/+; UAS-HTT-128Q/+”. n = 141), and 24.5 days for control flies co-expressing HTT-Q128 with luciferase-dsRNA (“*nsyb*-Gal4> UAS-luciferase-RNAi/+; UAS-HTT-128Q/+”, n = 174), with no significant difference between the two genotypes (p = 0.9 by Log-rank test). (K-M) Western blot assays for the ectopically expressed human HTT protein in (K and L) flies and (M) human cells, and quantifications from three independent repeat experiments show in bar charts below. (K) The levels of human HTT expressed from the same UAS-HTT transgene was ~60% lower (** p< = 0.01 (student’s *t*-test), N = 5 repeats) in *dhap40-ko* (lane 3) than in normal (WT, lane 1) background, and was absent in control flies lacking *nSyb*-Gal4 driver (lane 2). (L) The levels of HTT were about four times higher and HAP40 about three times higher in flies co-expressing HTT and HAP40 (lane 2) than flies expressing HTT (lane 3) or HAP40 (lane 1) alone, all driven by *nSyb*-Gal4. (M) Western blot assays for HTT and HAP40 levels in HEK293T cells with simultaneous co-expression of FLAG-tagged HTT-23Q and HTT-145Q, ran as three repeat experiments (N = 3) probed with anti-FLAG and anti-HAP40 antibodies, as indicated. Co-transfection with HAP40 led to an average of about two-fold increase of HAP40 and three- to five-fold increase of HTT-23Q and HTT-145Q (compare lane 4–6 with lanes 1–3). *** p< 0.001 (student’s *t*-test), which were calculated as the fold changes in HAP40 and HTT levels after co-transfection with HAP40 as compared to before HAP40 transfection.

Given that HAP40 binds full-length HTT (fl-HTT), but not its N-terminal region alone [35,36], we next tested another well-characterized fly HD model, which expresses mutant human fl-HTT with 128Q (fl-HTT-128Q) or wildtype control of fl-HTT with 16Q (fl-HTT-16Q) [52]. In addition, to ensure the reproducibility and minimize background variation, we also created a new set of UAS-based transgenic lines for human fl-HTT carrying 23 or 145 glutamines, with all the lines being inserted to the same attP40 integration site in the fly genome through the attB/Phi31 targeted integration method [53,54]. As expected, when tested using different Gal4 drivers, including the eye-specific GMR-Gal4 and pan-neuronal *elav*-Gal4 or *nSyb*-Gal4 drivers, wildtype fl-HTT (16Q or 23Q) did not induce any discernable detrimental effect, whereas fl-HTT-128Q and fl-HTT-145Q manifested strong toxicity, including early animal lethality and prominent eye degeneration, the latter being manifested as adult-onset gradual depigmentation and loss of internal photoreceptor cells (Figs 10A–10F and S9B–S9E). When tested using the full-length HTT-based HD models, loss of *dhap40* clearly ameliorated the neurodegenerative phenotypes in different assays. For example, in flies with eye-specific expression of wildtype fl-HTT-23Q, directed by the strong eye-specific GMR-Gal4 driver, their internal eye structure displayed highly regular composition and patterning, with seven photoreceptors organized in stereotypic pattern within each ommatidium unit (Fig 10A). In contrast, in age-matched flies expressing fl-HTT-145Q (Fig 10B), the eyes already manifested severe degeneration phenotypes even in young flies, showing prominent loss of photoreceptor cells and severe disintegration of ommatidia structure. However, such severe eye degeneration phenotypes were significantly suppressed by the absence of endogenous *dhap40* (Fig 10C), most of the eye units contained recognizable ommatidia in regular patterning and the presence of most photoreceptors inside each ommatidia unit, although many rhabdomeres still showed abnormally elongated morphology and some photoreceptors were clearly missing. Additionally, as these flies became older, flies with eye-specific expression of fl-HTT-145Q showed eye depigmentation phenotype due to the severe internal tissue loss (compare Fig 10E with age-matched 10D that expressed wildtype HTT-23Q). Again, loss of *dhap40* suppressed this depigmentation phenotype induced by fl-HTT-145Q (compare Fig 10F with 10E). Furthermore, we observed similar reduction in degeneration of internal neuronal photoreceptor cells in flies with pan-neuronal expression of fl-HTT-128Q driven by pan-neuronal *nSyb*-Gal4 (Fig 10G). Lastly, the adult lethality linked to pan-neuronal expression of mutant HTT was also delayed in the absence of *dhap40* (Fig 10H).

The significant protective effect conferred by the loss of endogenous *dhap40* would predict that higher levels of HAP40 should exacerbate the neurodegeneration of HD flies. Indeed, in flies with eye-specific expression of fl-HTT-145Q, directed by the strong eye-specific GMR-Gal4 driver, compared to flies co-expressing luciferase dsRNA control (S10A Fig), co-expression of human HAP40 significantly enhanced the eye degeneration phenotypes, causing prominent loss of photoreceptor cells and clear disruption of regular ommatidia patterning (S10B Fig). Interestingly, co-expression of fly dHap40 showed little effect (S10C Fig), likely due to its reduced binding affinity for human HTT (Fig 4E). Surprisingly, in another full-length HD model with fl-HTT-128Q-expressing driven by pan-neuronal expressing *nSyb*-Gal4, co-expression of human HAP40 only marginally enhanced the eye degeneration induced by the fl-HTT-128Q (Fig 10I). Moreover, the animal lethality associated with mutant fl-HTT was unchanged in the presence of the co-expressed HAP40 (Fig 10J). As expected, co-expression of HAP40 with wildtype HTT (fl-HTT-23Q or fl-HTT-16Q) did not elicit any apparent phenotypes in all the tissues examined (Figs 10G and 10I and S9).

In different animal models of neurodegenerative diseases, the severity of phenotypes mostly correlates with the expression levels of mutated disease proteins such as mutant HTT [52,55,56]. Therefore, the observed modifications by endogenous *dhap40* or the ectopically expressed human HAP40 could be due to their effect on overall HTT protein levels, especially considering the strong and conserved mutual dependence between HTT and HAP40 in maintaining each other’s stability (Fig 5). Accordingly, we examined whether the levels of human HTT, expressed ectopically from the transgenic flies, could be affected by the loss of endogenous *dhap40* or by the presence of co-expressed human HAP40. Indeed, when the same UAS-HTT transgene line was driven by pan-neuronal *nSyb*-Gal4, the levels of HTT in *dhap40-ko* mutants was less than half of that in wildtype background (Fig 10K). Conversely, co-expression of human HAP40 resulted in over three-fold increase of HTT levels than when HTT was expressed alone and strikingly, a similar increase of co-expressed HAP40 than when HAP40 was expressed alone (Fig 10L). These results implied that when expressed ectopically in transgenic flies, human HTT can be stabilized not only by human HAP40, but also by endogenous fly dHap40, an observation in line with the findings that fly dHap40 can bind, albeit at reduced efficiency, with human HTT (Fig 4E). Consistently, when tested in human cells, a similar synergistic effect was also observed between co-expressed HTT and HAP40. For example, in HEK293T cells that simultaneously co-expressed wildtype (23Q) and mutant (145Q) fl-HTT proteins, co-transfection of HAP40 led to significantly increased levels of both fl-HTT-23Q and fl-HTT-145 HTT proteins (compare lanes 4–6 with lanes 1–3 in Fig 10M and the quantification chart below).

Collectively, the above results demonstrate that HAP40-free mutant HTT largely retains it toxicity, if not more toxicity, in tested fly HD models. Additional studies in mammalian HD models are warranted to clarify the potentially important albeit complex role of HAP40 on mutant HTT-induced neurodegeneration in relevant physiological settings.

## Discussion

Despite the evidence implicating the importance of HAP40 on HTT regulation and HD pathogenesis, there has been little systematic examination of HAP40 in physiological and pathological settings. In this study, we isolated a fly homolog of HAP40 and further demonstrated its conserved physical and functional interactions with HTT. The co-existence of HAP40 and HTT in evolutionarily distant species from flies to humans not only supports the functional importance of HAP40 in HTT biology, but also establishes *Drosophila* as a relevant genetic model to evaluate the physiological and pathological roles of HAP40. Our results support that HAP40 is a central and positive regulator of HTT’s endogenous functions, and there exists a strong mutual-dependence between endogenous HTT and HAP40 on each other’s stability, a regulation that is highly conserved from flies to human cells. Lastly, our findings suggest that glutamine expansion in HTT does not significantly affect its affinity for HAP40, levels of HAP40 are not elevated in HD cells, and HAP40-free mutant HTT is still toxic. The robust and multiplex effect of HAP40 on HTT in models of fruit flies and cultured mammalian cells warrant further detailed studies in relevant physiological settings to elucidate HAP40’s roles in HTT functions and HD pathogenesis.

### HAP40 is a conserved binding partner of HTT

In the recent cryo-EM study on HTT structure, it was noted that a homology-based search failed to identify a HAP40 homolog in the *Drosophila* genome [36]. This is probably due to the overall low degree of sequence similarity between human HAP40 and fly dHap40, mirroring the significant sequence divergence between human and fly HTT from two very distant species [23]. However, our results clearly support that the previously uncharacterized *cg8134* gene encodes the fly orthologue of HAP40, as evident by its tight association with endogenous dHtt protein from an *in vivo* pull-down assay (Fig 2), by their overall structural similarities, especially their highly conserved N- and C-terminal regions known to be important for HTT binding (Fig 3), by the retained ability of dHap40 to physically interact with human HTT (Fig 4E) and stabilize ectopically expressed HTT in flies (Fig 10K), and lastly, by the ability of human HAP40 to rescue the loss of function phenotypes of *dhap40*-null flies (Fig 4D). It is still unclear to what extent their functions are conserved and whether fly dHap40 and dHtt can functionally replace HAP40 and HTT in mammals, as it is possible that HTT and HAP40 have gained certain new functions during evolution to serve the increasingly complex physiology and higher functions in mammals. Nevertheless, our findings support HAP40 and HTT as conserved partners co-existing through evolution from flies to mammals.

The importance of this conserved partnership between HTT and HAP40 is further reinforced by the findings of a highly conserved mutual dependence on each other’s stability between the two proteins. In both flies and human cells, loss of dHtt or HTT leads to an almost complete depletion of endogenous dHap40 or HAP40, respectively (Fig 5). Conversely, higher levels of HTT resulted in elevated levels of HAP40 (Fig 5D); and both in flies and human cells, their co-expression led to a synergistic, multi-fold increase of both proteins than when each was expressed alone (Fig 10L and 10M). These findings together support that complex formation with HAP40 is an important stabilizing mechanism for HTT. Collectively, our findings not only establish *Drosophila* as a relevant animal model for studying HAP40, but also uncover an ancient and important regulatory mechanism governing HTT that constrains the co-evolution of this HTT/HAP40 partnership through millions of years of evolution pressure in flies and humans.

The similarity between dHap40 and vertebrate HAP40 proteins exist at both sequence and structural levels (Fig 3A–3C), which not only form the basis of their functional conservation, but also implicate physiological significance of the conserved regions in HAP40 proteins. For example, the N- and C-terminal regions as well as residue E331 of human HAP40 are all highly conserved from *Drosophila* to vertebrates, supporting their roles in mediating direct physical interactions with HTT [36]. Conversely, consistent with its dispensable role in HTT binding, the central region of human HAP40 is not conserved in fly dHap40 and coincidentally is also invisible in the Cryo-EM imaging [36]. Among the other conserved regions in HAP40 (Fig 3A–3C), the 20 a.a.-long BΦ motif at the N-terminus is particularly interesting, as it is invisible in the Cryo-EM model, indicating its structural flexibility; However, its highly conserved and rather unique amino acid composition potentially suggest an important functional role. Similarly, the invariable proline repeats in-between the predicted α-helices 9 and 10 (Fig 3A–3C, prolines 282 to 284 in human HAP40) potentially introduce an important structural kink that facilitates the proper folding of HAP40 proteins. As an extrapolation, the size of HAP40 proteins is also relatively conserved, at around ~40kDa. Since within the HTT/HAP40 complex, HAP40 is sandwiched inside HTT [36], this raises an intriguing possibility that HAP40 in its folded conformation needs to stay within specific size range in order to fit in and prop up HTT into a unique shape or conformation, so as to properly execute its cellular functions. Future structural-functional study should help clarify the physiological significance of these observed conservations.

Finally, many studies on HTT and HD primarily focus on N-terminal HTT fragments, especially its exon 1, which lack the ability to physically interact with HAP40 [35,36]. Given that endogenous HTT mainly exists in a complex with HAP40 while HAP40-free HTT is functionally or activity-wise quite different from HTT/HAP40 complex (Figs 4 and 5), the HAP40-binding capacity of HTT constructs should be considered in future functional studies.

### HAP40 is an essential regulator of HTT

HTT has diverse cellular functions from transcription to autophagy [10,20]. Given that HAP40 is an essential HTT partner, it raises questions on whether HAP40 positively or negatively affects HTT’s cellular activities, and whether the two proteins always function together in the same processes or each mediates certain specific cellular activities. Identification of the HAP40 homolog in *Drosophila* affords us the opportunity to address these questions. Our studies in flies and human cells support that HAP40 might positively regulate HTT. Nevertheless, the overall phenotypes of *dhap40* null flies are weaker than that of *dhtt-ko* mutants, living on average 7 days longer and mobility declines slower than *dhtt-ko* flies. Although loss of HAP40 markedly reduced levels of endogenous HTT, significant amounts of HTT remained in both flies and human cells, which would suggest that the HAP40-free HTT is still partially functional, or alternatively they might mediate a distinct set of cellular functions independent of the HTT-HAP40 complex. Collectively, our findings support that HTT and HAP40 largely function together in the same set of cellular processes, and HAP40 is important for HTT’s normal physiological functions. Further, the two proteins are not functionally equal, as the primary role of HAP40 is likely to stabilize and regulate HTT, a hypothesis consistent with the observations that loss of HTT leads to an almost complete depletion of endogenous HAP40 in flies and human cell lines, while HTT alone is relatively more stable and likely still partially functional.

Our results also indicate that in the absence of HTT, most of HAP40 is degraded quickly through the proteasomal machinery (Fig 6). Conversely, in both *dhap40*-null flies and HAP40-knockout human 293 cells, lower but significant levels of HTT protein persist (Fig 5). Together, they implicate different turnover dynamics that govern these two proteins. Given HTT’s unusually large protein size, both its mRNA transcription as well as the synthesize, maturation and degradation of the final protein product are likely time-consuming and energy-intensive, therefore is regulated differently from the much smaller HAP40. Within the HTT/HAP40 protein complex, the levels of HTT might be a limiting factor than HAP40. Consistent with this hypothesis, in Fig 5D, when HTT was ectopically expressed in HTT-KO cells at levels that was significantly higher than that of endogenous HTT, there was a similarly increased level of endogenous HAP40 (compare lane 3 with lane 1 in Fig 5D). In contrast, in rescue experiment for *dhap40-ko* mutant flies (Fig 5E), where dHap40 was ectopically expressed from UAS-dHap40 transgene at much higher levels than endogenous dHap40, the endogenous dHtt levels was restored to levels similar to, but not higher than, that in wildtype flies (compare lane 4 with lane 1 in Fig 5E). Considering that HAP40 overexpression can prolong HTT’s half-life and reduce its turnover rate (Fig 7) while transient knockdown of HAP40 led to reduced levels of endogenous HTT (Fig 5K and 5L), they raise an attractive possibility that HTT exists in a constant equilibrium between a more stable HAP40-bound form and a degradation-prone HAP40-free form. Higher level of HAP40 tilts the equilibrium, resulting in higher proportion of HTT in complex in HAP40 and overall a slower turnover rate for HTT. Together, they support that HAP40 is an essential regulator of HTT stability and a potential target for HTT-lowering strategy.

### The role of HAP40 in HD pathogenesis

As an essential regulator of HTT, some key questions are how HAP40 affects the toxicity of mutant HTT, and whether the HAP40-free mutant HTT is more or less toxic. Contrary to an early report [35], we found that HAP40 levels were not elevated in HD cells (Fig 9), a result that was also observed in two recent reports [57,58]. Such a result would be a natural extension from our findings that polyQ expansion in HTT did not affect its binding with HAP40 (Figs 8 and S8) and their mutual dependence on protein stability (Fig 5), and that the majority, if not all, of HAP40 protein should be in complex with HTT, as most HTT-free HAP40 is quickly cleared away by the proteasome (Figs 5–7). Further, they are consistent with the earlier findings that mutant HTT largely retains its key physiological functions [59,60] and accordingly its ability to associate with its major binding partners including HAP40. Together, they imply that abnormal HAP40 accumulation is unlikely a pathogenic factor in HD.

When tested using fly models of HD, HAP40 did no apparently modulate the neurodegeneration induced by mutated HTT exon 1 (S9A Fig), likely due to the fact that HAP40 binds full-length but not truncated HTT [35,36]. However, surprisingly, despite its strong effect on the levels of full-length human HTT protein, neurodegeneration was only mildly affected by HAP40 in flies expressing full-length mutant HTT in certain circumstances (Fig 10). This rather weak modifying effect could suggest that HAP40 plays no meaningful role in HD pathogenesis. Alternatively, it could reflect a compounded effect of HAP40 on HTT regulation, such as on HTT’s physiological functions, its stability and overall protein levels, and potentially the potency of mutant HTT toxicity *per se*. First, normal functions of HTT are known to be neuronal protective, potentially arising from its roles from autophagy to trafficking and neurotrophic effect by BDNF and other neurotrophic factors [10,20]. Our results showed that HAP40 is a positive and essential regulator of HTT’s normal physiological functions in both flies and human cells (Fig 4). Depletion of HAP40 could therefore significantly compromise the neuroprotective activities of endogenous HTT. Second, HAP40 is essential for HTT’s stability and its overall protein levels (Figs 5 and 7), with levels of ectopically expressed HTT being elevated (Fig 10L and 10M) and its half-life extended (Fig 7H) in response to higher levels of HAP40, and conversely depletion of endogenous HAP40 simultaneously reducing the levels of endogenous HTT (Figs 5 and 7) or even the ectopically expressed human HTT in flies (Fig 10K). In animal HD models, the severity and progression of neurodegeneration often correlate with the levels of the expressed mutant HTT protein [52,55,56]. Considering that polyQ expansion did not affect HTT’s binding with HAP40 while HAP40 depletion lowered the levels of mutant HTT in HD cells (Fig 8), it is possible that HAP40 could affect HD pathogenesis by altering the total amount of mutant HTT protein. Lastly, HAP40 might directly affect the potency of mutant HTT toxicity *per se* by altering its folding and aggregating dynamics. It was shown that HAP40 exerts a strong impact on the conformation and biochemical property of HTT protein, as when expressed alone, full-length HTT was found to be in a conformational heterogeneous status, prone to oligomerize and aggregate [61], while HAP40-binding stabilized HTT into a homogenous and monomeric globular structure [36]. Although still a matter of debate, it has been postulated that the toxicity of mutant HTT is mainly due to its smaller aggregated species, while the large mutant HTT aggregates might be inactive or even play a potentially protective role [62–64]. Therefore, it is possible that HAP40-free mHTT is in a conformationally more labile and less stable but more toxic state, while HAP40-binding lowers its aggregating propensity, thereby converting mutant HTT to a structurally more homogenous and stable but less toxic conformation. In such a scenario, HAP40 might exert a double-edged effect on mutant HTT, reducing its potency of toxicity while increasing its total protein levels. This might explain why when driven by pan-neuronal *nSyb*-Gal4, despite several-fold increase of total HTT levels when co-expressed with HAP40, it did not translate into more severe neurodegeneration in fly HD model (Fig 10H–10M). Notably, among human HD patients, the rare HD homozygosity does not apparently lower the age of disease onset than the typical heterozygotes [65–67], implying a dosage threshold and potentially a saturating effect by mutant HTT in inflicting neuronal damage, which would discount against the postulated pathogenic role of HAP40 through its impact on the protein levels of mutant HTT. However, it was also reported that the phenotype and the rate of disease progression are more severe in HD homozygotes [66], implicating a dosage effect of mutant HTT protein on some aspects of HD pathogenesis. It is important to point out that all the experiments in our study were carried out in invertebrate *Drosophila* and cultured mammalian cells. More carefully studies in mammals under relevant physiological settings are needed to elucidate the exact roles of HAP40 in HTT regulation and HD pathogenesis, so as to take advantage of this conserved and highly specific HTT partner for novel therapeutic strategies such as “HTT-lowering”. Nevertheless, our results support that mutant HTT protein is toxic regardless of the presence of its binding partner HAP40 but its toxic potency might be modulated by HAP40.

## Materials and methods

### *Drosophila* husbandry and genetics

Fly stocks were maintained at room temperature following standard culture conditions. All fly crosses were performed at 25°C following standard genetic procedure unless otherwise specified. The following fly lines were from Bloomington Drosophila Stock Center: UAS-Htt.128Q.FL (#33808), UAS-Htt.16Q.FL (#33810), UAS-cas9 (58985), elav-Gal4 (#458), daughterless-Gal4 (#55850), nSyb-Gal4 (#51635), nanos-Gal4 (#4442).

UAS-Htt93Q-exon1 is a gift from Dr. Leslie Thompson.

### Genome tagging of *dhtt*

*dhtt* genome tagging constructs with C-terminal eGFP- and GA-TAP tags in pacman system were engineered following the established protocols [40, 68]. BacPac clone CH321-84B06 (BACPAC Resource Center (BPRC), Children’s Hospital Oakland Research Institute in Oakland, California), which covers all the genome coding regions of *dhtt* was selected as starting template for the tagging constructs [69]. An 82Kb genome region covering *dhtt* gene, which contains all the coding region of dhtt in addition to 28.5kb upstream of ATG start codon and 15kb downstream of the stop codon of the encoded dHtt protein, was cloned by recombineering. DNA templates for eGFP and GS-TAP tags were amplified by PCR and used for recombineering essentially as described [68, 69]. The purified DNA for the corresponding pacman tagging constructs were injected into phiC31*integrase-expresssing* embryos with VK22 attP integrate site at second chromosome, and transgenes were selected and established following standard protocols. The expression of tagged transgenes was validated by Western blots on protein extracts from adult transgenic flies (Figs 1 and 2).

### Creating *dhap40* mutant alleles

The mutant *dhap40* lines were generated following the established procedures [45]. Briefly, sgRNA1 (GGCAGCTAGATCCTTCCTAG) and sgRNA2 (GGTGCACTGCTATCACCGTG) targeting *cg8134* were cloned into pCFD6 vector and injected into fly embryo to generate corresponding transgenic lines. The resulting UAS-sgRNA 1, 2 were combined with UAS-cas9 and nanos-Gal4 for co-expression in fly germline. The male off-springs were selected and balanced to establish corresponding candidate mutant lines, and their genomic DNA were extracted and amplified using two primers: CAGCGTGGTCGATGTGCAG and CAGCGCCGAAACGAGTGG. For alleles with potential genome deletions, the protein extracts from the candidate homozygous flies were analyzed by Western blot assay to examine the absence of CG8134/dHap40 protein. The mutant lines with clear absence of CG8134/dHap40 protein were maintained for further characterization. Genome regions covering the established mutant alleles for *cg8134* gene were amplified by PCR followed by DNA sequencing to identify the molecular lesions within the *cg8134* gene. The confirmed *cg8134* null alleles were outcrossed with *w*^*1118*^ for 5 generations to establish stable fly lines with isogenic genetic background identical to the parental *w*^*11l8*^ line, before being employed for longevity and climbing assays.

### Molecular biology

pcDNA3-HTT-Q23-MYC (CH00038), pcDNA3-HTT-Q73-MYC (CH00039) and pcDNA3-HTT-Q145-MYC (CH00040) were from Coriell Institute deposited by the CHDI. The coding regions for HTT-Myc were released by select restriction enzymes and cloned into pUASTattB vector to generate corresponding transgenic fly lines.

For fly *cg8134*/*dhap40* overexpression, full-length cDNA clone for *cg8134* gene was digested from plasmid GH09650 (clone number 14278, Berkeley Drosophila Genome Project) by select restriction enzymes and cloned into pUAST vector to generate transgenic fly lines, or into pcDNA vector for transient expression by transfection in mammalian cells. For human HAP40 overexpression, full-length cDNA encoding human F8A1 (HAP40) was digested from pCS6(BC039693) plasmid (TCH1303, TransOMIC technologies) by restriction enzymes and cloned into pUAST vector for transgenic fly lines, or into pcDNA vector for transient expression by transfection in mammalian cells.

### Transgenic fly lines

For pUAST-HAP40 and pUAST-CG8134 (dHAP40) transgenes, the purified DNA were injected into *w*^*1118*^ embryos together with pπ25.7wc helper plasmid followed by standard transgenic procedures [70]. For pUASTattB-HTT-Q23, Q73 and Q145 transgenes and pacman-dhtt-GS-TAP and pacman-dhtt-eGFP tagging constructs, the purified DNA constructs were injected into phiC31*integrase-expresssing* embryos with VK22 attP integrate site at second chromosome, followed by standard transgenic procedures [40, 53, 54]. Embryo injection and transgene selection were carried out by Genetivision Co. (Houston, Texas).

### Antibodies

Primary antibodies were from the following sources: mouse anti-actin (1:10000, MAB1501, Chemicon and #ab-6276, Abcam); mouse anti-αTubulin (1:10000, DM1A, Sigma); mouse anti-Htt (MAB2166, Millipore. 1:1000); rabbit anti-Htt (D7F7, Cell Signaling #5656, 1:10,000); rabbit anti-F8A1/HAP40 antibody (HPA046960, Sigma or Novus NBP3-02964), mouse anti-HA (12CA5, Roche); mouse anti-mutant HTT (MW1, Developmental Study Hybridoma Bank); mouse anti-c-Myc (9E10, Santa Cruz); mouse anti-FLAG (M2, Sigma F3165); rabbit anti-dHtt and rabbit anti-dHap40 (this study), α-GAPDH (Sigma G9545, 1:5,000); mouse anti-Calnexin (CalNx, Cell Signaling #2433. 1:1,000), chicken anti-GFP (Aves. 1:10,000), mouse anti-SBP Tag (clone SB19-C4, Santa Cruz Biotechnology sc-101595). Alexa Fluor 488-Phalloidin (A-12379), Alexa Fluor 680-(A-21076) and Alexa Fluor 800-(926–32212) conjugated secondary antibodies for immunoblotting (1:10,000) were from Molecular probes-Invitrogen and LI-COR, respectively.

### Anti-dHtt and dHAP40 antibodies

To generate anti-dHtt antibody, the coding region corresponding amino acid 321–475 of dHtt protein was amplified by PCR and cloned into pET28b expression vector (Novagen). To generate anti-CG8134 antibody, the coding region of cg8134 gene was amplified by PCR and cloned into pET28b vector and verified by DNA sequencing. The constructs were transformed into BL21 *E*. *Coli* strain for inducible-expression of His-tagged proteins in bacteria. His-tagged dHtt and CG8134 protein fragments were purified by Nickle-beads following manufacturer’s instruction and used to immunize two rabbits (#1049 and #1050) for antibody production (Covance). The specificities of the antibodies were verified in Western blot assays for the absence of the 400kDa dHtt, or 40kDa CG8134/dHap40 protein bands in whole protein extracts from the corresponding *dhtt-ko* and *dhap40-ko* mutant flies, respectively.

### Affinity purification of dHtt and associated proteins (dHaps) and MS-Proteomics

For protein extraction, adult flies of the appropriate genotypes were grown in home-made population cages (45 cm-width, 45 cm-depth and 40-cm height) at room temperature (22°C). Overnight (0–20 hours) collections of fly eggs from each of the genotypes as described in the manuscript were harvested on 10 cm dishes with 1% agar and freshly-prepared wet yeast paste. After 1-minute of dechorionated in 50% home bleach followed by extensive wash using tap water, 1 gram of the eggs of the appropriate genotypes were mixed with 2ml of Lysis buffer (0.5% NP-40, 150mM KCl, 1mM EDTA 20mM Tris PH 7.5, with 1xProtease inhibitors and Phosphatase inhibitor cocktail (GenDepot)), and homogenized gently by douncer five times. The total protein concentrations of the lysates were measured by Bradford method, and the lysates were dilute to a final protein concentration at about 15–20 mg/ml. After centrifuge at 100,000g (Beckman Optima TL Ultracentrifuge) for 20 minutes twice at 4°C to remove all debris from the lysate, the supernatants were further processed according to the established procedures.

For GFP pulldown, 20 ul of slurry agarose GFP-Trap were added to the lysate, incubated for 60min at 4°C with constant rotation, followed by centrifugation to separate the supernatants from the beads. The collected agarose beads were washed quickly two times use NETN buffer (0.5% NP-40, 170mM NaCl, 1mM EDTA, 50mM Tris PH 7.3), then eluted with 25ul 0.1M Glycine (PH 2.5). The eluates were neutralized using 2.5ul 1M Tris (PH 7.0), followed by separation on 4–12% SDS-PAGE gradient gel (Nupage # 11062171–0033, Invitrogen) and visualized by standard CB staining. The protein bands were excised and eluted for MS to identify the target proteins.

The GS-TAP purifications were essentially as described [39]. Specifically, the cleared extract supernatants were supplemented with 400ul of 50% rabbit IgG bead suspension (Sigma A2909), followed by incubation at 4°C for 2 hours with constant rotation. After binding, IgG beads were spun down for 1 min at 500 g, washed with 10 ml of wash buffer (50 mM Tris pH 7.5, 5% glycerol, 0.2% IGEPAL, 1.5 mM MgCl_2_, 125 mM NaCl, 25 mM NaF, 1 mM Na_3_VO_4)_), and then rinsed with 400 ul TEV cleavage buffer (10 mM Tris pH 7.5, 100 mM NaCl, 0.1% IGEPAL, 0.5 mM EDTA, 1 mM DTT), followed by TEV cleavage using 3 ul of AcTEV protease (Invitrogen, # 12575–015) in 160 ul of TEV cleavage buffer with constant rotation at 16°C for 90 minutes. After TEV cleavage, the elutes were separated from IgG beads, added to 120 ul of pre-washed Streptavidin beads (Pierce, #53117) and incubated at 4°C for 45 min with constant rotation, followed by washing with 6 ml 1x TEV cleavage buffer. After the last wash, purified proteins were eluted with 200 ul of Elution buffer (2 mM biotin in 150mM NaCl, 10mM Tris Ph8.0, 0.5% NP-40).

### Cell culture and transient transfection

HEK293T or HeLa cells were maintained in DMEM medium (Mediatech) containing 10% fetal bovine serum (FBS, Invitrogen), 100 IU penicillin, and 100 μg/ml streptomycin. All cells were grown in a humidified incubator at 37°C with 5% CO2 and 95% air. Transfections of plasmid DNA were performed using Lipofectamine 2000 (Invitrogen) according to manufacturer’s instructions or for HEK293 cells by polyethylenimine (PEI) following standard procedure. For HTT and HAP40 protein degradation assays (Fig 6), cells were treated with 10 μM MG132, 10mM ammonium (NH4+) or 30uM chloroquine (CQ) for the duration of times as indicated. Cell lysates were prepared using NETN lysis buffer (20 mM Tris-HCl, pH 8.0, 100 mM NaCl, 0.5 mM EDTA, 0.5%(v/v) Nonidet P-40, 2.5 mM sodium pyrophosphate, 1 mM β-glycerolphosphate, 1 mM sodium orthovanadate, 1 μg/ml leupeptin, 1 mM phenylmethylsulfonyl fluoride) or CHAPS lysis buffer (CLB: 40 mM HEPES, pH 7.4, 2 mM EDTA, 10 mM pyrophosphate, 10 mM glycerophosphate, 0.3% CHAPS, protease inhibitors from Roche) as indicated.

The following cell lines were from the Coriell Institute and cultured following the suggested protocols from the vendor: (1) mouse striatal ST HDH Q111/111, ST HDH Q7/111, ST HDH Q7/7. (2) Human fibroblast cell lines for HD patients (GM21757, GM21756, GM09197, GM05539, GM04723 GM04857, GM04687, GM04855, GM04691, GM04849, GM04737, GM03621, GM04857, GM04281) and normal controls (GM04787, GM02189, GM04729, GM02149, GM02153 GM02169. GM04190 GM04204).

### siRNA silencing

Fibroblasts were seeded at 300.000 cells/well in 6-well plates in MEM (Thermofisher 21090055) + 15% +1X L-Glutamine + 1X Pen/Strep + 15% FBS medium. The next day, medium was replaced with growth medium without antibiotics. 150 pmol of the HTT- or HAP40- directed siRNA (Sigma. HTT: SASI_Hs01_00241076; HAP40: SASI_Hs01_00209460) was premixed with 5 μl of Lipofectamine RNAiMAX (Thermofisher 13778075) in 500μl Opti-MEM medium (Thermofisher 31985047) before being added to each well. Cells were collected at each time point, pelleted and stored at -80°C until use.

### Real-Time PCR

Total RNA was isolated using RNeasy Mini Kit (Qiagen 74,106) following manufacturer’s instructions, and RNA level was quantified using Nanodrop 2000 (ThermoFisher). qPCR was performed in a 384-well plate using QuantiTect Probe RT-PCR Kit (Qiagen 204,443) with Random primers according to the manufacturer’s instruction. For each sample, 40 ng of total RNA was used. Each sample was in triplicate, each experiment was performed three times (N = 3). The TaqMan probes (ThermoFisher) used are: HTT: Hs00918174_m1; HAP40: Hs01058731_s1; GAPDH: Hs02786624_g1. Fold changes were calculated by ΔΔCt method using Time 0 samples as reference. GAPDH was used as housekeeping gene.

### Stable HTT-Q23 and HTT-Q145 expressing cell lines

pCDNA3.1-HTT-23Q-FLAG, pCDNA3.1-HTT-73Q-FLAG and pCDNA3.1-HTT-145Q-FLAG from Corielle Institute was digested with PuvI. After purification, the linearized plasmids were transfected in HEK293 cells with Lipofectamine 3000 followed manufacturer’s protocol. 48 hours after transfection, cells were selected with 400 ug/ml G418 for and single colonies were picked for expression testing.

### HTT and HAP40 knockout cell lines by CRISPR/Cas9

Single or double knockout cell lines for HTT and HAP40 in HEK293 and HeLa cells were created by CRISPR/Cas9 method essentially as described [71]. For HTT-KO, the following sgRNAs were cloned into eSpCas9(1.1)_No_FLAG_ATP1A1_G2_Dual_sgRNA vector (Addgene): HTT-sgRNA1 (GAAGGACTTGAGGGACTCGA) and HTT-sgRNA4 (ATGACGCAGAGTCAGATGTC), or HAP40-SgRNA (GTGGCCAGGAGCCTCCGCCC). The corresponding constructs were transfected into HEK293 or HeLa cells, followed by Ouabain selection three days after transfection and expansion of single cell clones in 24-well plates as described [71]. The established KO cell lines were validated by Western blot assays with anti-HTT (mAb2166, Millipore) or anti-HAP40 (HPA046960, Sigma) antibodies for the absence of endogenous HTT or HAP40 proteins, respectively.

### Pulse-Chase experiments

HEK293T cells were seeded at 500,000 cells/well in MW6 plates. After 1 day cells were starved in DMEM -Cys -Met for 15’ and then labelled for 15’ in DMEM -Cys -Met + 0.2 mCi/ml EasyTAG. Cells were washed 1x with unlabeled media and then at each time point cells were collected and directly lysed in PBS+ 1%TritonX100 + Protease Inhibitor Cocktail (Roche). 300 μg total cell extracts were pre-cleared with 30 ul Protein G Dynabeads for 1h at 4°C. 30 μl of Protein G Dynabeads were incubated with 1 μg αHTT D7F7 at RT for 1h in PBS 0.1% Tween-20 and washed 2x with PBS 0.1% Tween-20. Pre-cleared lysates were then incubated with Protein G-D7F7 complex at 4°C for 2 hrs. ProtG-Ab-Ag complexes were then passed through 30% Sucrose cushion in 0.15% SDS NDET (1% NP-40, 0.4% Na-Deoxycholate, 66mM EDTA, 10 mM Tris-HCl pH7.4), washed 2x in 0.3% SDS NDET and finally eluted in 25 ul Laemmli Buffer (95°C x 5’). Eluates were loaded in a 4–12% SDS Page Gel. Western Blot was performed by αHTT D7F7 **(**dilution in 1:10000 in TBST 5% BSA). HRP-conjugated α-rabbit (1:3000) was used as the secondary antibody. ARG was obtained directly exposing dried WB membrane for indicated times. Images were acquired by ChemiDoc (Biorad). Densitometry is expressed as the percentage with respect to Time 0 of the ratio between HTT ARG and HTT WB.

To test HAP40 overexpression effect, HEK293T cells were seeded at 300,000 cells/well in MW6 plate. Next day cells were transfected with Lipofectamine 2000 according to the manufacturer’s instructions with Empty Vector pcDNA3.1(+) (EV) or pcDNA3.1(+)/HAP40-FLAG. Next day the cells were pulsed as above.

### Bright field imaging of adult fly eyes

Adult flies of appropriate genotypes were orientated on glass slides with nail polish. Z-stack scanning of adult eyes were recorded under the 10X objective using Zeiss Axioimager Z1 microscope (usually 10–20 layers scanned) and reconstructed into 3D projection using CZFocus software.

### Dissection and staining of adult eye retina

Dissecting eye retina was performed as described [72]. Briefly, female flies of specific genotypes were beheaded. Then their heads were torn open from their proboscis to let 4% paraformaldehyde go in. After those heads were fixed for 1 hr at room temperature, heads were washed briefly with PBS for 2 times. Their retinas were carefully removed from the corneal lens layer using a fine forceps and a tungsten hook. The dissected retinas were washed with PBS for three times, 20 mins each, and stained by Phalloidin 488 (1:1000, Molecular Probes, Cat#: A12379) for 16 hrs at 4 degrees. Next day the samples were washed by PBS for three times, 20 mins each before being mounted and imaged using a confocal microscope (Leica DM6000-B).

### Climbing assay

Climbing assays were performed as described previously [22]. Briefly, 20 female flies were placed into a clear fly vial, gently tapped to the bottom of the vial, and then given 18 seconds to climb a 5 cm vertical distance. Flies that successfully did so were counted and their ratio at each time point were analyzed by t-test. Each fly group was tested every 7 days and transferred into a new vial with fresh food every 3–4 days.

### Longevity assay

Viability assays were performed as described previously [22]. Briefly, 20 newly hatched male or female flies of a specific genotype were placed into individual vials with fresh fly food at 25°C. The number of dead flies was recorded every 3 days until all flies died. Flies were transferred into a new vial with fresh food every 3 days to prevent them from sticking to old food or becoming dehydrated. The survival curve for each genotype were analyzed by log-rank test.

### Pseudopupil assay of adult fly eyes

Pseudopupil assay of adult fly eyes were performed as described [73]. Briefly, flies of a specific genotype were oriented and affixed in nail polish. The eyes were immersed in lens oil (Resolve Microscope Immersion oil, Thermo Scientific, Cat# M5000) and imaged through a 40X oil microscope lens (Zeiss, AX10). For each fly head, the rhabdomeres in 15–20 ommatidia were counted. Usually, 10–20 flies for each genotype were analyzed by Mann-Whitney rank sum test.

### Biochemistry

#### Western blotting

Standard 10% to 14% SDS-PAGE gels were used for separation of most proteins except for HTT, which was better analyzed by NuPAGE Tris-Acetate gels from Invitrogen specially formulated for detection of proteins with large molecular weight. The boiled samples were separated on SDS-PAGE and transferred to nitrocellulose membranes from Millipore. After blocking with 5% nonfat milk in Tris-buffered saline with 0.1% Tween-20 for 1 hour, membranes were incubated with primary antibodies. Secondary antibodies conjugated with Alexa-800 or Alexa-680 (Invitrogen) or HRP (KPL) were used and the signals were detected by the Odyssey Infrared Imaging System and quantified by Odyssey Application Software 3.0 or by densitometry of the digital images using ImageJ software (NIH).

#### Co-immunoprecipitation (co-IP)

Co-IP were performed as described. Specifically, transiently transfected HEK293T cells in 100 mm dishes were lysed in a NETN or CLB as indicated, sonicated three times for 5 sec each, and centrifuged at 14,000 rpm for 30 min at 4°C. Epitope-tagged proteins or endogenous proteins were immunoprecipitated from the cell lysate with anti-HA, anti-Myc, anti-FLAG, anti-Htt, and anti-HAP40 antibodies and Protein A/G Plus-agarose beads (sc-2003, Santa Cruz) as indicated. Immunoprecipitates or whole cell extracts (WCE) were analyzed by standard Western blotting.

### Statistics analysis and data acquisition

The statistical significance of the difference between experimental groups was determined by log-rank test, or Mann-Whitney U test, or two-tailed unpaired Student’s *t*-test, or one-way analysis of variance followed by Bonferroni *post hoc* test, as specified in corresponding legends. Where multiple comparisons were performed, we used normalization to control values. Differences were considered significant for *P*<0.05 and noted in the figures as *, *P*<0.01 noted in the figures as ** and *P*<0.001 noted as ***, or as specified in corresponding legends figures. Data are presented as mean+s.e.m from a minimum of three independent experiments. The exact sample size (n) is indicated in each figure and it corresponds to individual experiments unless otherwise stated. All the experiments were done at least 3 times and in duplicate or triplicate to account for technical variability.

For the studies in *Drosophila*, sample group allocation was based on genotype and the genotypes were blinded to the observed except for those cases in which tissues from different animals have to be pooled.

## Supporting information

S1 Fig Expression of dHtt-eGFP protein from pacman-dhtt-eGFP transgenic flies. Western blot for whole animal homogenates from two independent fly lines transgenic for pacman-dhtt-2-eGFP. Samples were double-probed with α-GFP (left panel) to detect dHtt-eGFP expressed from the transgene and α-dHtt antibody (middle panel) to reveal both endogenous dHtt protein and tagged dHtt-eGFP expressed from the transgene. Note that due to their large sizes, the tagged dHtt-eGFP can only be slightly separated from the smaller endogenous dHtt protein, and two bands largely overlap (right panel, green for α-eGFP and red for α-dHtt, note the overlaying yellow signal). The protein levels of the upper-half dHtt band (representing dHtt-eGFP from the transgene) is similar as the lower-half of dHtt band (representing endogenous dHtt), suggesting similar levels of protein expression between endogenous dHtt and dHtt-eGFP from the transgene. Two background bands in each channels from anti-HTT or anti-GFP antibodies served as loading controls.
(TIF)

S2 Fig A. The N- and C-terminal regions of HAP40 are most conserved between CG8134 (dHap40) and vertebrate HAP40. Sequence alignment of the N- and C-terminal regions between CG8134 and HAP40 homologs from human, mouse, frog and zebra fish, as indicated. The amino acid positions of the corresponding boundaries are labeled accordingly. Amino acids that are identical to CG8134 are highlighted in black and with similar chemical properties highlight in color. B. Sequence alignment between wildtype and dhap40 mutant alleles around the molecular lesions in established dhap40 alleles. C-F. The molecular lesions in four established dhap40 alleles. Genome sequence of exon 2 region of cg8134 gene, in which the exact molecular lesions of the four validated dhap40 mutant alleles, ko3, ko7, ko8 and ko9, are labeled in S2C-F, respectively, as indicated.
(PDF)

S3 Fig Validation of anti-CG8134 (dHap40) antibodies. Western blot assay against purified CG8134 proteins using serum from (A) rabbit #1 (ID number as 1049) and (B) rabbit #2 (ID number as 1050) that had been immunized with purified CG8134 protein expressed from bacteria. The quantify of purified CG8134 protein (ng) loaded into each lane were indicated on the top of the gels. Pre-immunization serum (pre-serum) from the same animals were used as controls. Anti-CG8134 sera from both animals, but not control of pre-immunization sera, could robustly detect the purified CG8134 protein. The cyan color indicates over-saturation of the detected signals that were above the sensitivity limit of the LiCoR laser scanner used for detection of far-red fluorescent signal.
(TIF)

S4 Fig High levels of dHap40 or human HAP40 proteins are not toxic in *Drosophila*. (A and B) Representative confocal images of whole-mount retina with phalloidin staining, dissected from 40-day-old adult flies with pan-neuronal expression of (A) dHap40 or (B) human HAP40 from UAS-derived transgenic flies, as indicated. Genotypes: (A) elav-Gal4/+>UAS-dHap40/+. (B) elav-Gal4>UAS-HAP40, all 40-day-old females. Flies from both genotypes showed the normal composition and organization of the seven photoreceptors within each ommatidium unit, suggesting that overexpression of dHap40 or human HAP40 in neurons were not toxic. More than 70 flies were examined for each genotype. (C and D) Representative bright-field images of 30-day-old adult fly eyes expressing (C) luciferase-dsRNA control or (D) human HAP40 (F8A1) from respective UAS-transgenes, both directed by strong eye-specific GMR-Gal4 driver. More than 90 flies were examined for each genotype. (E and F) Representative confocal images of whole-mount retina with phalloidin staining, dissected from 40-day-old adult flies with pan-neuronal expression of (E) human HAP40 or (F) fly dHap40 driven by pan-neuronal nsyb-Gal4 driver. Genotypes; (E) nsyb-Gal4/+ >UAS-HAP40/+. (F) nsyb-Gal4/+ >UAS-dHap40/+. Both showed normal composition and organization of the seven photoreceptors in each of the eight ommatidium units within the image field. More than 4 flies were examined and imaged for each genotype.
(TIF)

S5 Fig Autophagy inhibitors do not affect HTT degradation in HAP40-KO cells. Western blot assays and quantifications for endogenous HTT proteins in HAP40-KO or wildtype (WT) HEK293 cells under different treatments, as indicated. (Bottom) Normalized levels of HTT proteins from three repeat experiments. Treatment with autophagy inhibitor 3-MA or Bafilomycin A1 for 5 hours showed no clear effect on the levels of endogenous HTT protein in two independent HAP40-KO cells (N = 3 repeats for all the experiments). n.s., no significance. Actin served as loading and normalization controls in all the experiments.
(TIF)

S6 Fig Pulse-chase experiments to measure the turnover rates of endogenous HTT protein. (A) Schematics of pulse-chase experiments. HEK293 cells were starved in methionine-free medium for 30 minutes, then were supplemented with medium containing ^35^S-methionine for another 15 minutes to label the newly synthesized proteins. After the pulse labeling, the cells were washed and then maintained in regular medium for indicated intervals before harvesting for further analysis. (B) Endogenous HTT protein were enriched by immunoprecipitation with D7F7 anti-HTT antibody and resolved by SDS-PAGE separation, followed by autoradiography (ARG) and Western blot assays to measure the amount of ^35^S-labeled and total HTT protein at each time point, as indicated. (C) Turnover rate of endogenous HTT protein in control and HAP40-overexpressing cells, which was quantified as the relative levels of remaining ^35^S-labeled HTT at each time point after the start of the chase, all normalized against total HTT from each pulldown. Half-life for endogenous HTT at ~65 ± 5hrs hours (repeat N = 3).
(TIF)

S7 Fig PolyQ expansion in HTT does not affect its binding with HAP40. (A) Time course analyses of endogenous HAP40 and ectopically expressed HTT-23Q, 73Q or 145Q in wildtype HEK293 cells, hours after transfection with FLAG-tagged HTT expressing plasmids, as indicated. (B) Quantification of the time-dependent changes of HAP40 levels normalized against HTT at each time point, averaged from three repeat experiments.
(TIF)

S8 Fig The levels of HAP40 protein are lower in HD cells. (A and B) Two independent Western blot assays of endogenous HTT and HAP40 proteins in additional five normal and eight HD human fibroblast cells, as indicated. In (A), the membrane was co-probed with both anti-total HTT (D7F7, green) and mutant HTT specific (MW1, red) antibodies. Note that in (A), MW1 detected mutant HTT bands in all the eight HD cell lines, but not in the five wildtype cell lines, and these mutant HTT bands were also positive for anti-total HTT (D7F7, green) antibody but several appeared to migrate slightly slower than wildtype HTT from the same cells. The human fibroblast cell lines tested are: GM02149 (WT-4), GM02153 (WT-5), GM02169 (WT-6), GM04190 (WT-7), GM04204 (WT-8), GM04687 (HD-6), GM04855 (HD-7), GM04691 (HD-8), GM04849 (HD-9), GM04737 (HD-10), GM03621 (HD-11), GM04857 (HD-12, CAG repeats reported as 50 and 40), and GM04281 (HD-13). (C and D) quantification of (C) endogenous HTT and (D) HAP40 levels in these cell lines, normalized against loading control Actin from three independent experiments. *p< = 0.05, **p< = 0.01, ***p< = 0.001 (student’s *t*-test). n.s., no significance. β-Actin served as loading and normalization controls in all the experiments.
(TIF)

S9 Fig **A. HAP40 does not modulate neurodegeneration induced by mutant HTT exon1 fragment in *Drosophila***. Loss of endogenous *dhap40* did not have apparent effect on neurodegenerative phenotypes induced by HTT-exon1-93Q. Bar chart presentation of the average number of intact photoreceptor cells (PRC) per ommatidium in 7-day-old female flies of the following genotypes: *“elav-*Gal4/+; *UAS-*HTT-exon1-93Q/+*”*: 6.4 PRC/ommatidium (n = 12 flies); “*dhap40*^*ko3*^, *elav-*Gal4/+; *UAS-*HTT-exon1-93Q/+”: 6.4 PRC/ommatidium (n = 12 flies). The difference between the two is insignificant (p = 0.6 in rank-sum test). Flies in the study were cultured at 21°C. **B-D. HTT-145Q induces age-dependent neurodegeneration in *Drosophila* eye.** Bright-field images of adult fly eyes expressing full-length human HTT (HTT) with expanded (145Q, B and C) or wildtype (23Q, D) length of polyQ track, at young (7-day-old, B1 and B2) or old (30-day-old, C and D) ages, as indicated. Note the apparent de-pigmentation (green arrows), suggesting underneath cell death, only in old (C) but not young (B) flies expressing mutant HTT-145Q, and not in controls of wildtype HTT-23Q (D) at old age. Two representative flies for each indicated genotypes and ages were presented. Genotypes: (B and C) GMR-Gal4/+; UAS-HTT-145Q /+. (D) GMR-Gal4/+; UAS-HTT-23Q. More than 30 flies were examined for each genotypes. Note that the same S9D1 Fig eye image was also used in Fig 10D as control. **E. Neuronal expression of HTT-Q145 but not HTT-Q23 induces neurodegeneration and causes early death of the animals.** Survival curves of flies with pan-neuronal expression of full-length human HTT transgenes driven by pan-neuronal elav-Gal4 driver. Note that flies expressing mutant HTT (HTT-145Q, orange color) die significantly earlier than those expressing wildtype HTT (HTT-23Q, blue line). Genotypes: elav-Gal4/+; UAS-HTT-Q145 /+ (orange line, n = 79). elav-Gal4/+; UAS-HTT-23Q/+ (blue line, n = 84).
(TIF)

S10 Fig HAP40 affects neurodegeneration induced by full-length mutant HTT. (A-C) Representative confocal images of whole-mount retina with phalloidin staining, dissected from 1-day-old adult flies with eye-specific co-expression of human mutant HTT-145Q with (A) control luciferase dsRNA, (B) human HAP40 or (C) fly dHap40, all directed by GMR-Gal4 driver. In control (A), co-expression of mutant HTT-145Q with luciferase dsRNA caused abnormal ommatidium units with elongated, fragmented and partial loss of rhabdomere structures in most ommatidia units. In contrast, (B) co-expression with human HAP40 significantly enhanced the degeneration phenotypes, showing significant loss of photoreceptor cells and more prominent deformation of ommatidia structure, while (C) co-expression with fly dHap40 did no significantly affect the phenotypes. Genotypes: (A) GMR-Gal4/+ >UAS-HTT-145Q/+; UAS-Luciferase dsRNA/+. (B) GMR-Gal4/+ >UAS-HTT-145Q/+; UAS-HAP40/+. (C) GMR-Gal4/+ >UAS-HTT-145Q/+; UAS-dHap40/+. (D and E) Survival curves of the adult flies expressing full-length human HTT-128Q. (D) Average life span was 17 days for “*dhap40* ^*ko3*^*; nsyb-*Gal4/UAS-HTT-128Q” flies (n = 179) and 15 days for control *“nsyb-*Gal4/UAS-HTT-128Q” flies (n = 229). The difference between the two is significance (p<0.001. Log-rank test). (E) Average life span was 24.3 days for flies co-expressing HTT-128Q with HAP40 (genotype: “*nsyb*-Gal4>UAS-HAP40/+; UAS-HTT-128Q/+”. n = 141), and 24.5 days for control flies co-expressing HTT-128Q with luciferase-dsRNA (“*nsyb*-Gal4> UAS-luciferase-RNAi/+; UAS-HTT-128Q/+”, n = 174), with no significant difference between the two genotypes (p = 0.9 by Log-rank test).
(TIF)
