# Supplementary figures and images for: HAP40 is a conserved central regulator of Huntingtin and a potential modulator of Huntington’s disease pathogenesis
Source: PLoS Genet. 2022 Jul 19;18(7):e1010302. doi: 10.1371/journal.pgen.1010302 (PMC9295956; doi:10.1371/journal.pgen.1010302)

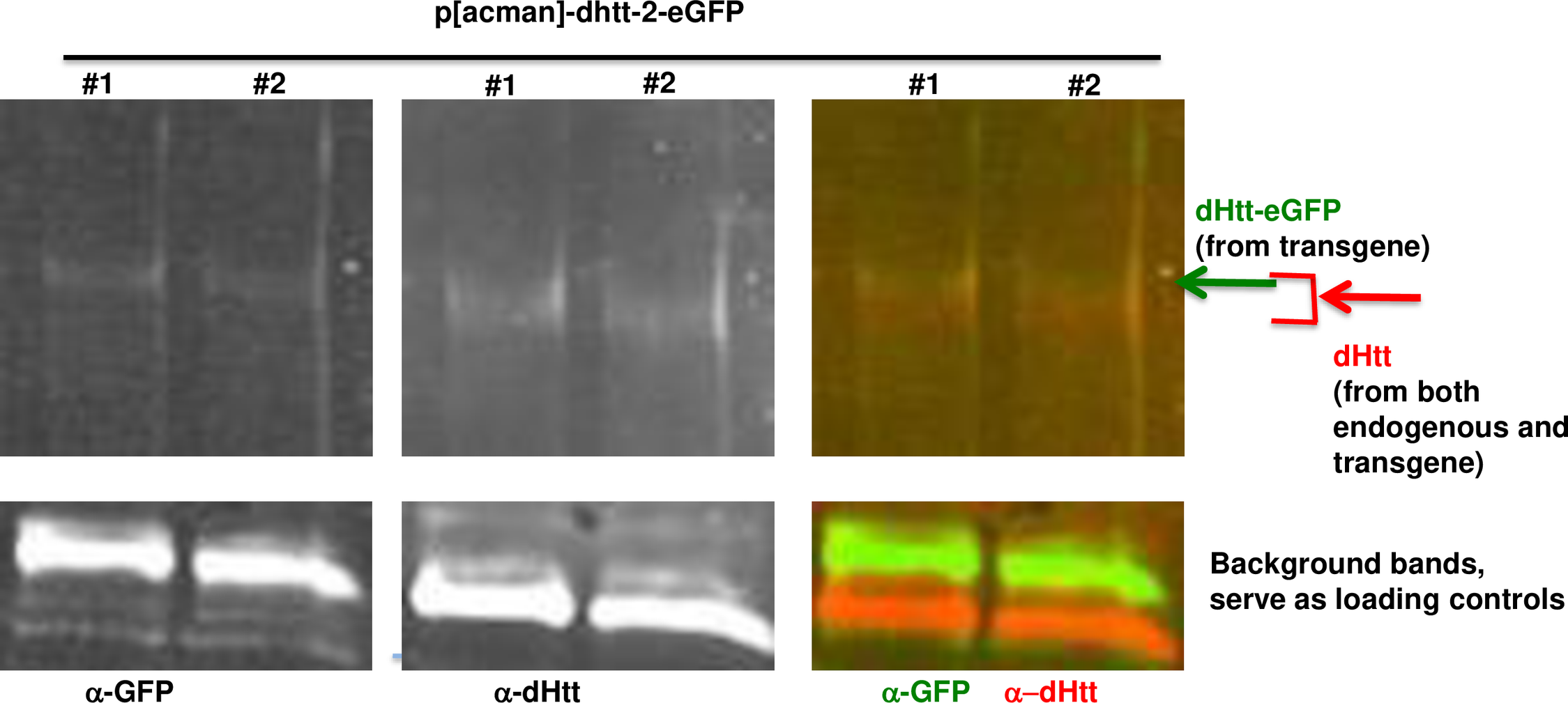

Supplement: S1 Fig — Western blot for whole animal homogenates from two independent fly lines transgenic for pacman-dhtt-2-eGFP. Samples were double-probed with α-GFP (left panel) to detect dHtt-eGFP expressed from the transgene and α-dHtt antibody (middle panel) to reveal both endogenous dHtt protein and tagged dHtt-eGFP expressed from the transgene. Note that due to their large sizes, the tagged dHtt-eGFP can only be slightly separated from the smaller endogenous dHtt protein, and two bands largely overlap (right panel, green for α-eGFP and red for α-dHtt, note the overlaying yellow signal). The protein levels of the upper-half dHtt band (representing dHtt-eGFP from the transgene) is similar as the lower-half of dHtt band (representing endogenous dHtt), suggesting similar levels of protein expression between endogenous dHtt and dHtt-eGFP from the transgene. Two background bands in each channels from anti-HTT or anti-GFP antibodies served as loading controls. (TIF) [file pgen.1010302.s001.tif]

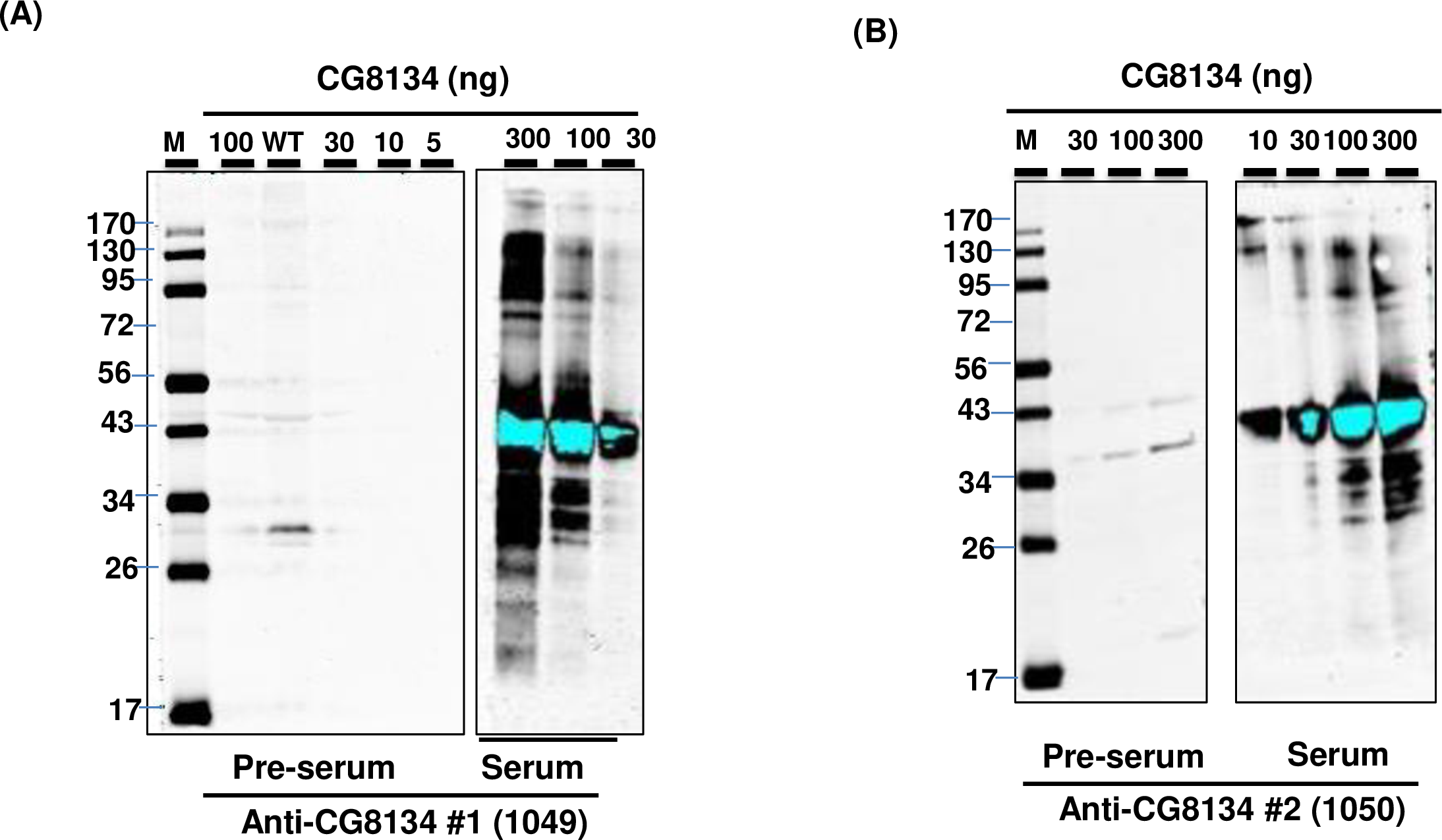

Supplement: S3 Fig — Western blot assay against purified CG8134 proteins using serum from (A) rabbit #1 (ID number as 1049) and (B) rabbit #2 (ID number as 1050) that had been immunized with purified CG8134 protein expressed from bacteria. The quantify of purified CG8134 protein (ng) loaded into each lane were indicated on the top of the gels. Pre-immunization serum (pre-serum) from the same animals were used as controls. Anti-CG8134 sera from both animals, but not control of pre-immunization sera, could robustly detect the purified CG8134 protein. The cyan color indicates over-saturation of the detected signals that were above the sensitivity limit of the LiCoR laser scanner used for detection of far-red fluorescent signal. (TIF) [file pgen.1010302.s003.tif]

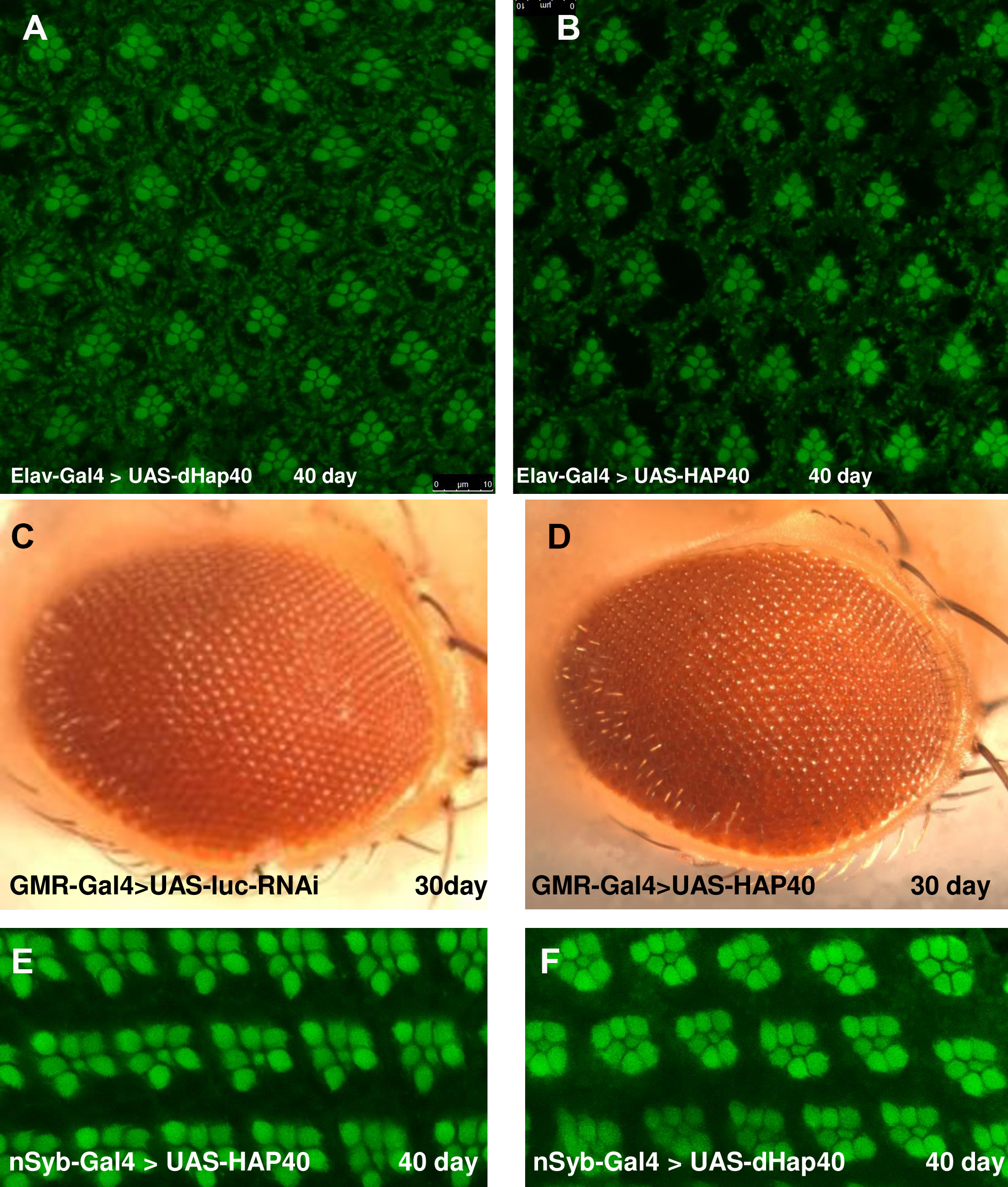

Supplement: S4 Fig — (A and B) Representative confocal images of whole-mount retina with phalloidin staining, dissected from 40-day-old adult flies with pan-neuronal expression of (A) dHap40 or (B) human HAP40 from UAS-derived transgenic flies, as indicated. Genotypes: (A) elav-Gal4/+>UAS-dHap40/+. (B) elav-Gal4>UAS-HAP40, all 40-day-old females. Flies from both genotypes showed the normal composition and organization of the seven photoreceptors within each ommatidium unit, suggesting that overexpression of dHap40 or human HAP40 in neurons were not toxic. More than 70 flies were examined for each genotype. (C and D) Representative bright-field images of 30-day-old adult fly eyes expressing (C) luciferase-dsRNA control or (D) human HAP40 (F8A1) from respective UAS-transgenes, both directed by strong eye-specific GMR-Gal4 driver. More than 90 flies were examined for each genotype. (E and F) Representative confocal images of whole-mount retina with phalloidin staining, dissected from 40-day-old adult flies with pan-neuronal expression of (E) human HAP40 or (F) fly dHap40 driven by pan-neuronal nsyb-Gal4 driver. Genotypes; (E) nsyb-Gal4/+ >UAS-HAP40/+. (F) nsyb-Gal4/+ >UAS-dHap40/+. Both showed normal composition and organization of the seven photoreceptors in each of the eight ommatidium units within the image field. More than 4 flies were examined and imaged for each genotype. (TIF) [file pgen.1010302.s004.tif]

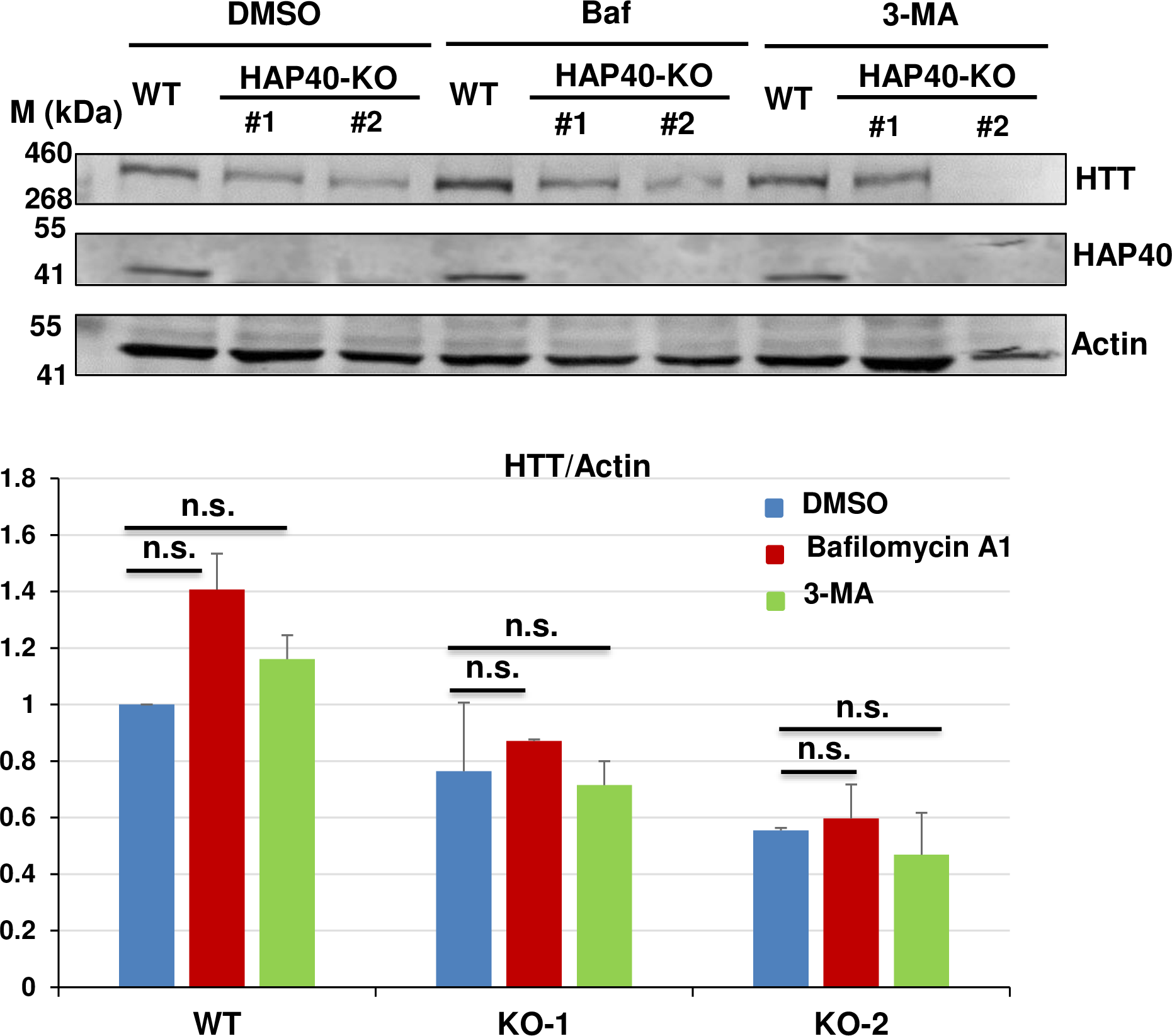

Supplement: S5 Fig — Western blot assays and quantifications for endogenous HTT proteins in HAP40-KO or wildtype (WT) HEK293 cells under different treatments, as indicated. (Bottom) Normalized levels of HTT proteins from three repeat experiments. Treatment with autophagy inhibitor 3-MA or Bafilomycin A1 for 5 hours showed no clear effect on the levels of endogenous HTT protein in two independent HAP40-KO cells (N = 3 repeats for all the experiments). n.s., no significance. Actin served as loading and normalization controls in all the experiments. (TIF) [file pgen.1010302.s005.tif]

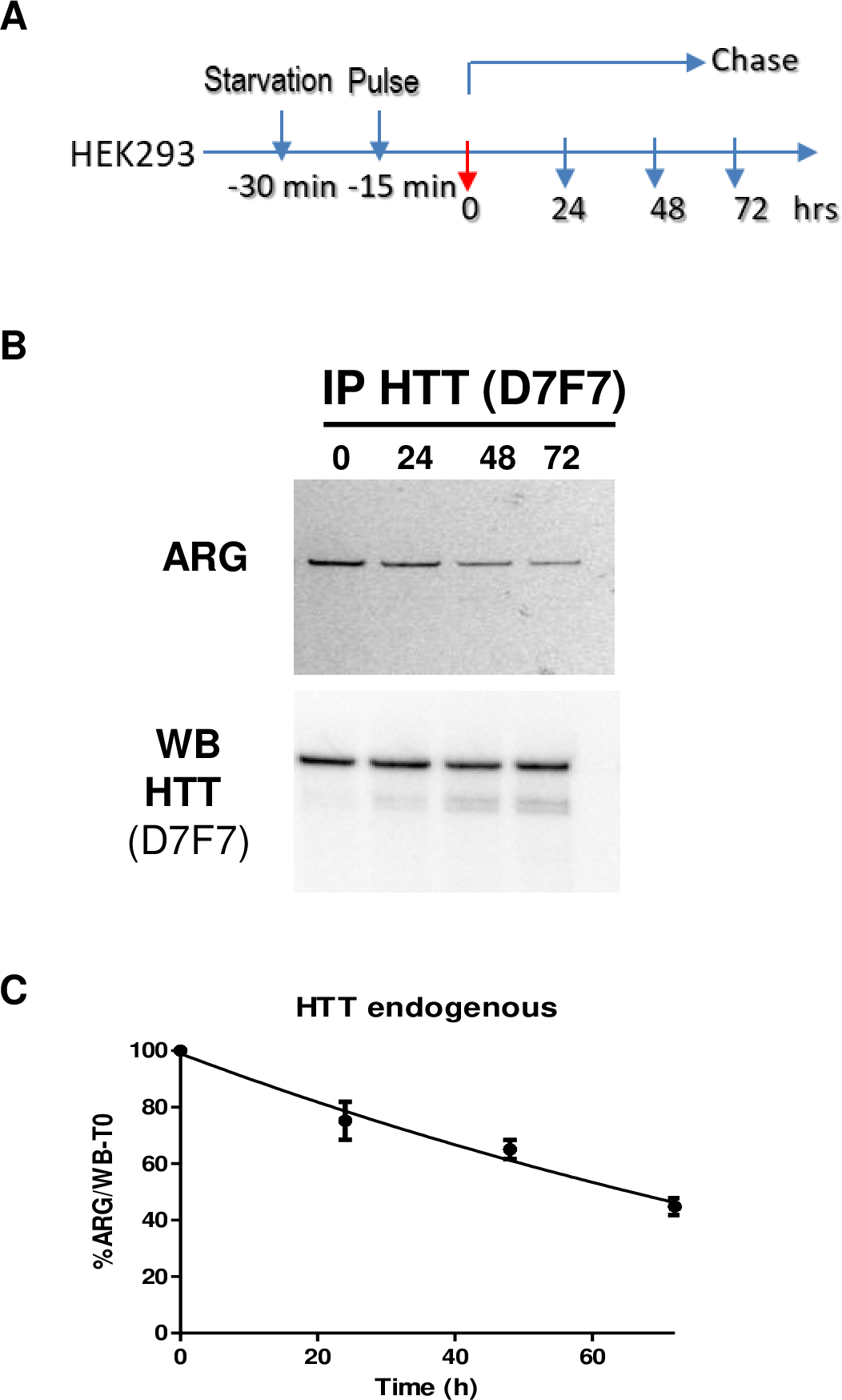

Supplement: S6 Fig — (A) Schematics of pulse-chase experiments. HEK293 cells were starved in methionine-free medium for 30 minutes, then were supplemented with medium containing 35S-methionine for another 15 minutes to label the newly synthesized proteins. After the pulse labeling, the cells were washed and then maintained in regular medium for indicated intervals before harvesting for further analysis. (B) Endogenous HTT protein were enriched by immunoprecipitation with D7F7 anti-HTT antibody and resolved by SDS-PAGE separation, followed by autoradiography (ARG) and Western blot assays to measure the amount of 35S-labeled and total HTT protein at each time point, as indicated. (C) Turnover rate of endogenous HTT protein in control and HAP40-overexpressing cells, which was quantified as the relative levels of remaining 35S-labeled HTT at each time point after the start of the chase, all normalized against total HTT from each pulldown. Half-life for endogenous HTT at ~65 ± 5hrs hours (repeat N = 3). (TIF) [file pgen.1010302.s006.tif]

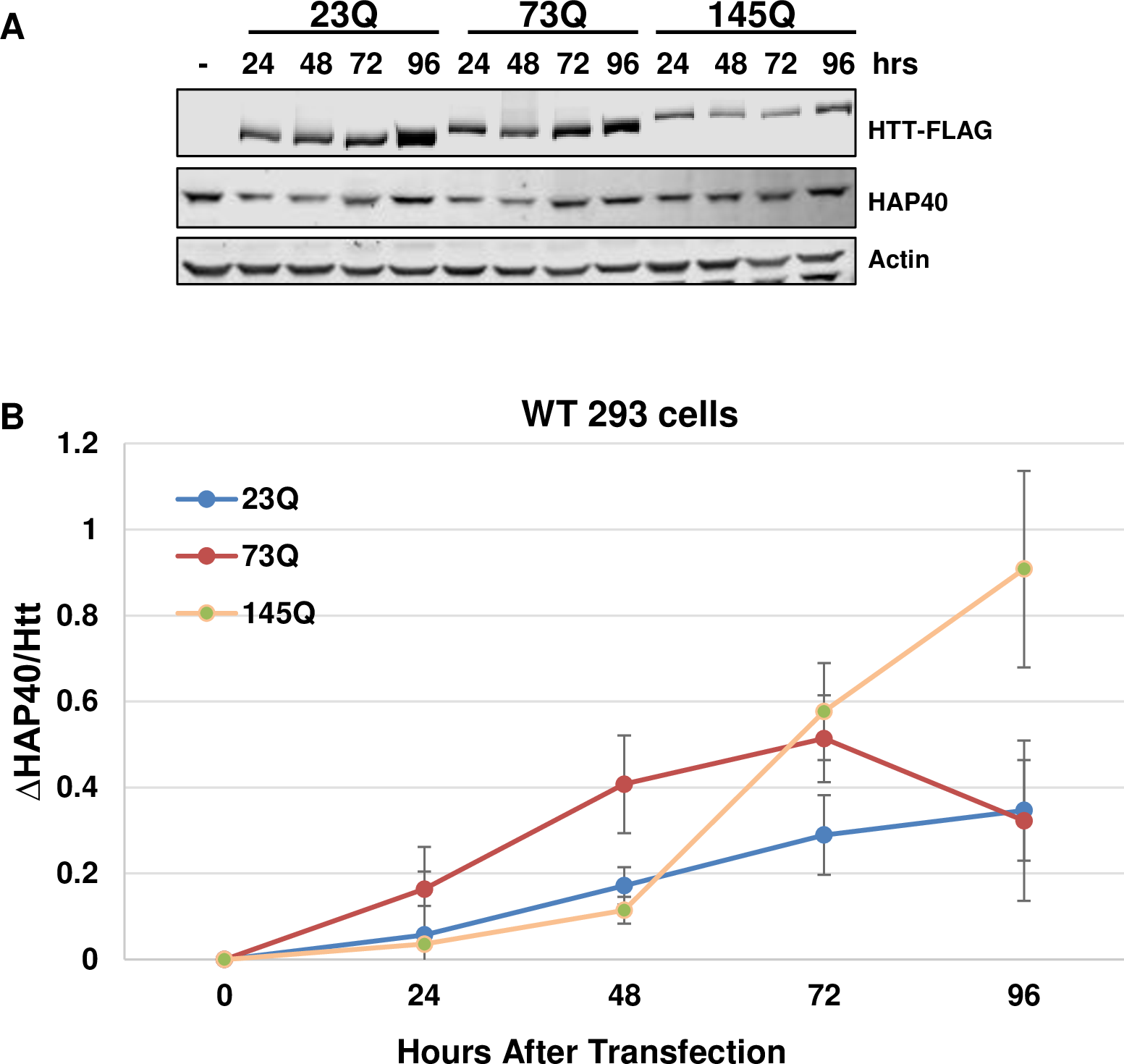

Supplement: S7 Fig — (A) Time course analyses of endogenous HAP40 and ectopically expressed HTT-23Q, 73Q or 145Q in wildtype HEK293 cells, hours after transfection with FLAG-tagged HTT expressing plasmids, as indicated. (B) Quantification of the time-dependent changes of HAP40 levels normalized against HTT at each time point, averaged from three repeat experiments. (TIF) [file pgen.1010302.s007.tif]

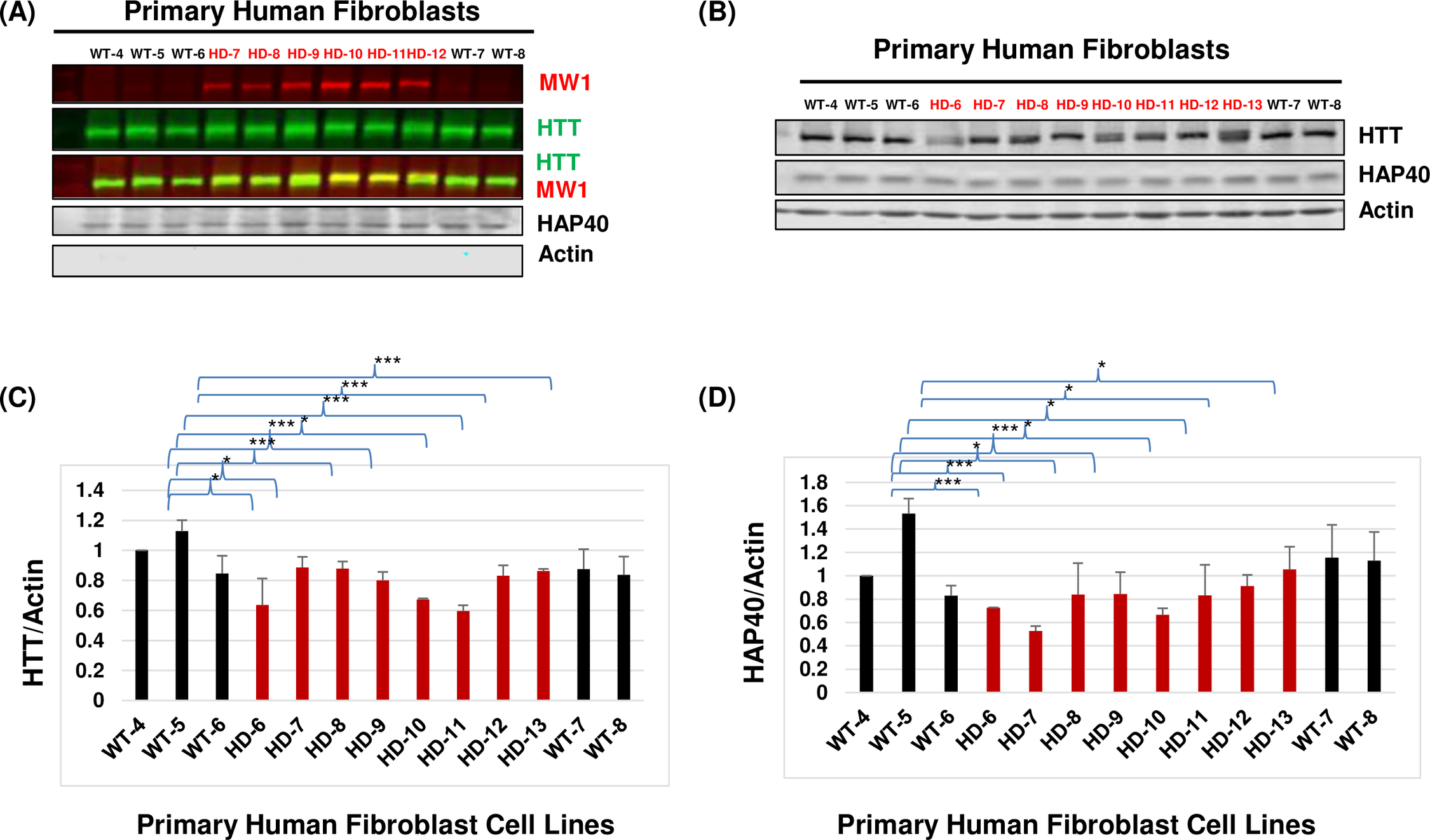

Supplement: S8 Fig — (A and B) Two independent Western blot assays of endogenous HTT and HAP40 proteins in additional five normal and eight HD human fibroblast cells, as indicated. In (A), the membrane was co-probed with both anti-total HTT (D7F7, green) and mutant HTT specific (MW1, red) antibodies. Note that in (A), MW1 detected mutant HTT bands in all the eight HD cell lines, but not in the five wildtype cell lines, and these mutant HTT bands were also positive for anti-total HTT (D7F7, green) antibody but several appeared to migrate slightly slower than wildtype HTT from the same cells. The human fibroblast cell lines tested are: GM02149 (WT-4), GM02153 (WT-5), GM02169 (WT-6), GM04190 (WT-7), GM04204 (WT-8), GM04687 (HD-6), GM04855 (HD-7), GM04691 (HD-8), GM04849 (HD-9), GM04737 (HD-10), GM03621 (HD-11), GM04857 (HD-12, CAG repeats reported as 50 and 40), and GM04281 (HD-13). (C and D) quantification of (C) endogenous HTT and (D) HAP40 levels in these cell lines, normalized against loading control Actin from three independent experiments. *p< = 0.05, **p< = 0.01, ***p< = 0.001 (student’s t-test). n.s., no significance. β-Actin served as loading and normalization controls in all the experiments. (TIF) [file pgen.1010302.s008.tif]

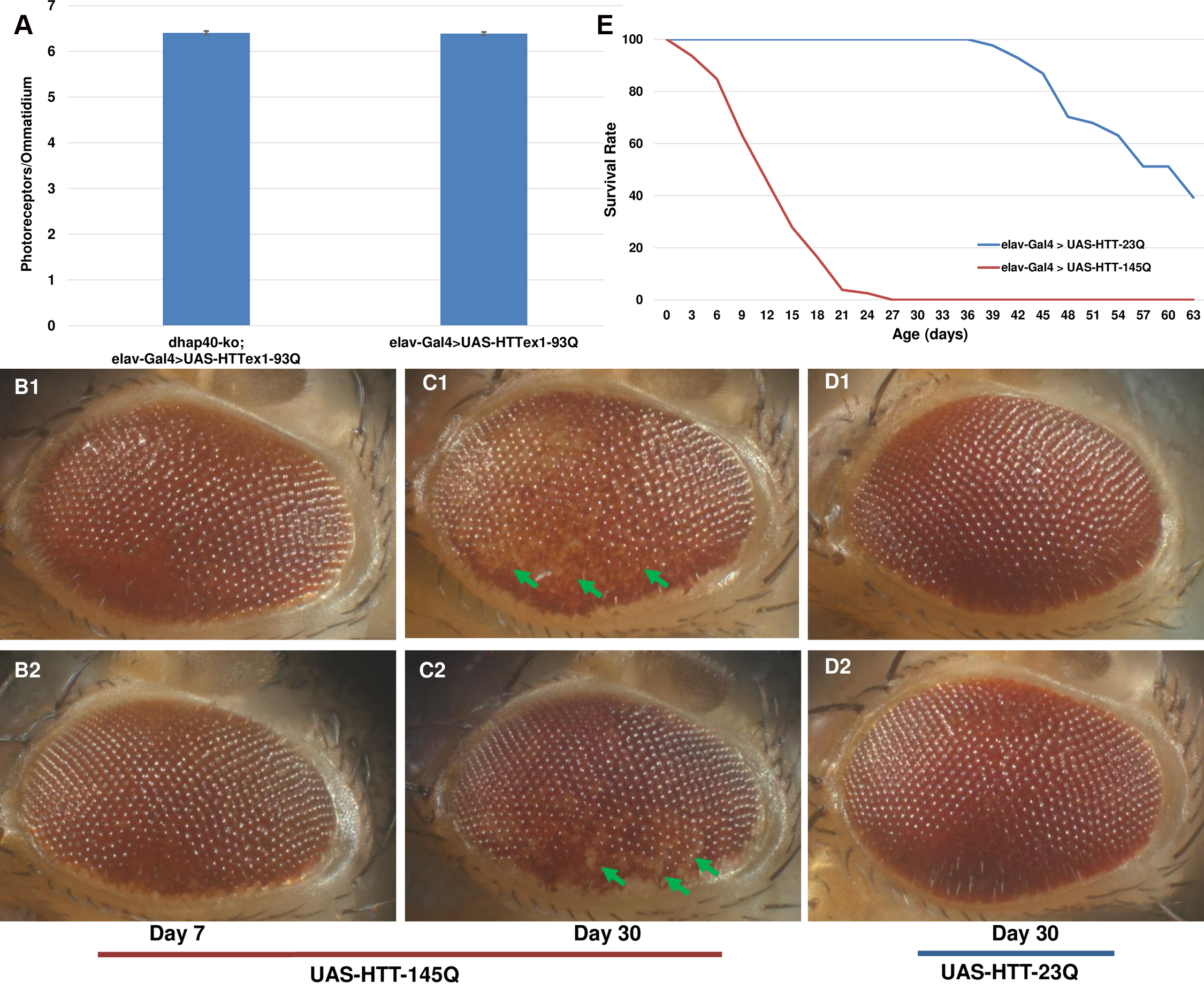

Supplement: S9 Fig — A. HAP40 does not modulate neurodegeneration induced by mutant HTT exon1 fragment in Drosophila. Loss of endogenous dhap40 did not have apparent effect on neurodegenerative phenotypes induced by HTT-exon1-93Q. Bar chart presentation of the average number of intact photoreceptor cells (PRC) per ommatidium in 7-day-old female flies of the following genotypes: “elav-Gal4/+; UAS-HTT-exon1-93Q/+”: 6.4 PRC/ommatidium (n = 12 flies); “dhap40ko3, elav-Gal4/+; UAS-HTT-exon1-93Q/+”: 6.4 PRC/ommatidium (n = 12 flies). The difference between the two is insignificant (p = 0.6 in rank-sum test). Flies in the study were cultured at 21°C. B-D. HTT-145Q induces age-dependent neurodegeneration in Drosophila eye. Bright-field images of adult fly eyes expressing full-length human HTT (HTT) with expanded (145Q, B and C) or wildtype (23Q, D) length of polyQ track, at young (7-day-old, B1 and B2) or old (30-day-old, C and D) ages, as indicated. Note the apparent de-pigmentation (green arrows), suggesting underneath cell death, only in old (C) but not young (B) flies expressing mutant HTT-145Q, and not in controls of wildtype HTT-23Q (D) at old age. Two representative flies for each indicated genotypes and ages were presented. Genotypes: (B and C) GMR-Gal4/+; UAS-HTT-145Q /+. (D) GMR-Gal4/+; UAS-HTT-23Q. More than 30 flies were examined for each genotypes. Note that the same S9D1 Fig eye image was also used in Fig 10D as control. E. Neuronal expression of HTT-Q145 but not HTT-Q23 induces neurodegeneration and causes early death of the animals. Survival curves of flies with pan-neuronal expression of full-length human HTT transgenes driven by pan-neuronal elav-Gal4 driver. Note that flies expressing mutant HTT (HTT-145Q, orange color) die significantly earlier than those expressing wildtype HTT (HTT-23Q, blue line). Genotypes: elav-Gal4/+; UAS-HTT-Q145 /+ (orange line, n = 79). elav-Gal4/+; UAS-HTT-23Q/+ (blue line, n = 84). (TIF) [file pgen.1010302.s009.tif]

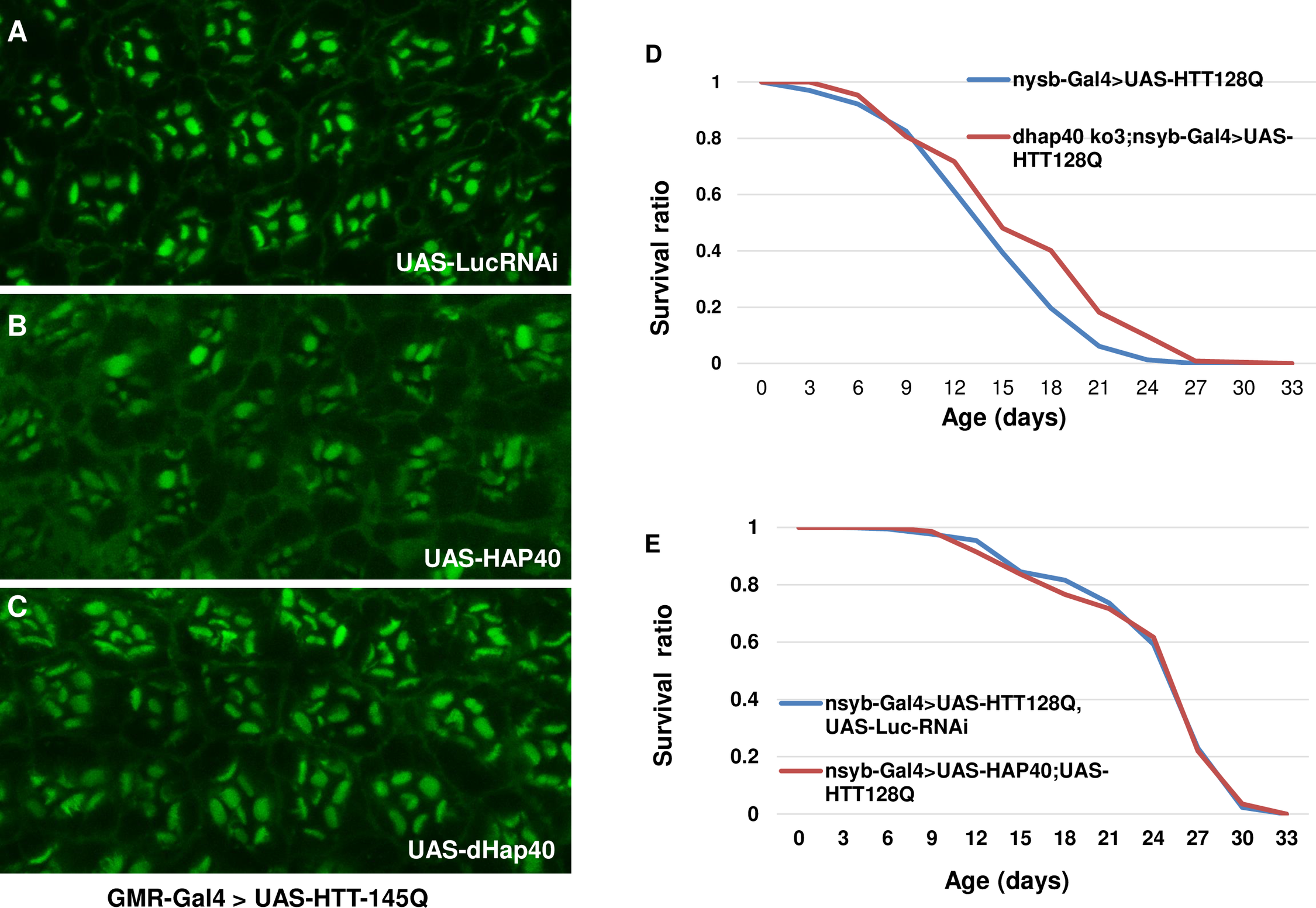

Supplement: S10 Fig — (A-C) Representative confocal images of whole-mount retina with phalloidin staining, dissected from 1-day-old adult flies with eye-specific co-expression of human mutant HTT-145Q with (A) control luciferase dsRNA, (B) human HAP40 or (C) fly dHap40, all directed by GMR-Gal4 driver. In control (A), co-expression of mutant HTT-145Q with luciferase dsRNA caused abnormal ommatidium units with elongated, fragmented and partial loss of rhabdomere structures in most ommatidia units. In contrast, (B) co-expression with human HAP40 significantly enhanced the degeneration phenotypes, showing significant loss of photoreceptor cells and more prominent deformation of ommatidia structure, while (C) co-expression with fly dHap40 did no significantly affect the phenotypes. Genotypes: (A) GMR-Gal4/+ >UAS-HTT-145Q/+; UAS-Luciferase dsRNA/+. (B) GMR-Gal4/+ >UAS-HTT-145Q/+; UAS-HAP40/+. (C) GMR-Gal4/+ >UAS-HTT-145Q/+; UAS-dHap40/+. (D and E) Survival curves of the adult flies expressing full-length human HTT-128Q. (D) Average life span was 17 days for “dhap40 ko3; nsyb-Gal4/UAS-HTT-128Q” flies (n = 179) and 15 days for control “nsyb-Gal4/UAS-HTT-128Q” flies (n = 229). The difference between the two is significance (p<0.001. Log-rank test). (E) Average life span was 24.3 days for flies co-expressing HTT-128Q with HAP40 (genotype: “nsyb-Gal4>UAS-HAP40/+; UAS-HTT-128Q/+”. n = 141), and 24.5 days for control flies co-expressing HTT-128Q with luciferase-dsRNA (“nsyb-Gal4> UAS-luciferase-RNAi/+; UAS-HTT-128Q/+”, n = 174), with no significant difference between the two genotypes (p = 0.9 by Log-rank test). (TIF) [file pgen.1010302.s010.tif]
