# Supplementary material for: HAP40 is a conserved central regulator of Huntingtin and a potential modulator of Huntington’s disease pathogenesis
Source: PLoS Genet. 2022 Jul 19;18(7):e1010302. doi: 10.1371/journal.pgen.1010302 (PMC9295956; doi:10.1371/journal.pgen.1010302)
Supplement: S2 Fig — A. The N- and C-terminal regions of HAP40 are most conserved between CG8134 (dHap40) and vertebrate HAP40. Sequence alignment of the N- and C-terminal regions between CG8134 and HAP40 homologs from human, mouse, frog and zebra fish, as indicated. The amino acid positions of the corresponding boundaries are labeled accordingly. Amino acids that are identical to CG8134 are highlighted in black and with similar chemical properties highlight in color. B. Sequence alignment between wildtype and dhap40 mutant alleles around the molecular lesions in established dhap40 alleles. C-F. The molecular lesions in four established dhap40 alleles. Genome sequence of exon 2 region of cg8134 gene, in which the exact molecular lesions of the four validated dhap40 mutant alleles, ko3, ko7, ko8 and ko9, are labeled in S2C-F, respectively, as indicated. (PDF) [file pgen.1010302.s002.pdf]

(A)

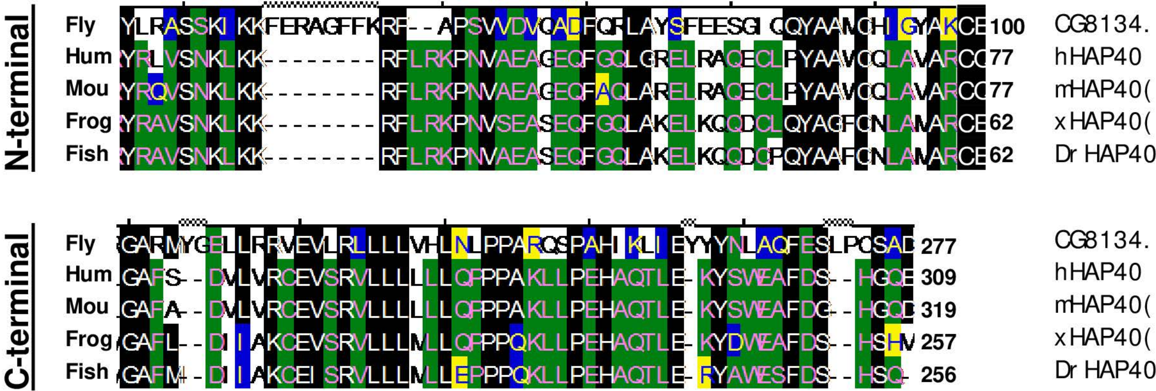
N-terminal
Fly
Hum
Mou
Frog
Fish
100
77
77
62
62
CG8134.
hHAP40
mHAP40(
xHAP40(
Dr HAP40
C-terminal
Fly
Hum
Mou
Frog
Fish
277
309
319
257
256
CG8134.
hHAP40
mHAP40(
xHAP40(
Dr HAP40

(B)

```
             Ser Tyr Gly Gly Ala Pro Gln Arg Glu Ser Glu Ala Tyr Leu Arg Ala Ala Arg Ser Phe   120
                        310                  320                 330                 340                 350                 360
CG8134 ORF   TCCTACGGCGGCGCACCGCAGCGCGAATCGGAGGCCTATCTGCGGGCAGCTAGATCCTTC   360
CG8134-ko3   TCCTACGGCGGCG-----------------------------------------------   313
CG8134-ko7   TCCTACGGCGGCGCACCGCAGCGCGAATCGGAGGCCTATCTGCGGGCAGCTAGATCC---   357
CG8134-ko8   TCCTACGGCGGCGCACCGCAGCGCGAATCGGAGGCCTATCTGCGGGCAGCTAGATCCT--   358
CG8134-ko9   TCCTACGGCGGCGCACCGCAGCGCGAATCGGAGGCCTATCTGCGG---------------   345

             Leu Ala Ala His Asn Glu Ser Gly Arg Leu His Leu Arg Thr Arg His Ser Gly Phe Arg   140
                        370                  380                 390                 400                 410                 420
CG8134 ORF   CTAGCGGCGCACAACGAGTCCGGGCGACTGCATCTGCGCACCCGCCACTCGGGATTTCGG   420
CG8134-ko3   ------------------------------------------------------------   313
CG8134-ko7   ---------------------GGGCGACTGCATCTGCGCACCCGCCACTCGGGATTTCGG   396
CG8134-ko8   ------------------------------------------------------------   358
CG8134-ko9   -------CGCACAACGAGTCCGGGCGACTGCATCTGCGCACCCGCCACTCGGGATTTCGG   398

             Glu Gly Ala Val His Cys Tyr His Arg Ala Ala Asp Arg Ala Val Asp Gly Cys Val Phe   160
                        430                  440                 450                 460                 470                 480
CG8134 ORF   GAGGGAGCGGTGCACTGCTATCACCGTGCGGCGGATCGAGCGGTGGACGGTTGCGTCTTC   480
CG8134-ko3   ---------------------------------GATCGAGCGGTGGACGGTTGCGTCTTC   340
CG8134-ko7   GAGGGAGCGGTGCACTGC-----------GGCGGATCGAGCGGTGGACGGTTGCGTCTTC   445
CG8134-ko8   ---------------------------GCGGCGGATCGAGCGGTGGACGGTTGCGTCTTC   391
CG8134-ko9   GAGGGAGCGGTGCACCGC--------------GGATCGAGCGGTGGACGGTTGCGTCTTC   444
```

(C)

Start point of deletion

end point of deletion

cgctttgcgcccagcgtggtcgatgtgcaggcggacttccagcgactggcctacagcttc
R F A P S V V D V Q A D F Q R L A Y S F
gaggagtcgggcatacagcagtatgcggccatgtgccacattggctatgccaagtgcgag
E E S G I Q Q Y A A M C H I G Y A K C E
tcctacggcggcgcaccgcagcgcgaatcggaggcctatctgcgggcagctagatccttc
S Y G G A P Q R E S E A Y L R A A R S F
ctagcggcgcacaacgagtccgggcgactgcatctgcgcacccgccactcgggatttcgg
L A A H N E S G R L H L R T R H S G F R
gagggagcggtgcactgctatcaccgtgcggcggatcgagcggtggacggttgcgtcttc
E G A V H C Y H R A A D R A V D G C V F
aaggcggccattctgcgcgagttgaagcagctgcagcggcagctggacagcaccagcagc
K A A I L R E L K Q L Q R Q L D S T S S
tttgcctcgcccacccatcagatccatgacctggaaatcagcgccgaaacgagtggccag
F A S P T H Q I H D L E I S A E T S G Q
cgcggcgattttcgcagtgcactgcagcattacgacgacattgtggacaatgtttacgag
R G D F R S A L Q H Y D D I V D N V Y E
cgacgcggtgctcgcatgtacggtgaactattgcggcgcgttgaggtgctgcgtctgctg
R R G A R M Y G E L L R R V E V L R L L
ctcctagtccatttgaacctgcctcccgcacgccagtcacccgcccacatcaagctgatc
L L V H L N L P P A R Q S P A H I K L I
gagtactactacaacctggcgcagttcgagtcgctcccctgttcggcggacgacggtgga
E Y Y Y N L A Q F E S L P C S A D D G G

(D)

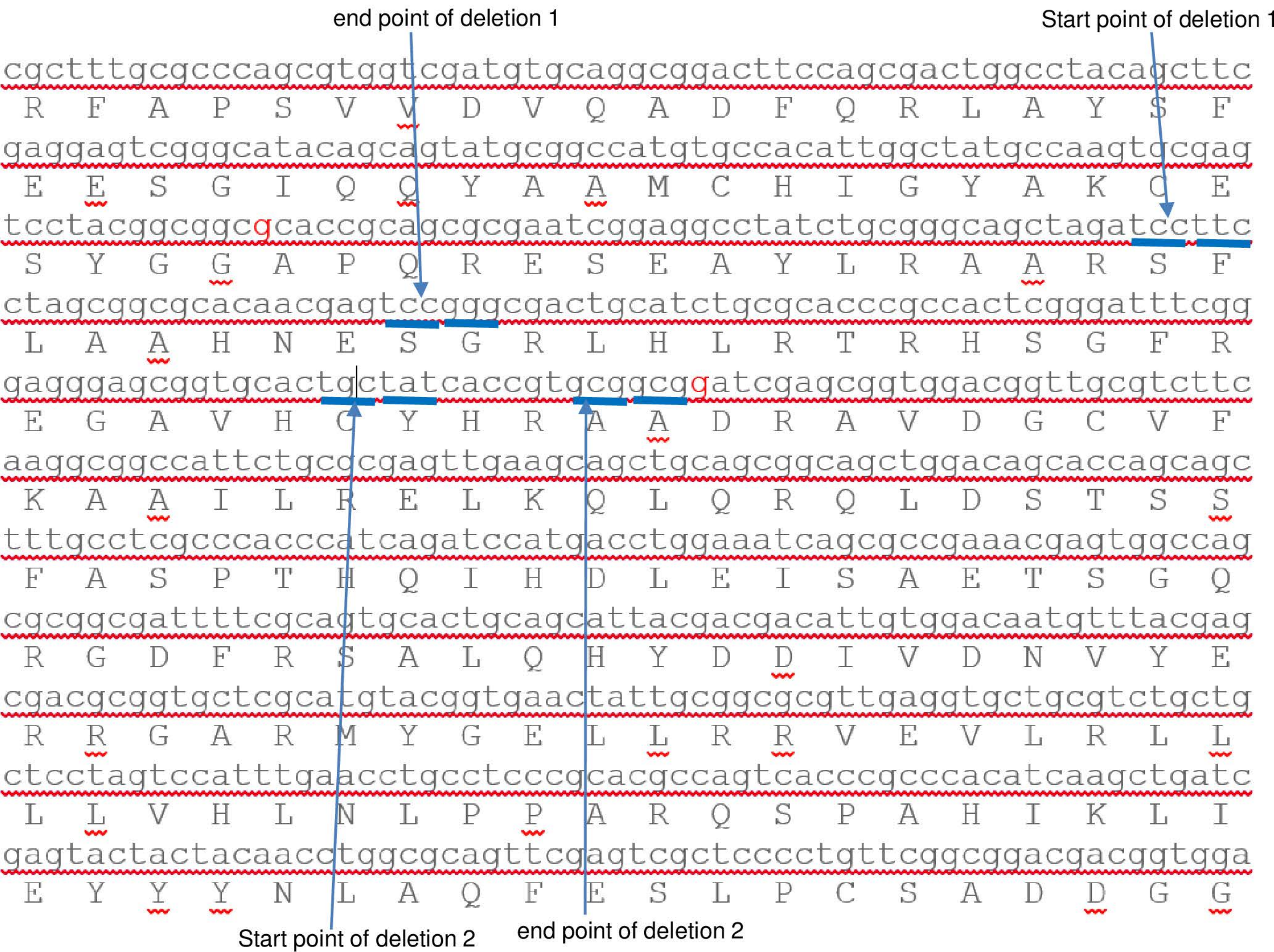
end point of deletion 1
Start point of deletion 1
cgctttgcgcccagcgtggtcgatgtgcaggcggacttccagcgactggcctacagcttc
R F A P S V V D V Q A D F Q R L A Y S F
gaggagtcgggcatacagcagtatgcggccatgtgccacattggctatgccaagtgcgag
E E S G I Q Q Y A A M C H I G Y A K C E
tcctacggcggcgcaccgcagcgcgaatcggaggcctatctgcgggcagctagatccttc
S Y G G A P Q R E S E A Y L R A A R S F
ctagcggcgcacaacgagtccgggcgactgcatctgcgcacccgccactcgggatttcgg
L A A H N E S G R L H L R T R H S G F R
gagggagcggtgcactgctatcaccgtgcggcggatcgagcggtggacggttgcgtcttc
E G A V H C Y H R A A D R A V D G C V F
aaggcggccattctgcgcgagttgaagcagctgcagcggcagctggacagcaccagcagc
K A A I L R E L K Q L Q R Q L D S T S S
tttgcctcgcccacccatcagatccatgacctggaaatcagcgccgaaacgagtggccag
F A S P T H Q I H D L E I S A E T S G Q
cgcggcgatttcgcagtgcactgcagcattacgacgacattgtggacaatgtttacgag
R G D F R S A L Q H Y D D I V D N V Y E
cgacgcggtgctcgcatgtacggtgaactattgcggcgcgttgaggtgctgcgtctgctg
R R G A R M Y G E L L R R V E V L R L L
ctcctagtccatttgaacctgcctcccgcacgccagtcacccgcccacatcaagctgatc
L L V H L N L P P A R Q S P A H I K L I
gagtactactacaacctggcgcagttcgagtcgctcccctgttcggcggacgacggtgga
E Y Y Y N L A Q F E S L P C S A D D G G
Start point of deletion 2
end point of deletion 2

(E)

end point of deletion

Start point of deletion

```
cgctttgcgcccagcgtggtcgatgtgcaggcggacttccagcgactggcctacagcttc
 R  F  A  P  S  V  V  D  V  Q  A  D  F  Q  R  L  A  Y  S  F
gaggagtcgggcatacagcagtatgcggccatgtgccacattggctatgccaagtgcgag
 E  E  S  G  I  Q  Q  Y  A  A  M  C  H  I  G  Y  A  K  C  E
tcctacggcggcgcaccgcagcgcgaatcggaggcctatctgcgggcagctagatccttc
 S  Y  G  G  A  P  Q  R  E  S  E  A  Y  L  R  A  A  R  S  F
ctagcggcgcacaacgagtccgggcgactgcatctgcgcacccgccactcgggatttcgg
 L  A  A  H  N  E  S  G  R  L  H  L  R  T  R  H  S  G  F  R
gagggagcggtgcactgctatcaccgtgcggcggatcgagcggtggacggttgcgtcttc
 E  G  A  V  H  C  Y  H  R  A  A  D  R  A  V  D  G  C  V  F
aaggcggccattctgcgcgagttgaagcagctgcagcggcagctggacagcaccagcagc
 K  A  A  I  L  R  E  L  K  Q  L  Q  R  Q  L  D  S  T  S  S
tttgcctcgcccacccatcagatccatgacctggaaatcagcgccgaaacgagtggccag
 F  A  S  P  T  H  Q  I  H  D  L  E  I  S  A  E  T  S  G  Q
cgcggcgatttcgcagtgcactgcagcattacgacgacattgtggacaatgtttacgag
 R  G  D  F  R  S  A  L  Q  H  Y  D  D  I  V  D  N  V  Y  E
cgacgcggtgctcgcatgtacggtgaactattgcggcgcgttgaggtgctgcgtctgctg
 R  R  G  A  R  M  Y  G  E  L  L  R  R  V  E  V  L  R  L  L
ctcctagtccatttgaacctgcctcccgcacgccagtcacccgcccacatcaagctgatc
 L  L  V  H  L  N  L  P  P  A  R  Q  S  P  A  H  I  K  L  I
gagtactactacaacctggcgcagttcgagtcgctcccctgttcggcggacgacggtgga
 E  Y  Y  Y  N  L  A  Q  F  E  S  L  P  C  S  A  D  D  G  G
```

(F)

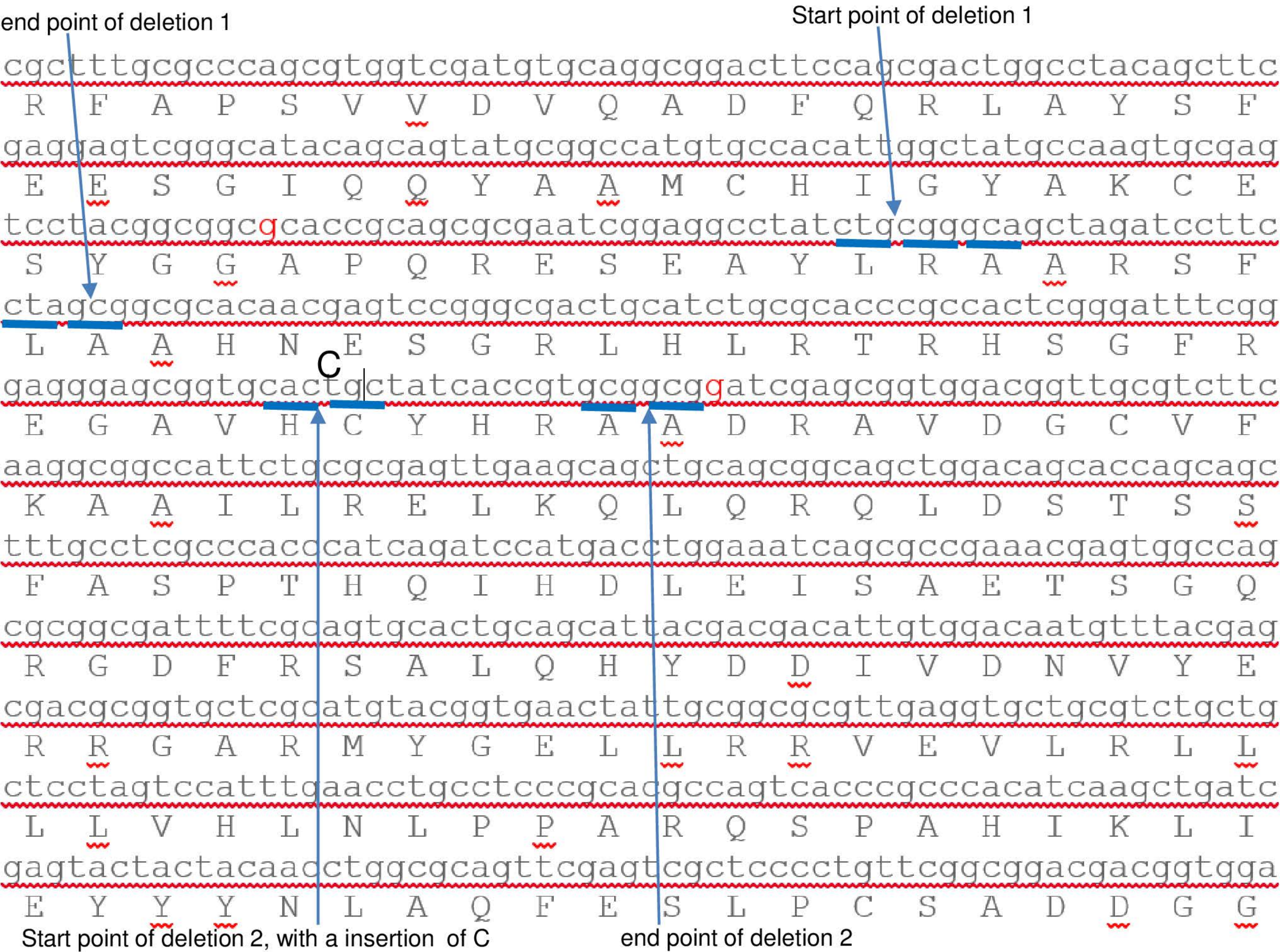
end point of deletion 1
Start point of deletion 1
cgctttgcgcccagcgtggtcgatgtgcaggcggacttccagcgactggcctacagcttc
R F A P S V V D V Q A D F Q R L A Y S F
gaggagtcgggcatacagcagtatgcggccatgtgccacattggctatgccaagtgcgag
E E S G I Q Q Y A A M C H I G Y A K C E
tcctacggcggcgcaccgcagcgcgaatcggaggcctatctgcgggcagctagatccttc
S Y G G A P Q R E S E A Y L R A A R S F
ctagcggcgcacaacgagtccgggcgactgcatctgcgcacccgccactcgggatttcgg
L A A H N E S G R L H L R T R H S G F R
C
gagggagcggtgcactgctatcaccgtgcggcggatcgagcggtggacggttgcgtcttc
E G A V H C Y H R A A D R A V D G C V F
aaggcggccattctgcgcgagttgaagcagctgcagcggcagctggacagcaccagcagc
K A A I L R E L K Q L Q R Q L D S T S S
tttgcctcgcccacccatcagatccatgacctggaaatcagcgccgaaacgagtggccag
F A S P T H Q I H D L E I S A E T S G Q
cgcggcgattttcgcagtgcactgcagcattacgacgacattgtggacaatgtttacgag
R G D F R S A L Q H Y D D I V D N V Y E
cgacgcggtgctcgcatgtacggtgaactattgcggcgcgttgaggtgctgcgtctgctg
R R G A R M Y G E L L R R V E V L R L L
ctcctagtccatttgaacctgcctcccgcacgccagtcacccgcccacatcaagctgatc
L L V H L N L P P A R Q S P A H I K L I
gagtactactacaacctggcgcagttcgagtcgctcccctgttcggcggacgacggtgga
E Y Y Y N L A Q F E S L P C S A D D G G
Start point of deletion 2, with a insertion of C
end point of deletion 2
